# Serelaxin as a potential treatment for renal dysfunction in cirrhosis: Preclinical evaluation and results of a randomized phase 2 trial

**DOI:** 10.1371/journal.pmed.1002248

**Published:** 2017-02-28

**Authors:** Victoria K. Snowdon, Neil J. Lachlan, Anna M. Hoy, Patrick W. F. Hadoke, Scott I. Semple, Dilip Patel, Will Mungall, Timothy J. Kendall, Adrian Thomson, Ross J. Lennen, Maurits A. Jansen, Carmel M. Moran, Antonella Pellicoro, Prakash Ramachandran, Isaac Shaw, Rebecca L. Aucott, Thomas Severin, Rajnish Saini, Judy Pak, Denise Yates, Neelesh Dongre, Jeremy S. Duffield, David J. Webb, John P. Iredale, Peter C. Hayes, Jonathan A. Fallowfield

**Affiliations:** 1 MRC Centre for Inflammation Research, University of Edinburgh, Edinburgh, United Kingdom; 2 British Heart Foundation/University of Edinburgh Centre for Cardiovascular Science, University of Edinburgh, Edinburgh, United Kingdom; 3 Clinical Research Imaging Centre, University of Edinburgh, Edinburgh, United Kingdom; 4 Department of Radiology, Royal Infirmary of Edinburgh, Edinburgh, United Kingdom; 5 Biological Services, University of Edinburgh, Edinburgh, United Kingdom; 6 Novartis Pharma, Basel, Switzerland; 7 Novartis Pharmaceuticals Corporation, East Hanover, New Jersey, United States of America; 8 Novartis Institutes for BioMedical Research, Cambridge, Massachusetts, United States of America; 9 Division of Nephrology and Lung Biology, University of Washington, Seattle, Washington, United States of America; Royal Derby Hospital, UNITED KINGDOM

## Abstract

**Background:**

Chronic liver scarring from any cause leads to cirrhosis, portal hypertension, and a progressive decline in renal blood flow and renal function. Extreme renal vasoconstriction characterizes hepatorenal syndrome, a functional and potentially reversible form of acute kidney injury in patients with advanced cirrhosis, but current therapy with systemic vasoconstrictors is ineffective in a substantial proportion of patients and is limited by ischemic adverse events. Serelaxin (recombinant human relaxin-2) is a peptide molecule with anti-fibrotic and vasoprotective properties that binds to relaxin family peptide receptor-1 (RXFP1) and has been shown to increase renal perfusion in healthy human volunteers. We hypothesized that serelaxin could ameliorate renal vasoconstriction and renal dysfunction in patients with cirrhosis and portal hypertension.

**Methods and findings:**

To establish preclinical proof of concept, we developed two independent rat models of cirrhosis that were characterized by progressive reduction in renal blood flow and glomerular filtration rate and showed evidence of renal endothelial dysfunction. We then set out to further explore and validate our hypothesis in a phase 2 randomized open-label parallel-group study in male and female patients with alcohol-related cirrhosis and portal hypertension. Forty patients were randomized 1:1 to treatment with serelaxin intravenous (i.v.) infusion (for 60 min at 80 μg/kg/d and then 60 min at 30 μg/kg/d) or terlipressin (single 2-mg i.v. bolus), and the regional hemodynamic effects were quantified by phase contrast magnetic resonance angiography at baseline and after 120 min. The primary endpoint was the change from baseline in total renal artery blood flow.

Therapeutic targeting of renal vasoconstriction with serelaxin in the rat models increased kidney perfusion, oxygenation, and function through reduction in renal vascular resistance, reversal of endothelial dysfunction, and increased activation of the AKT/eNOS/NO signaling pathway in the kidney. In the randomized clinical study, infusion of serelaxin for 120 min increased total renal arterial blood flow by 65% (95% CI 40%, 95%; *p <* 0.001) from baseline. Administration of serelaxin was safe and well tolerated, with no detrimental effect on systemic blood pressure or hepatic perfusion. The clinical study’s main limitations were the relatively small sample size and stable, well-compensated population.

**Conclusions:**

Our mechanistic findings in rat models and exploratory study in human cirrhosis suggest the therapeutic potential of selective renal vasodilation using serelaxin as a new treatment for renal dysfunction in cirrhosis, although further validation in patients with more advanced cirrhosis and renal dysfunction is required.

**Trial registration:**

ClinicalTrials.gov NCT01640964

## Abstract 2

In a Research Article, Jonathan Fallowfield and colleagues study serelaxin as a possible treatment for renal vasoconstriction in liver cirrhosis.

## Author summary

**Why was this study done?:**

- Hepatorenal syndrome type 1 is a rapidly progressive, but potentially reversible, form of acute kidney injury occurring in patients with liver cirrhosis, characterized by severe renal vasoconstriction and a very poor prognosis. There is a significant unmet need for a widely approved, safe, and effective pharmacological treatment.
- Serelaxin, a recombinant form of the human peptide hormone relaxin-2, has been shown to increase renal perfusion in healthy human volunteers. We tested whether serelaxin could ameliorate renal vasoconstriction and renal dysfunction in cirrhosis.

**What did the researchers do and find?:**

- Administration of serelaxin improved renal blood flow, oxygenation, and function in two independent animal models of cirrhosis through reversal of endothelial dysfunction and increased activation of nitric oxide signaling in the kidney.
- In an exploratory phase 2 clinical trial in patients with cirrhosis and portal hypertension, serelaxin infusion induced a significant increase in renal blood flow and was safe and well tolerated, with no adverse effects on systemic blood pressure or hepatic perfusion.

**What do these findings mean?:**

- Our data indicate that selective targeting of renal vasoconstriction using serelaxin may modulate renal dysfunction in cirrhosis without affecting systemic blood pressure.
- Further studies in patients with more advanced cirrhosis and renal dysfunction are required to confirm whether there are beneficial effects on renal function and patient outcomes.

## Introduction

Acute kidney injury (AKI) is a major challenge for healthcare providers and clinicians worldwide, affecting an estimated 13 million people and contributing to 1.7 million deaths every year [1]. AKI occurs in approximately 20% of patients with liver cirrhosis who are admitted into hospital, and the associated morbidity and mortality remain unacceptably high [2]. Hepatorenal syndrome (HRS), the most severe form of AKI in cirrhosis, develops in more than 50% of patients with cirrhosis who die [3]. HRS is a functional, and potentially reversible, type of renal failure characterized by intense renal vasoconstriction and hypoperfusion and is associated with a dismal prognosis [4]. It occurs in response to portal hypertension (PHT) and the associated splanchnic arterial vasodilatation and impairment of cardiac function that lead to a reduction in effective circulating volume, with compensatory activation of neurohormonal systems and an intrarenal imbalance between vasoconstrictor and vasodilator systems causing increased renal vascular resistance (RVR) [5,6]. Pooling of blood in the splanchnic circulation also alters gut permeability and enhances bacterial translocation, with the release of endotoxin and increase in pro-inflammatory cytokines leading to amplification of circulatory dysfunction [7]. Imaging studies have shown a significant (~40%) reduction in renal blood flow (RBF) and redistribution of intrarenal blood flow in patients with compensated cirrhosis compared to healthy volunteers [8,9]. As cirrhosis advances, systemic hemodynamics are increasingly disturbed, RBF becomes progressively compromised, and patients are susceptible to episodes of AKI (including HRS) in response to precipitating factors, particularly bacterial infections [10].

The median survival time for patients with the acute form of HRS (“type 1”) is only 4 wk, highlighting the need for an effective pharmacological treatment [11]. Evidence also suggests that even relatively mild cases of AKI, insufficient to make a diagnosis of HRS, have a negative impact on patient survival, indicating that prompt and successful treatment of these episodes could lead to improved clinical outcomes [12]. The most effective pharmacological treatment for HRS type 1 is terlipressin (a splanchnic and systemic vasoconstrictor). However, in a recent large randomized placebo-controlled trial, confirmed HRS reversal occurred in only 19.6% of patients receiving terlipressin, and the drug was permanently discontinued in 20.4% due to adverse (mainly ischemic) events [13]. An ideal treatment for HRS would consist of a drug with selective vasodilator activity in the renal circulation but without significant vasodilator effects in other vascular beds, particularly the splanchnic circulation.

The endogenous peptide hormone relaxin (RLX) regulates maternal hemodynamic adaptations in early pregnancy through direct actions on the renal and systemic vasculature, including reduced RVR, increased RBF, and increased glomerular filtration rate (GFR) [14]. Serelaxin (recombinant human relaxin-2) binds with high affinity to the G-protein-coupled receptor RXFP1, which is differentially expressed in the renal and systemic vasculature [15] and mediates vasoactive effects through a number of mechanisms including enhanced nitric oxide (NO) signaling and antagonism of the vasoconstrictive effects of angiotensin-II and endothelin [14]. However, in contrast to vasodilators such as nitroglycerin, which primarily act via direct venodilation, the vasorelaxatory action of serelaxin in rats is thought to predominantly affect arteries [16]. We recently showed that administration of serelaxin in cirrhotic rats decreased PHT through augmentation of hepatic NO signaling and a reduction in intrahepatic vascular resistance [17]. In healthy human volunteers, intravenous infusion of serelaxin for 30 min increased RBF by 47% compared with baseline levels, irrespective of sex [18].

We hypothesized that serelaxin could ameliorate renal vasoconstriction and renal dysfunction in cirrhosis. To test our hypothesis, we first evaluated the effects of serelaxin in two independent experimental rat models of cirrhosis that were characterized by a progressive reduction in RBF and GFR and showed evidence of renal endothelial dysfunction. We then determined the translational relevance of these findings in an exploratory phase 2 clinical trial in patients with compensated cirrhosis and PHT who were randomized to treatment with serelaxin or terlipressin, followed by noninvasive hemodynamic assessments using magnetic resonance imaging (MRI).

## Methods

### Experimental design

Our main objective was to use experimental and human models to establish proof of concept for serelaxin in modulating renal vasoconstriction and thereby its potential as a novel clinical therapy for renal dysfunction in cirrhosis.

Animal experiments were designed and implemented according to NC3Rs ARRIVE guidelines. We calculated sample sizes prospectively for the animal model experiments using our previously published data (group means and standard deviations) on using serelaxin in rat cirrhosis induced by carbon tetrachloride (CCl_4_) and bile duct ligation (BDL) [17]. A sample size of ≥5 rats per group was computed using G*Power version 3.1.2 [19] given α = 0.05, power (1 − β) = 0.9, and effect size (*d*) = 2.64. Cage-mates were randomized to serelaxin or vehicle treatment, and all surviving rats at designated endpoints were included in the data analysis. No animals or potential outliers were excluded from the datasets presented. Biochemical and histological assessments were performed in a blinded manner.

In addition, a phase 2 randomized clinical trial (ClinicalTrials.gov NCT01640964) was conducted. The main purpose of this study was to investigate the hemodynamic effects of serelaxin infusion on the renal and hepatic circulation, measured by phase contrast magnetic resonance angiography (PC-MRA), in patients with compensated alcohol-related cirrhosis and PHT. Terlipressin, a well-characterized drug licensed in many countries for treatment of variceal bleeding and HRS, was used as a positive control to validate the sensitivity and dynamic range of the PC-MRA methodology in this population. The study was not sized based on the power to detect a difference between the serelaxin and terlipressin groups. Instead, a sample size of 20 patients per group was calculated to be sufficient to create a 90% confidence interval (CI) on the mean change from baseline for all blood flow parameters that would exclude zero if a 25% change was observed.

### Ethics statement

Procedures involving animals were conducted with approval from the University of Edinburgh Animal Welfare and Ethical Review Body and UK Home Office, and in compliance with the Animals (Scientific Procedures) Act 1986. Wherever possible, inhalational anesthesia was used to maximize control of depth/duration of anesthesia. Animals were killed by an approved Schedule 1 method by appropriately trained personnel. Human tissues were analyzed to validate preclinical observations in accordance with the Human Tissue (Scotland) Act 2006. The clinical trial was conducted according to the ethical principles of the Declaration of Helsinki 2013 and following approval from the Scotland A Research Ethics Committee (REC Ref: 12/SS/0177). All patients gave written informed consent.

### Rat carbon tetrachloride and bile duct ligation models

Animals were housed under standard conditions (12:12 h light-dark cycle, temperature 23 ± 2°C), three rats per cage, and were acclimatized to the room for 1 wk before the beginning of the experiments. Cirrhosis and PHT were induced in 8-wk (250–300 g) male wild-type Sprague-Dawley rats by intraperitoneal CCl_4_ (Sigma-Aldrich) treatment for up to 16 wk or by BDL for up to 4 wk, as described previously [17]. Cage-mates were randomly allocated to CCl_4_ versus olive oil (OO) vehicle treatment or to BDL versus sham procedure (where the common bile duct was exposed but not ligated).

### Sustained (72 h) serelaxin hemodynamic study

Both 16-wk CCl_4_ (*n =* 16) and 3-wk post-BDL (*n =* 14) rats were randomized to receive continuous treatment for 72 h with serelaxin (4 μg/h) or vehicle via subcutaneous (s.c.) osmotic minipumps (2ML1 Alzet, Durect). Groups of CCl_4_ rats (*n =* 12) were also randomized to serelaxin or vehicle plus coadministration of L-N^G^-nitroarginine methyl ester (L-NAME) (Sigma-Aldrich) 250 mg/ml in drinking water, starting 24 h before osmotic minipump insertion and continuing for the 72-h study period. After 72 h, rats had hemodynamic and renal functional measurements performed under terminal isoflurane anesthesia, as detailed below.

### Acute serelaxin hemodynamic study

Serelaxin was supplied under material transfer agreement by Corthera (a Novartis affiliate company). Groups of OO control rats (*n =* 12) or 16-wk CCl_4_ rats (*n =* 14) were randomized to injection of serelaxin (4 μg in 200 μl intravenous [i.v.]) or an equivalent volume of vehicle, and anesthetized for continuous hemodynamic monitoring over 60 min.

### Acute nitrovasodilator hemodynamic study

Groups of 16-wk CCl_4_ rats (*n =* 6) were anesthetized and treated with a range of doses of sodium nitroprusside (SNP; 0.09, 0.9, and 9 μg in 200 μl i.v.; Sigma-Aldrich) or an equivalent volume of vehicle. Continuous hemodynamic monitoring was undertaken for 10 min after each dose, as described above.

### Hemodynamic measurements

Hemodynamic monitoring was performed using aseptic technique in rats anesthetized with Inactin (Sigma-Aldrich) 0.1 ml/100 g (125 mg/ml) unless otherwise specified. Briefly, a tracheostomy was created, the right internal jugular vein cannulated with PE50 tubing (Smiths Medical), and 0.9% NaCl instilled at 0.05 ml/100 g/min throughout surgery and then 0.03 ml/100 g/min during stabilization/monitoring. Rats received 5% inulin-FITC (Sigma-Aldrich) in 0.9% NaCl for GFR measurement. Body temperature was maintained at 37°C using a heat mat. Mean arterial pressure (MAP) and heart rate (HR) were measured via a right femoral artery PE50 catheter, portal pressure (PP) using a 24G cannula inserted into the portal vein, and RBF using a Doppler transit time probe (MA2PSB, ADInstruments) placed around the left renal artery proximal to the bifurcation, all connected to a Powerlab 4/35 system and analyzed using LabChart 7 Pro software (ADInstruments). Rats were stabilized for 20 min prior to data recording. The urinary bladder was catheterized with PE90 tubing. At the end of the experiment, blood was collected and tissues fixed in 10% buffered formalin or snap frozen.

### Glomerular filtration rate measurement

A continuous infusion of 5% inulin-FITC in 0.9% NaCl was instilled at 0.03 ml/100 g/min. Two 20-min urine collections were performed after 20 min of stabilization, and plasma samples were collected at baseline, 20 min, and 40 min for each rat and frozen immediately on dry ice. Samples were thawed and spun down before analysis. Urine was diluted 1:100 in HEPES buffer (HEPES 1.19 g, 450 ml of distilled water, pH adjusted to 7.4 with 5 M NaOH, and autoclaved). A nine-point 2-fold dilution series was constructed starting with 1 mg/ml in HEPES buffer (20 μl in 980 μl of buffer). All samples were further diluted 1:100 in HEPES buffer and were analyzed in duplicate. Then, 190 μl was loaded per well, and fluorescence read at 485 nm (excitation) and 538 nm (emission). Concentrations of inulin were calculated from the standard curve. The urine volume and urine and plasma concentrations of inulin were measured. The GFR was calculated as follows: [(urine inulin × urine volume/min)/plasma inulin]. The mean plasma inulin concentration was 0.18 mg/ml.

### Wire myography

Wire myography was performed using isolated renal vessels as described previously [20]. Vascular responses were studied in vessels from 16-wk OO/CCl_4_ rats (*n =* 6) using physiological inhibitors of NO, cyclooxygenase, and endothelium-derived hyperpolarizing factor (EDHF) signaling, and in groups of 4-wk sham/BDL rats and 16-wk OO/CCl_4_ rats after 72-h serelaxin or vehicle infusion (*n =* 6/8 per group). Harvested vessels (extrarenal and intrarenal renal arteries) were placed in 4°C physiological salt solution (PSS) (119 mM NaCl, 4.7 mM KCl, 2.5 mM CaCl_2_, 1.17 mM MgSO_4_, 25 mM NaHCO_3_, 1.18 mM KH_2_PO_4_, 0.026 mM K_2_EDTA, and 5.5 mM D-glucose). Extrarenal, segmental, and interlobar renal arteries were dissected and cleaned of connective tissue. Rings of 2 mm were mounted from each vessel segment onto 40-mm stainless steel wires, one end of which was attached to a force transducer, and the other to a micrometer. The vessel ring was bathed in PSS at 37°C and bubbled with 95% O_2_ and 5% CO_2_, and stepwise radial stretching was performed to determine the lumen diameter necessary for optimal force generation. Vessel rings were then stretched to achieve 90% of the diameter expected if they had been fully relaxed and exposed to a transmural pressure of 13.3 kPa (100 mm Hg). The vessel ring was allowed to equilibrate for 30 min before viability was assessed using three stimulations with KPSS (125 mM K, equimolar substitution of NaCl with KCl in PSS), then activation for 2 min with each solution, followed by a 5-min washout period in PSS to allow full relaxation. Measurement of viability was performed in these vessels to confirm that isolation and mounting of the vessel did not damage the arterial wall. The ability of the vessel to respond to contractile and dilator agents was investigated by producing concentration–response curves (CRCs). CRCs were obtained for phenylephrine (1 × 10^−9^ to 3 × 10^−5^ M; Sigma-Aldrich), and a concentration that produced 80% maximum contraction (EC_80_) was chosen for each individual rat vessel ring. Following contraction, CRCs were obtained to the endothelium-dependent vasodilator acetylcholine (ACh; 1 × 10^−9^ to 3 × 10^−5^ M; Sigma-Aldrich) and the endothelium-independent vasodilator SNP (1 × 10^−9^ to 3 × 10^−5^ M; Sigma-Aldrich). To determine the mechanism underlying physiological endothelium-dependent vasodilatation in isolated normal/control rat renal arteries, vessels were pretreated with either the NO synthase (NOS) inhibitor L-NAME (1 × 10^−4^ M; Sigma-Aldrich), the cyclooxygenase (COX) inhibitor indomethacin (1 × 10^−5^ M; Sigma-Aldrich), or the endothelium-derived hyperpolarizing factor inhibitors apamin (1 × 10^−4^ M; Sigma-Aldrich) and charybdotoxin (1 × 10^−5^ M; Sigma-Aldrich) before stimulation with ACh.

### Kidney blood-oxygen-level-dependent magnetic resonance imaging studies

Groups of 8-wk (*n =* 16) and 16-wk CCl_4_-treated rats (*n =* 12) were randomized to serelaxin (4 μg in 200 μl i.v.) or vehicle (200 μl), and kidney blood-oxygen-level-dependent MRI (BOLD-MRI) measurements (T2*) were acquired using a 7.0 Tesla preclinical MRI scanner (Agilent Technologies).

Animals were anesthetized with isoflurane, and the tail vein was cannulated for serelaxin or vehicle injection. Core body temperature was maintained at 37°C, and respiratory rate was recorded throughout the scanning protocol. A birdcage volume coil (72-mm diameter) and a four-channel phased array surface coil (Rapid Biomedical) were used for radio frequency transmission and signal reception, respectively. BOLD-MRI image acquisition used a multiecho gradient-recalled echo pulse sequence of ten T2* weighted images; TE = 4, 8, 12, 16, 20, 24, 28, 32, 36, and 40 ms; TR = 100 ms; and flip angle of 30°. An axial slice through the center of the right kidney was selected with 50 × 40 mm field of view containing a 192 × 128 acquisition matrix (in-plane resolution = 0.26 × 0.31 mm). Selecting a single axial slice aligned parallel with the renal artery, identified by rapid scout scanning (fast gradient echo, three slices in coronal orientation), ensured that the slice position encompassed the most representative section of the kidney regions. Slice thickness was 2 mm, with 14 signal averages. The scan time was 3 min for each BOLD-MRI scan.

### High-resolution ultrasound studies

Groups of 16-wk CCl_4_ rats (*n =* 16) were randomized to serelaxin (4 μg in 200 μl i.v.) or vehicle (200 μl), and RBF and cardiac output (CO) were assessed by ultrasound (Vevo 770, VisualSonics).

Animals were anesthetized with isoflurane, placed in a supine position on a heated table (VisualSonics, Fujifilm), and cannulated via tail vein for injection of serelaxin or vehicle. Heart rate was monitored continuously using an electrocardiogram, and core body temperature was maintained at 37°C using the heated table and a heat lamp as required. The chest and abdominal regions were shaved and remaining hair removed with depilatory cream. Parasternal, long-axis B-mode and M-mode images were taken of the heart at the level of the papillary muscle for quantitative assessment of cardiac function at baseline and at 60 min after injection of serelaxin or vehicle. Measurements included end systolic and end diastolic left ventricular area and volume, stroke volume, CO, and ejection fraction. A short-axis image of the right kidney was then obtained, and, using pulse-wave Doppler, measurements of RBF were taken from the superior branch of the renal artery distal to the point of renal artery bifurcation at the hilum. Measurement of renal artery Doppler ultrasound parameters (peak velocity, velocity time integral, and Pourcelot resistive index [(peak systolic velocity − end diastolic velocity)/peak systolic velocity]) were taken at baseline and every 10 min for 60 min.

### Immunofluorescence

Kidney tissue sections (7 μm) were incubated with primary anti-RXFP1 (H-160) antibody (Santa Cruz Biotechnology, sc-50328; 1:200 rat, 1:500 human) and detected with ImmPRESS HRP Anti-Rabbit IgG (Vector Laboratories, MP-7401). The HRP label was amplified with Cy3-conjugated tyramine (PerkinElmer, SAT704B001EA).

### Determination of hepatic collagen proportionate area

Liver tissue sections (3 μm) from four separate lobes were stained with picrosirius red, and collagen proportionate area (percent) calculated as described previously [17].

### Quantification of renal acute tubular necrosis

Hematoxylin-and-eosin-stained kidney sections were assessed for acute tubular necrosis (ATN) by a histopathologist blinded to treatment allocation. Ten high-power fields (×400) were analyzed for the percentage of tubules showing cell necrosis, loss of brush border, cast formation, and tubule dilatation and scored as follows: 0, none; 1, ≤10%; 2, 11%–25%; 3, 26%–45%; 4, 46%–75%; and 5, >76%.

### Reverse transcription and real-time PCR

RNA extraction, reverse transcription, and real-time PCR were performed as described previously [17] using validated primer/probe sets for rat RXFP1 (Rn01495351_m1) and eukaryotic 18S (Applied Biosystems) or the Rat Hypertension RT^2^ Profiler PCR Array (Qiagen). Gene expression was normalized to 18S rRNA, and relative expression calculated by 2^−ΔΔ^Ct method.

### Human relaxin-2 and TNFα measurement

Rat serum was diluted 1:4 before human relaxin-2 or TNFα levels were determined using solid-phase Quantikine ELISA (R&D Systems).

### Nitric oxide synthase assay

Whole kidney tissue was homogenized in phosphate-buffered saline (pH 7.4) and centrifuged at 10,000*g* for 10 min, and supernatant collected. Then, 150 μg of protein was used to measure NOS activity using an EnzyChrom kit (BioAssay Systems).

### Nitric oxide measurement

Rat serum was centrifuged (13,000*g*, 2 min) before filtration using a 10,000-Da molecular weight cutoff filter (Millipore). NO/NO_3_^−^/NO_2_ determination was performed using the Parameter Total Nitric Oxide and Nitrate/Nitrite Assay Kit (R&D Systems).

### Western blotting

Western blotting was performed as described previously [17] using the following primary antibodies/dilutions: panAKT, 1:1,000; phospho-AKT^Thr308^, 1:1,000; eNOS, 1:1,000; phospho-eNOS^Ser117^, 1:1,000 (all Cell Signaling Technology); relaxin receptor 1 H160, 1:1,000 (Santa Cruz Biotechnology). Densitometry was performed using ImageJ software (US National Institutes of Health).

### Clinical trial

We undertook a randomized open-label non-controlled parallel-group exploratory phase 2 clinical trial in *n =* 40 patients recruited at the Royal Infirmary of Edinburgh (Edinburgh, UK) between April 2013 and October 2014 (Protocol: CRLX030X2201, version 01; see S1 Text).

#### Inclusion/exclusion criteria

The following inclusion criteria applied: age 18–75 y, male or female, and cirrhosis of alcohol etiology with clinical and/or endoscopic evidence of PHT. Key exclusion criteria included the following: use or plan for use of any drug to treat PHT (e.g., nonselective beta blockers), decompensated cirrhosis (Child-Pugh score > 9 at screening) and/or ascites and/or hepatic encephalopathy, variceal bleed in the preceding month, ongoing drug or alcohol abuse, severe renal impairment (estimated GFR < 30 ml/min), systolic blood pressure < 110 mm Hg, hepatocellular carcinoma, known portal or splenic vein thrombosis, prolonged corrected QT interval, contraindication to MRI scan, contraindication to terlipressin, and any uncontrolled clinically significant disease.

#### Interventions

After written informed consent was obtained, patients underwent three study visits: screening, treatment, and follow-up at −7, 0, and 29 d, respectively. Eligible patients were randomly assigned (1:1) to treatment with either (1) i.v. infusion of serelaxin at an initial rate of 80 μg/kg/d (equivalent to 3.33 μg/kg/h) for the first 60 min, followed by a subsequent rate of 30 μg/kg/d (equivalent to 1.25 μg/kg/h) for the next 60 min or (2) a single i.v. bolus of 2 mg of terlipressin acetate. Participants were monitored for 120 min after the initiation of treatment. We used a pragmatic 120-min time frame as pharmacokinetic studies had indicated that steady-state serum serelaxin levels efficacious in a phase 3 acute heart failure trial could be achieved within this period [21] and the marked hemodynamic effects of i.v. terlipressin persist for up to 4 h [22]. The study had a 4-wk follow-up time point because this was considered the best interval for detecting anti-serelaxin antibody development.

#### Randomization

Participants were randomized using computer-generated allocation cards. The patient randomization list was produced by Novartis Drug Supply Management using a validated automated system. The randomization scheme was reviewed and approved by a member of the Novartis Biostatistics Quality Assurance Group.

#### Outcome measures

The primary outcome was the change from baseline in the total renal artery blood flow following serelaxin treatment (where the flow is the average flow over the cardiac cycle, and total renal artery flow = left + right renal artery flow measured by PC-MRA). Secondary outcomes were as follows: the change from baseline in total renal artery flow 120 min after terlipressin treatment; the change in blood flow from baseline following serelaxin treatment in the portal vein, hepatic artery, superior mesenteric artery (SMA), and superior abdominal aorta (above renal bifurcation); and the number of patients with adverse events (AEs), serious adverse events (SAEs), and death, as assessment of safety and tolerability of serelaxin over the 4-wk study period. Prespecified exploratory outcomes included the change in blood flow from baseline following serelaxin treatment in the inferior abdominal aorta (below renal bifurcation) and the azygos vein, and the effects of serelaxin treatment on selected biomarkers, pharmacokinetics, and immunogenicity.

#### Study assessments

For PC-MRA, a 3.0 Tesla whole body MRI scanner using the Verio platform (Siemens Healthcare) equipped with spine and body receiver array coils was used to acquire ECG-gated noncontrast magnetic resonance angiography using 3-D gradient echo (FLASH) sequences and 2-D true fast imaging with steady-state precession (FISP) for localization of the hepatorenal vasculature. The phase images from the 3-D PC-MRA sequences were analyzed before and after serelaxin or terlipressin treatment (S10 Fig). Outcome assessors for PC-MRA were blinded to treatment allocation. For each vessel, the velocity encoding of the acquisition was adjusted on a patient-by-patient basis. All images were checked visually after each flow measurement sequence; if motion artifacts were detected, the individual vessel scan was repeated. Flow measurements were derived from a semi-automated analysis of phase contrast images using Siemens Argus Flow software. On the vascular images where the PC-MRA phase sequence had been applied, contours were drawn manually (or semi-automatically after seed points were indicated at the lumen boundary) at end diastole (first image of the cine acquisition) and end systole (smallest cavity size during cardiac cycle) on every slice. For the flow measurements, the lumen of each vessel was carefully evaluated at the peak systolic phase image, and the area of the lumen was used as the region of interest for the flow and velocity quantification program (S11 Fig), yielding values that represent average volumetric flow rate and peak systolic velocity within the vessel [23]. The same magnetic resonance angiography scans were submitted to an imaging clinical research organization (Bioclinica) for archiving and for performing an additional exploratory analysis to compare the performance of another software platform that utilizes a fully automated image analysis algorithm. The RBF values and change from baseline data were highly correlated for both the serelaxin and terlipressin arms in comparison to the semi-automated approach. The use of an automated approach reduces reader bias and provides a platform to scale to evaluation of multicenter imaging data.

#### Biomarkers

All biomarker samples from the same patient were analyzed in the same batch by laboratory personnel at the central laboratory (Quintiles, UK) using commercially available assay kits. Ethylenediaminetetraacetic acid (EDTA) plasma nitrate levels were measured using the R&D Systems Parameter Total Nitric Oxide and Nitrate/Nitrite Assay. EDTA plasma endothelin-1 levels were measured using the Biomedica Endothelin ELISA. Lithium heparin plasma MMP-9 levels were measured using the R&D Systems Quantikine ELISA Human MMP-9 Immunoassay.

Blood samples from serelaxin-treated patients were collected for pharmacokinetics (relaxin-2 ELISA; R&D Systems) and immunogenicity analysis (anti-serelaxin antibody detection in a validated four-tiered assay approach) by Laboratory of the Government Chemist.

#### Safety assessments

Safety assessments consisted of collecting all data on AEs, SAEs, deaths, AE severity and relationship to study drug, and pregnancies. Monitoring of hematology and clinical chemistry were performed at a central laboratory, and assessments of vital signs and physical condition were performed regularly. ECGs were done locally at screening, and centrally read ECGs were done at four time points at visit 2 and sent electronically to a designated clinical research organization (ERT).

### Statistical analysis

Primary data from preclinical experiments are expressed as mean ± standard error of the mean (SEM), mean differences between groups, and 95% CIs. Differences among groups were assessed with one-way analysis of variance (ANOVA), two-way ANOVA, or repeated measures ANOVA with a Bonferroni correction, and between pairs with an unpaired two-tailed Student’s *t*-test. CRCs from myography were fitted to a sigmoidal curve using nonlinear regression. For the clinical trial, summary statistics for continuous variables were calculated (*n*, mean, standard deviation [SD], median, quartiles, minimum, maximum, geometric mean, and 95% CI on the geometric mean). Summary statistics for the baseline, post-baseline, and change from baseline measurements of blood flow for patients in the serelaxin group are presented for each time point. CIs were also constructed on the change from baseline parameters and were calculated for both the arithmetic and geometric means. A paired two-tailed Student’s *t*-test was used to compare baseline and post-baseline parameters. Data for the terlipressin group were analyzed in a similar manner as for the serelaxin group. GraphPad Prism version 5.03 (GraphPad Software) was used to perform statistical calculations. *p <* 0.05 was considered statistically significant.

## Results

### Rat models of cirrhosis and portal hypertension exhibited renal vasoconstriction and renal functional impairment

**Fig 1:**
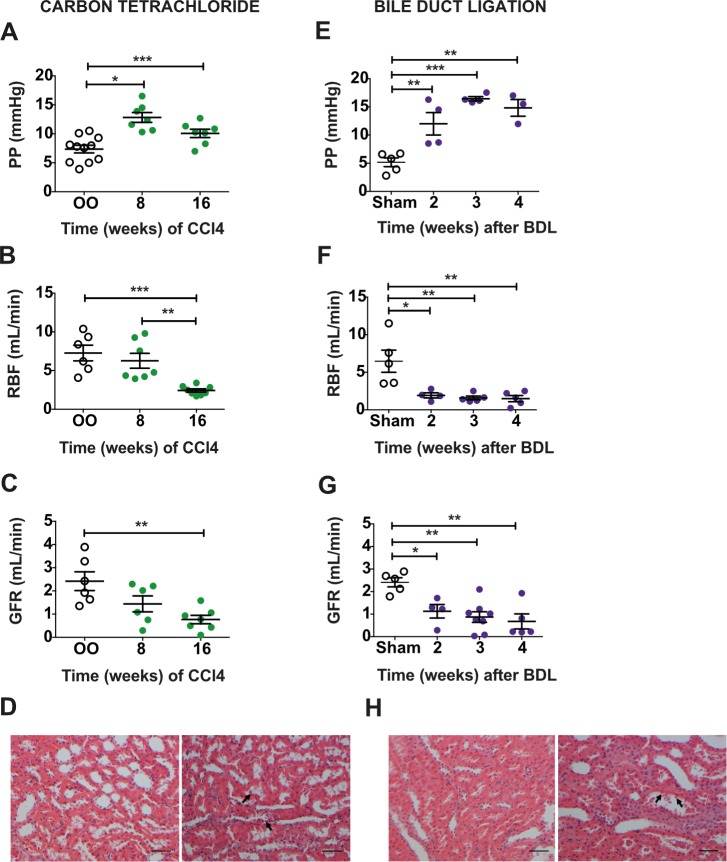
Rat models of advanced cirrhosis, portal hypertension, and renal dysfunction. Portal pressure (PP; A), renal blood flow (RBF; B), and glomerular filtration rate (GFR; C) in 16-wk CCl_4_ and olive oil (OO) control rats (*n =* 6–11). Representative H&E-stained kidney (scale bar 50 μm) showing minor tubular epithelial cell vacuolation (arrows) without significant necrosis after 16 wk of CCl_4_ (D). PP (E), RBF (F), and GFR (G) in bile duct ligation (BDL) and sham-operated (sham) control rats (*n =* 4–8). Representative H&E-stained kidney (scale bar 50 μm) showing necrotic cells within the tubule lumen and loss of the normal circumferential epithelial cell population (arrows) 4 wk after BDL (H). Data presented as mean ± standard error of the mean, analyzed by one-way ANOVA with post hoc Bonferroni correction (*p < 0.05; **p < 0.01; ***p < 0.001).

CCl_4_-treated rats developed cirrhosis and PHT (Figs 1A and S1A) but no jaundice or significant ascites. After 16 wk, there was a marked reduction in RBF (CCl_4_ 2.4 ± 0.2 ml/min versus OO 7.3 ± 1.0 ml/min; mean difference *d* = −4.84, 95% CI −7.69, −1.99; *p* < 0.001) and GFR (CCl_4_ 0.8 ± 0.2 ml/min versus OO 2.4 ± 0.4 ml/min; *d =* −1.65, 95% CI −2.82 −0.48; *p =* 0.005) (Fig 1B and 1C). Urinary sodium levels showed a nonsignificant reduction in 16-wk CCl_4_ rats (CCl_4_ 34 mmol/l versus OO 55 mmol/l; *p =* 0.22), consistent with the sodium-retaining phenotype associated with this model [24]. Despite significant renal functional impairment, kidney histology in CCl_4_-treated animals showed only minor tubular injury (Fig 1D).

In contrast, following BDL, rats were jaundiced after 2 wk, and all had moderate to severe ascites after 4 wk. Liver histology showed florid inflammation, collagen accumulation, and architectural distortion (S1F Fig). The PP increased progressively after BDL (Fig 1E), whereas there was a substantial decrease in RBF (4-wk BDL 1.5 ± 0.4 ml/min versus sham 6.5 ± 1.5 ml/min; *d =* −4.97, 95% CI −8.48, −1.47; *p =* 0.0037) (Fig 1F) and GFR (4-wk BDL 0.7 ± 0.3 ml/min versus sham 2.4 ± 0.2 ml/min; *d =* −1.74, 95% CI −2.92, −0.55; *p =* 0.0023) (Fig 1G). Urinary sodium was variable in BDL rats, though 4-wk BDL levels were in line with published data [21] (4-wk BDL 46 mmol/l versus sham 58 mmol/l; *p =* 0.73). Kidney histology in BDL animals showed features of ATN (Fig 1H).

### Impaired renal arterial vascular responses and the role of nitric oxide

**Fig 2:**
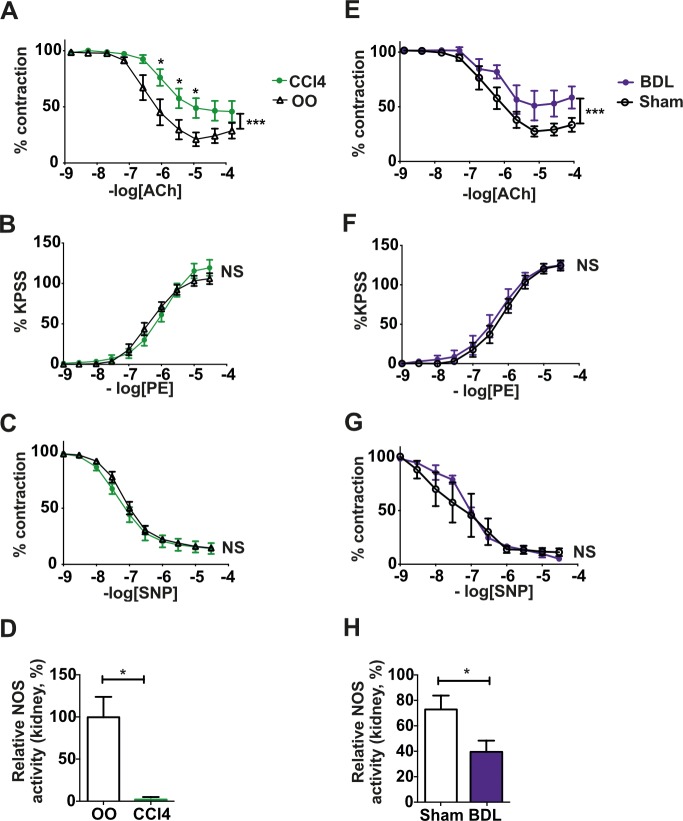
Vascular responses in isolated extrarenal renal arteries from cirrhotic rats. Concentration–response curves to acetylcholine (ACh; A and E), phenylephrine (PE; B and F), and sodium nitroprusside (SNP; C and G) in isolated extrarenal renal arteries from 16-wk CCl_4_ and 4-wk bile duct ligation (BDL) rats (*n =* 8–12). Data presented as mean ± standard error of the mean (SEM), relative to preconstricted value, analyzed by two-way ANOVA with post hoc Bonferroni correction (*p < 0.05; ***p < 0.001; NS, not significant). Nitric oxide synthase (NOS) activity in whole kidney extracts from 16-wk CCl_4_ (D) and 4-wk BDL (H) rats (*n =* 6–7). Data presented as mean ± SEM, analyzed by unpaired *t*-test (*p < 0.05). OO, olive oil.

Extrarenal renal arteries harvested from 16-wk CCl_4_ and 4-wk BDL rats showed a markedly attenuated response to the endothelium-dependent vasodilator ACh (Fig 2A and 2E). The −logIC_50_ (the −log of the concentration of ACh needed to reduce contraction by 50%) was lower in vessels from cirrhotic rats compared to controls (CCl_4_ 6.42 ± 0.14 versus OO 6.88 ± 0.11, *p <* 0.001; BDL 6.29 ± 0.12 versus sham 6.52 ± 0.05, *p <* 0.001), caused by a rightward shift in the CRC. The Emax to ACh (percent of maximal dilatation achieved) was lower in vessels from cirrhotic rats compared to controls (CCl_4_ 47.99% ± 8.57% versus OO 78.61% ± 5.17%, *p =* 0.0056; BDL 51.33% ± 11.76% versus sham 72.84% ± 5.86%, *p =* 0.13). Significant impairment of endothelium-dependent vasodilation was also observed at the level of the smaller (segmental and interlobar) intrarenal arteries in both cirrhosis models (S2A–S2D Fig). NOS was shown to be critical to endothelium-dependent renal arterial vasodilation in both normal and cirrhotic rats (S2E and S2F Fig). There were no differences in response to the vasoconstrictor phenylephrine or endothelium-independent vasodilator SNP in either model (Fig 2B, 2C, 2F and 2G).

### Nitric oxide synthase activity was reduced in kidneys from cirrhotic rats

There was a substantial reduction in renal NOS activity (Fig 2D and 2H) and phosphorylated eNOS (p-eNOS) protein (S3A and S3B Fig) in cirrhotic rats. Additionally, there was increased transcription in kidney of genes involved in the regulation of vascular tone (endothelin receptor A [*Ednra*] and B [*Ednrb*] and arginine vasopressin receptor 1a [*Agtr1a*]) and negative regulators of NOS activity (arginase-II [*Arg2*] and caveolin-I [*Cav1*]) (S3C and S3D Fig). Increased arginase-II and caveolin-I protein was also shown (S3E–S3H Fig).

### *RXFP1* expression was upregulated in kidney and localized to the renal vasculature in rat cirrhosis models

**Fig 3:**
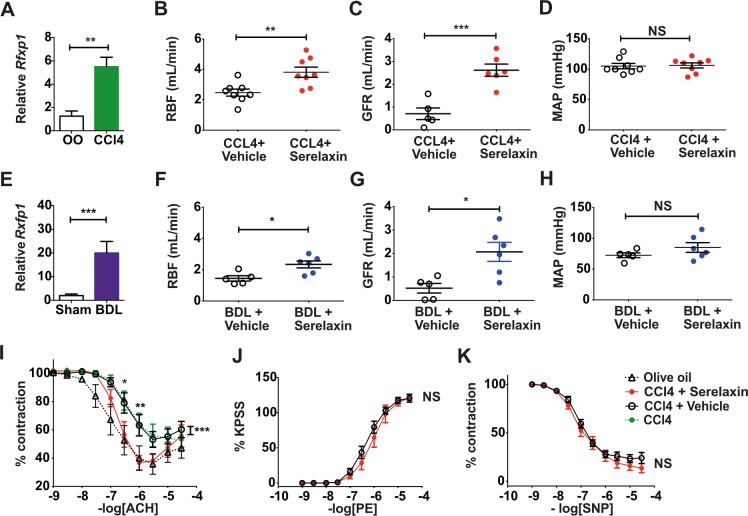
Effect of 72-h serelaxin infusion on renal perfusion and renovascular responses in cirrhotic rats. Relative *Rxfp1* transcripts (normalized to 18S rRNA) in whole kidney extracts from 16-wk CCl_4_ (A) and 4-wk bile duct ligation (BDL) (E) rats (*n =* 3–6). Renal blood flow (RBF; B and F), mean arterial pressure (MAP; D and H), and glomerular filtration rate (GFR; C and G) in CCl_4_ and BDL rats after 72-h s.c. serelaxin or vehicle (*n =* 5–8). Data presented as mean ± standard error of the mean (SEM), analyzed by unpaired *t*-test (*p < 0.05; **p < 0.01; ***p < 0.001; NS, not significant). Concentration–response curves to acetylcholine (ACh; I), phenylephrine (PE; J), and sodium nitroprusside (SNP; K) in the presence of serelaxin or vehicle in 16-wk CCl_4_ rats (*n =* 5–8). Data presented as mean ± SEM, analyzed by two-way ANOVA with post hoc Bonferroni correction (*p < 0.05; **p < 0.01; ***p < 0.001). OO, olive oil.

*Rxfp1* transcript levels were markedly increased in 16-wk CCl_4_ and 4-wk BDL rat kidneys compared to controls (CCl_4_: *d =* 4.2, 95% CI 1.77, 6.71, *p =* 0.0042; BDL: *d =* 18, 95% CI 11.15, 24.84, *p* < 0.001) (Fig 3A and 3E). Relative RXFP1 protein levels were also increased in 16-wk CCl_4_ and, to a lesser extent, 4-wk BDL rat kidneys (S4B and S4D Fig). RXFP1 staining localized to renal vascular endothelium and smooth muscle, as well as perivascular adventitial cells (S4E Fig). Kidney biopsies are not routinely performed for the clinical diagnosis of HRS, but a similar staining pattern for RXFP1 was observed in normal human kidney tissue (S4F Fig).

### Sustained serelaxin infusion increased renal perfusion and reversed renal dysfunction in advanced CCl_4_ cirrhotic rats

Mean serum serelaxin levels were 10.2 ± 1.3 ng/ml in 16-wk CCl_4_-treated rats after 72-h serelaxin infusion (4 μg/h s.c.), but serelaxin was undetectable following vehicle infusion. Serelaxin decreased RVR compared to vehicle (29.3 ± 2.8 mm Hg/ml/min versus 46.46 ± 6.9 mm Hg/ml/min; *d =* −17.18, 95% CI −33.07, −1.29; *p =* 0.04) (S5A Fig) and increased RBF by 54% (3.8 ± 0.3 ml/min versus 2.5 ± 0.2 ml/min; *d =* 1.34, 95% CI 0.47, 2.22; *p =* 0.005) (Fig 3B). Furthermore, serelaxin increased GFR by 371% compared to vehicle (2.6 ± 0.3 ml/min versus 0.7 ± 0.3 ml/min; *d =* 1.91, 95% CI 1.06, 2.76; *p* < 0.001) (Fig 3C), completely restoring GFR to that of control rats. The filtration fraction (FF; GFR/RBF) increased from 0.32 ± 0.09 to 0.79 ± 0.12 (*d =* 0.47, 95% CI 0.12, 0.83; *p =* 0.015) following serelaxin treatment (S5C Fig). However, there was no difference in MAP (serelaxin 106 ± 4 mm Hg versus vehicle 105 ± 4 mm Hg; *d =* 1.13, 95% CI −12.06, 14.31; *p =* 0.86) (Fig 3D) or serum total nitrite levels (serelaxin 18.6 ± 1.7 μmol/l versus vehicle 16.5 ± 2.8 μmol/l; *d =* 2.4, 95% CI −4.99, 9.8; *p =* 0.48) (S6A Fig). The effects of serelaxin on RBF and GFR were not associated with a change in the degree of liver injury or fibrosis (S6C and S6E Fig).

### Sustained serelaxin treatment increased renal perfusion and reversed renal dysfunction in decompensated bile-duct-ligated cirrhotic rats

To determine whether the effects of serelaxin on RBF and GFR were dependent on model or disease stage, we repeated all our analyses in BDL rats with decompensated cirrhosis. Mean serum serelaxin levels were 10.6 ± 1.8 ng/ml in 3-wk BDL rats after 72-h serelaxin infusion (4 μg/h s.c.), but serelaxin was undetectable following vehicle infusion. Serelaxin decreased RVR compared to vehicle (35.9 ± 2.4 mm Hg/ml/min versus 53.1 ± 7.4 mm Hg/ml/min; *d =* −17.2, 95% CI −32.34, −2.08; *p =* 0.03) (S5B Fig) and increased RBF by 53% (2.3 ± 0.2 ml/min versus 1.5 ± 0.2 ml/min; *d =* 0.84, 95% CI 0.23, 1.46; *p =* 0.012) (Fig 3F). Moreover, serelaxin increased GFR by 350% compared to vehicle (2.1 ± 0.5 ml/min versus 0.6 ± 0.2 ml/min; *d =* 1.55, 95% CI 0.45, 2.65; *p =* 0.011) (Fig 3G), representing an improvement to 86% of sham control levels. The FF increased from 0.25 ± 0.14 to 0.84 ± 0.11 (*d =* 0.58, 95% CI 0.18, 0.98; *p =* 0.009) following serelaxin treatment (S5D Fig). However, there were no differences in MAP (serelaxin 81 ± 8 mm Hg versus vehicle 73 ± 4 mm Hg; *d =* 8.72, 95% CI −13.08, 30.51 *p =* 0.39) (Fig 3H) or serum total nitrite level (serelaxin 30.7 ± 7.2 μmol/l versus vehicle 30.8 ± 4.2 μmol/l; *d =* 2.51, 95% CI −15.6, 20.6; *p =* 0.76) (S6B Fig), and the effects of serelaxin were independent of biliary injury and liver fibrosis (S6D and S6F Fig).

### Sustained serelaxin treatment restored endothelium-dependent vasodilation in isolated renal arteries from CCl_4_ cirrhotic rats

Serelaxin treatment (4 μg/h s.c. for 72 h) restored normal endothelium-dependent vasodilation responses in isolated extrarenal arteries, but no such effect was observed following vehicle infusion (Fig 3I). Serelaxin had no effect on vasoconstriction or endothelium-independent vasodilation in these arteries (Fig 3J and 3K).

### Serelaxin induced renal arterial vasodilation in cirrhotic rats through activation of the AKT/eNOS/NO signaling pathway

**Fig 4:**
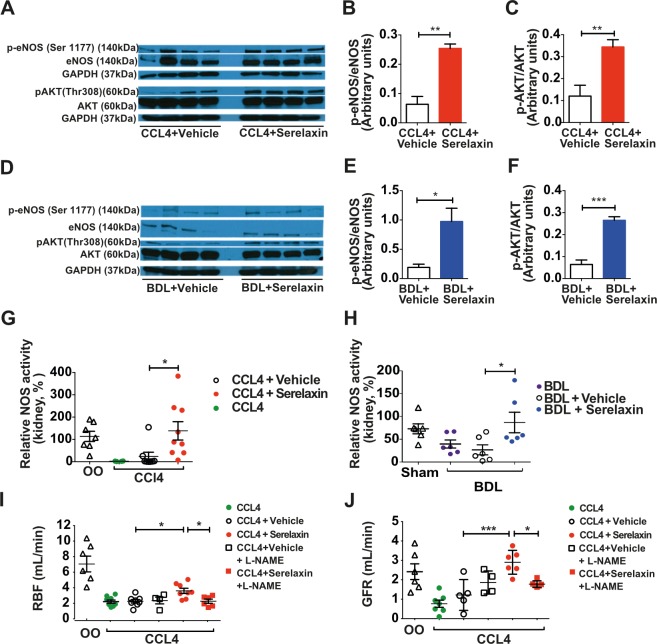
Effect of sustained serelaxin infusion on AKT/eNOS/NO signaling in cirrhotic rat kidney. Quantification of p-eNOS/eNOS and p-AKT/AKT in whole kidney extracts from 72-h serelaxin- or vehicle-treated 16-wk CCl_4_ and 4-wk bile duct ligation (BDL) rats (*n =* 4) (A–F). Data presented as mean ± standard error of the mean (SEM), analyzed by unpaired *t*-test (*p < 0.05; **p < 0.01; ***p < 0.001). NOS activity in whole kidney extracts from CCl_4_ (G) and BDL (H) rats treated with serelaxin or vehicle (*n =* 6–8). Data presented as mean ± SEM, analyzed by unpaired *t*-test (*p < 0.05; **p < 0.01). Renal blood flow (RBF; I) and glomerular filtration rate (GFR; J) in 16-wk CCl_4_ rats co-treated with L-N^G^-nitroarginine methyl ester (L-NAME) (*n =* 4–8). Data presented as mean ± SEM, analyzed by one-way ANOVA with post hoc Bonferroni correction (*p < 0.05; **p < 0.01; ***p < 0.001). OO, olive oil.

Serelaxin treatment (4 μg/h s.c. for 72 h) increased p-eNOS and p-AKT protein in whole kidney in both rat cirrhosis models (Fig 4A–4F) and augmented NOS activity to levels approximating those of controls (CCl_4_: serelaxin 122% ± 31% of control kidney NOS activity versus vehicle 5% ± 3%; *d =* 114, 95% CI 6.45, 223, *p =* 0.035; BDL: serelaxin 86% ± 22% versus vehicle 26% ± 11%; *d =* 60, 95% CI 4.2, 115, *p =* 0.032) (Fig 4G and 4H). To confirm NOS requirement, subgroups of CCl_4_ cirrhotic rats were co-treated with L-NAME (250 mg/l per os). L-NAME inhibited eNOS expression and NOS activity in kidney (S7A–S7C Fig) and increased MAP (S7D Fig), but abrogated the effects of serelaxin on RBF and GFR (Fig 4I and 4J).

### Renal blood flow and oxygenation in rats with advanced cirrhosis was augmented by a single intravenous bolus of serelaxin

**Fig 5:**
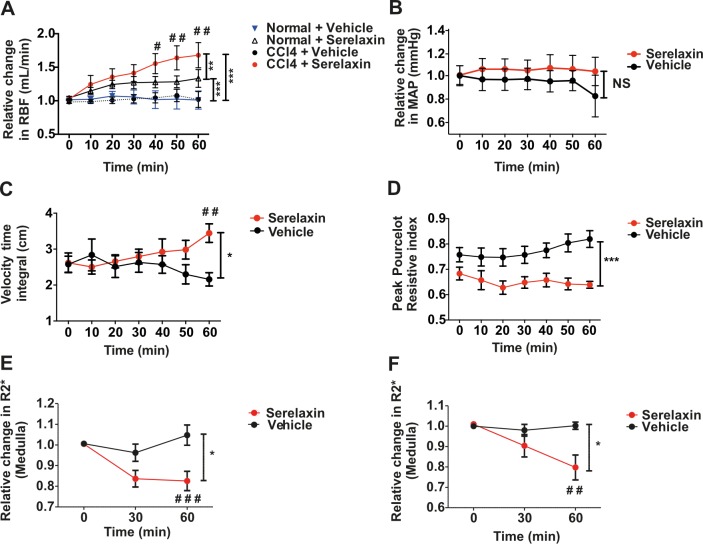
Effect of acute serelaxin treatment on renal blood flow and tissue oxygenation in CCl_4_ cirrhotic rats. Renal blood flow (RBF, A) and mean arterial pressure (MAP, B) responses to acute i.v. serelaxin (4 μg) or vehicle in 16-wk CCl_4_ rats (*n =* 5–7). Measurement of velocity time integral (C) and renal resistive index (D) following acute i.v. serelaxin (4 μg) or vehicle (*n =* 6–8). Deoxygenated hemoglobin levels (R2*) in renal medulla in 8-wk (E) and 16-wk (F) CCl_4_ rats at baseline, 30 min, and 60 min following acute i.v. serelaxin (4 μg) or vehicle (*n =* 5–8). Data presented as mean ± standard error of the mean, analyzed by two-way ANOVA (*p < 0.05; **p < 0.01; ***p < 0.001; NS, not significant) with post hoc Bonferroni correction to compare individual CCl_4_ time points with respective vehicle controls (^#^*p <* 0.05; ^##^*p <* 0.01; ^###^*p <* 0.001).

The mean serum serelaxin level at 60 min after serelaxin i.v. injection (4 μg in 200 μl) was 6.0 ± 0.3 ng/ml, whereas serelaxin was undetectable after vehicle injection (200 μl). In CCl_4_ cirrhotic rats, a single i.v. serelaxin bolus increased RBF by 50% from baseline after 60 min (*d =* 0.66, 95% CI 0.26, 1.05; *p =* 0.016) (Fig 5A). Age- and sex-matched healthy control rats showed a smaller (30%) increase from baseline in RBF at 60 min following serelaxin (*d =* 0.29, 95% CI 0.05, 0.53; *p =* 0.019) (Fig 5A). Serelaxin induced a modest increase in resting HR (S8A and S8B Fig) but had no effect on MAP in cirrhotic rats (Fig 5B).

The effects of serelaxin on RBF and CO were also measured noninvasively by ultrasound. Over a 60-min observation period, treatment of CCl_4_ cirrhotic rats with serelaxin (4 μg in 200 μl) increased the renal arterial velocity time integral relative to vehicle control (*d =* 1.29, 95% CI 0.25, 2.32; *p =* 0.007) (Fig 5C), and there was a significant effect of serelaxin treatment on renal resistive index (F[1,92] = 11.66, *p =* 0.001) (Fig 5D). There was no change in CO at 60 min following either serelaxin or vehicle (S8C Fig).

Additionally, serelaxin (4 μg in 200 μl) reduced levels of R2* (indicative of lower levels of deoxygenated hemoglobin) in the renal medulla and cortex of 8-wk (early cirrhosis) and 16-wk (established cirrhosis) CCl_4_ rats after 60 min measured using BOLD-MRI (Figs 5E, 5F and S8D), whereas vehicle (200 μl) had no effect.

As NO is pivotal to the mechanism of vasodilation proposed for serelaxin [25], we also investigated the acute hemodynamic response to the NO-releasing drug SNP using doses previously shown to increase hind-limb blood flow in rats [26]. In contrast to the selective effect of i.v. serelaxin on RBF (and PP [17]), with preservation of MAP, SNP caused a dose-dependent decrease in MAP and failed to increase renal perfusion (S9 Fig).

### Serelaxin exhibited systemic anti-inflammatory effects and downregulated vasoconstrictor genes in kidney

Tumor necrosis factor α (TNFα) is a major pro-inflammatory cytokine that contributes to endothelial dysfunction through dysregulation and uncoupling of eNOS [27]. Baseline serum TNFα concentrations were significantly increased in both cirrhosis models (OO 20.5 ± 0.5 pg/ml versus 16-wk CCl_4_ 1,102 ± 270 pg/ml, *d =* 1,081, 95% CI 470, 1,693, *p* < 0.001; sham 727 ± 219 pg/ml versus BDL 4,006 ± 247 pg/ml, *d =* 3,279, 95% CI 1,417, 5,141, *p* < 0.001). Serelaxin infusion for 72 h (4 μg/h s.c.) reduced serum TNFα levels (CCl_4_: serelaxin 94.7 ± 61 pg/ml versus vehicle 817.3 ± 151 pg/ml, *d =* −722, 95% CI −1,400, −45.2, *p =* 0.033; BDL: serelaxin 1,516 ± 549 pg/ml versus 3,933 ± 391 pg/ml, *d =* −2,417, 95% CI −4,005, −828, *p =* 0.0014) (S10A and S10B Fig). Serelaxin also decreased transcripts for *Ednra*, urotensin-II receptor (*Uts2r*), and urotensin-II (*Uts2*) in the kidneys of cirrhotic rats (S10C Fig).

### Results of an exploratory phase 2 study of serelaxin in patients with compensated cirrhosis and portal hypertension

**Fig 6:**
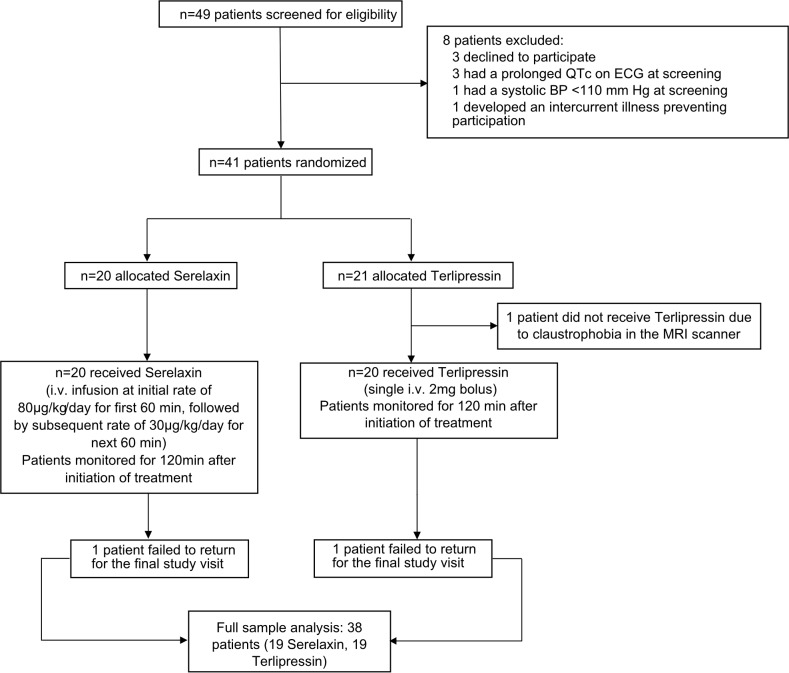
Flow chart of participants in the study.

Based upon the positive results of serelaxin treatment in experimental models, we proceeded to a human study that consisted of two parts: Part A and Part B. Here we present the data from Part A. A total of 41 male and female participants with compensated alcohol-related cirrhosis and PHT were randomized, and 40 went on to receive serelaxin or terlipressin treatment in a 1:1 ratio (Fig 6). Of these, 38 participants (19 in each treatment group) completed the study. Two participants (one from each group) did not return for the end of study visit but were confirmed to be alive at 90 d after their scheduled end of study visit. The main demographic and clinical characteristics of the study participants are shown in Table 1. Baseline data were generally comparable between the two treatment arms. A summary of the hemodynamic data from the study is presented in S1 Table.

**Table 1:** Baseline demographic and clinical characteristics of study participants at screening.

Characteristic	Serelaxin (*n =* 20)	Terlipressin (*n =* 20)
Sex, *n* (percent) male/female	16 (80%)/4 (20%)	16 (80%)/4 (20%)
Age (years)*	60 (57, 64)	55 (51, 58)
Body mass index (kg/m^2^)*	30 (28, 32)	28 (25, 31)
Alcohol use, *n* (percent) A/L/O^†^	13 (65%)/7 (35%)/0	10 (50%)/4 (20%)/6 (30%)
Albumin (g/l)*	40 (37, 43)	41 (39, 42)
Bilirubin (μmol/l)*	12 (8, 18)	19 (12, 29)
INR*	1.2 (1.1, 1.3)	1.2 (1.1, 1.3)
Creatinine (μmol/l)*	73 (66, 81)	66 (59, 73)
eGFR (ml/min/1.73 m^2^)*	90 (78, 103)	104 (89, 120)
Platelet count (× 10^−3^)*	77 (42, 143)	95 (68, 134)
Mean arterial pressure (mm Hg)*	108 (101, 115)	105 (98, 112)
Heart rate (beats/min)*	71 (67, 75)	65 (61, 70)
Child-Pugh score*	5 (5, 6)	5 (5, 6)
MELD score*	9 (8, 10)	10 (8, 11)
Previous variceal bleed, *n* (percent)	7 (35%)	3 (15%)
Splenomegaly, *n* (percent)	13 (65%)	15 (75%)

*Data expressed as mean (95% CI), except mean arterial pressure and heart rate, which are expressed as geometric mean ± 95% CI.

^†^A = abstinent, L = less than one drink per day, O = one to two drinks per day.

eGFR, estimated glomerular filtration rate; INR, international normalized ratio; MELD, Model for End-Stage Liver Disease.

#### Dynamic blood flow measurements by PC-MRA

**Fig 7:**
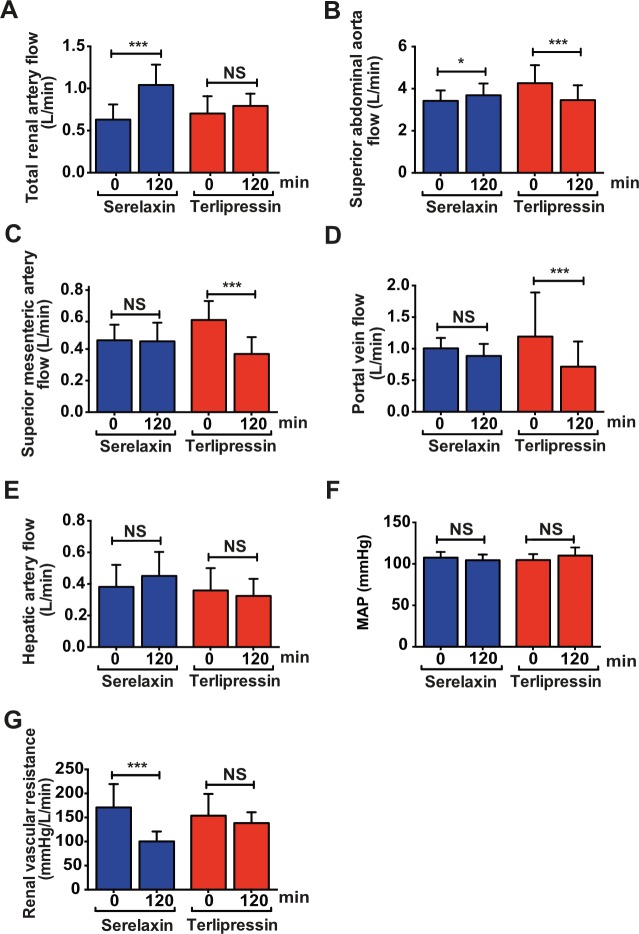
Differential effects of serelaxin and terlipressin treatment on splanchnic and extra-splanchnic hemodynamics. Blood flow changes in total renal artery (left + right renal artery; A), superior abdominal aorta (B), superior mesenteric artery (C), portal vein (D), and hepatic artery (E); mean arterial pressure (MAP; F); and renal vascular resistance (G) following serelaxin infusion (60 min at 80 μg/kg/d then 60 min at 30 μg/kg/d) or terlipressin (2-mg i.v. bolus). Data presented as geometric mean ± 95% CI, analyzed by paired *t*-tests for within-group comparison of baseline and post-treatment flow measurements (*n =* 20; *p < 0.05; ***p < 0.001; NS, not significant).

The primary outcome of the study was the change in total renal arterial blood flow measured by PC-MRA after 120 min of serelaxin infusion. Serelaxin infusion caused a substantial increase (+65%, 95% CI 40%, 95%) from baseline in total renal arterial blood flow (0.63 l/min, 95% CI 0.49, 0.81, versus 1.04 l/min, 95% CI 0.85, 1.28; *p <* 0.001), associated with a decrease in RVR (from 171 mm Hg/l/min, 95% CI 133, 219, to 100 mm Hg/l/min, 95% CI 83, 121; *p* < 0.001) (Fig 7A and 7G). There were only modest effects of serelaxin on secondary outcomes, including an increase in blood flow from baseline in the superior abdominal aorta (+8%, 95% CI 2%, 14%; *p =* 0.017) and nonsignificant changes in hepatic artery flow (+18%, 95% CI −3%, 44%; *p =* 0.11) and portal vein flow (−12%, 95% CI −22%, 0%; *p =* 0.11) (Fig 7; S1 Table). Notably, there was no change in blood flow from baseline in the SMA (Fig 7C) or in total liver blood flow (S1 Table).

Administration of a single 2-mg i.v. bolus of terlipressin caused no significant change from baseline in RVR or total renal arterial blood flow after 120 min (+14%, 95% CI −3%, 33%; *p =* 0.25). However, there was a substantial decrease in SMA flow (−37%, 95% CI −45%, −28%; *p <* 0.001), portal vein flow (−40%, 95% CI −57%, −16%; *p =* 0.01), azygos blood flow (−30%, 95% CI −41%, −18%; *p <* 0.001), and total liver blood flow (−35%, 95% CI −51%, −13%; *p =* 0.015) (Fig 7; S1 Table).

Although this study was not powered for comparison between serelaxin and terlipressin, the change from baseline blood flow in several vessels was significantly different between the treatment arms (S2 Table). Notably, the increase in total renal arterial blood flow was much greater following serelaxin compared with terlipressin (*d =* 0.35 ± 0.10, 95% CI 0.15, 0.54; *p =* 0.001).

#### Safety and tolerability assessments

Overall, serelaxin was well tolerated. A total of three patients (15%) in the serelaxin group and 11 patients (55%) in the terlipressin group developed at least one AE (Table 2). AEs associated with terlipressin (mainly gastrointestinal) were consistent with the product label. There were no deaths reported during treatment or the protocol-specified 4-wk follow-up period. No meaningful changes in liver function tests were associated with serelaxin treatment. There was no significant change in MAP from baseline following 120 min of serelaxin infusion (108 mm Hg, 95% CI 101, 115, versus 105 mm Hg, 95% CI 98, 111; *p =* 0.5002; Fig 7F; S1 Table), and no AEs of hypotension or any discontinuations due to a blood pressure decrease. Electrocardiographic data were evaluated by an independent vendor (ERT) and demonstrated that there was no relevant effect of serelaxin on cardiac repolarization or other electrocardiographic effects. One participant in each group experienced a treatment-emergent SAE during the study (serelaxin: vomiting and ascites; terlipressin: alcohol poisoning, fall, and noncardiac chest pain). In both cases, alcohol intoxication provided an explanation for these SAEs, and a certain causal relationship could not be established.

**Table 2:** Overview of safety data.

Adverse event	Serelaxin (*n =* 20)	Terlipressin (*n =* 20)
**Any AE**	3 (15%)	11 (55%)
**Suspected treatment-related AE**	1 (5%)	10 (50%)
**Any SAE**	1 (5%)	1 (5%)
**Suspected treatment-related SAE**	0	0
**Death up to 30 d**	0	0
**Dose interruption**	0	0
**Treatment withdrawal**	0	0
**AE regardless of study drug relationship**		
Abdominal pain	0	4 (20%)
Alcohol poisoning	0	1 (5%)
Ascites	1 (5%)	0
Cellulitis	1 (5%)	0
Diarrhea	0	6 (30%)
Electrocardiogram QT prolonged	1 (5%)	1 (5%)
Fall	0	1 (5%)
Headache	0	1 (5%)
Hypertension	0	3 (15%)
Noncardiac chest pain	0	1 (5%)
Pruritus	1 (5%)	0
Syncope	0	1 (5%)
Vomiting	1 (5%)	1 (5%)

Data given as *n* (percent). Adverse events (AEs) and serious adverse events (SAEs) are shown, regardless of study drug relationship, by preferred term and treatment group.

QT, QT interval.

#### Pharmacokinetic, immunogenicity, and biomarker evaluations in serelaxin-treated patients

**Fig 8:**
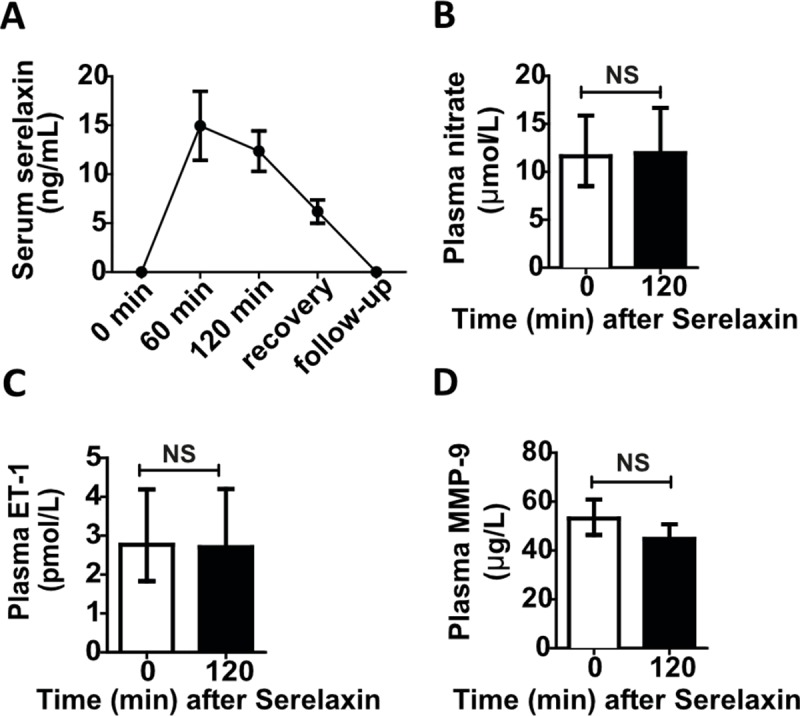
Effect of serelaxin infusion on pharmacokinetics and plasma biomarkers in patients with cirrhosis and portal hypertension. Serum serelaxin concentration measured by ELISA pre-dose (0 min), at 60 min and 120 min post-initiation of serelaxin infusion, in recovery period (~60 min after cessation of serelaxin), and at 4-wk follow-up visit (A). Data presented as mean ± standard deviation (*n =* 20). Plasma nitrate (B), endothelin-1 (ET-1) (C), and matrix metalloproteinase-9 (MMP-9) (D) measured by ELISA pre-dose (0 min) and at 120 min after initiation of serelaxin infusion. Data presented as geometric mean ± 95% CI (*n =* 20). NS, not significant.

No dose adjustments or interruptions of treatment were made in the study. Serum serelaxin concentration (Fig 8A) increased rapidly to 14.6 ng/ml (range 9.5, 23.2) following the initial 60-min fast infusion rate (80 μg/kg/d), then decreased to 12.2 ng/ml (range 8.1, 17.9) following the subsequent slower infusion rate (30 μg/kg/d), and declined rapidly to 6.1 ng/ml (range 4.4, 9.9) during the recovery phase (~60 min following cessation of serelaxin). Serelaxin was undetectable at week 4 of the study. No patients had detectable anti-serelaxin antibodies at baseline or at 4 wk post-treatment. There were no significant changes from baseline observed in levels of prespecified peripheral plasma biomarkers following serelaxin exposure (Fig 8B–8D; nitrate 11.6 pmol/l, 95% CI 8.5, 15.9, versus 12.0 pmol/l, 95% CI 8.6, 16.7; ET-1 2.8 pmol/l, 95% CI 1.8, 4.2, versus 2.7 pmol/l, 95% CI 1.7, 4.2; MMP-9 53.1 μg/l, 95% CI 46.3, 60.9, versus 44.8 μg/l, 95% CI 39.5, 50.7).

## Discussion

We have shown for the first time, to the best of our knowledge, that serelaxin attenuated renal vasoconstriction (RVR), improved RBF and endothelial dysfunction, and reversed impaired kidney function in experimental rat models of cirrhosis, PHT, and renal dysfunction (Fig 3). Crucially, we went on to demonstrate the translational relevance of these findings by showing in patients with cirrhosis and PHT that serelaxin reduced RVR and caused a substantial increase in RBF (Fig 7).

In two pathologically distinct rat models, advancement of cirrhosis was associated with a progressive reduction in RBF and GFR (Fig 1). The beneficial effects of serelaxin on RBF and GFR were dependent on an increase in renal NO signaling (as the effect was abrogated by co-treatment with L-NAME; Fig 4) and were associated with downregulation of vasoconstrictor receptor expression in cirrhotic rat kidney (S10 Fig). In contrast, administration of a nonselective nitrovasodilator induced hypotension and reduced RBF (S9 Fig), consistent with previous observations in human cirrhosis [28].

Both acute (i.v. bolus; Fig 5) and sustained (72-h s.c. infusion; Fig 3) serelaxin treatment induced a significant increase in RBF in cirrhotic rats. However, the relative effect of serelaxin on GFR was even greater (Fig 3). In both models the GFR was restored to near-normal levels and the FF increased (S5 Fig), suggesting a differential effect on afferent and efferent renal arteriolar tone. This is a significant observation because an increase in RBF by other renal vasodilators, such as dopamine, is not necessarily accompanied by an increase in GFR [29].

Additionally, serelaxin may augment renal function by modifying other nonvasomotor factors, such as inflammation, that also contribute to renal dysfunction in cirrhosis [30]. Indeed, previous studies have demonstrated that serelaxin is a vasoprotective molecule under conditions of acute inflammation/oxidative stress (reviewed in [31]). In our preclinical cirrhosis models, serelaxin decreased the (elevated) serum levels of TNFα (S10 Fig), a pro-inflammatory cytokine heavily implicated in endothelial dysfunction. Moreover, co-incubation of TNFα-exposed aortic rings with serelaxin was shown to increase eNOS activity, attenuate arginase-II expression, and improve vasorelaxation [31]. Both large and small renal arteries isolated from CCl_4_ and BDL cirrhotic rats exhibited marked impairment in endothelium-dependent vasodilation (Fig 2), consistent with a study in human cirrhosis linking endothelial dysfunction with the development of renal vasoconstriction and renal failure [32]. However, 72-h serelaxin infusion restored renovascular function (Fig 3) and decreased RVR (S5 Fig). Taken together, our findings indicate that serelaxin, through a number of mechanisms, modulated renal arterial endothelial responsiveness in cirrhosis in favor of vasodilatation.

Notably, serelaxin treatment also decreased transcription of the vasoconstriction-related genes *Ednra* and *Uts2r* in cirrhotic rat kidneys (S10 Fig). Endothelin receptor A is a smooth muscle receptor that mediates increased vascular tone after stimulation by endothelin-I whereas urotensin-II is a potent vasoconstrictor of large conductive vessels but, paradoxically, relaxes mesenteric vessels. Circulating levels of urotensin-II are elevated in human cirrhosis, and a urotensin-II receptor antagonist reduced PP and increased RBF in BDL rats [33].

The levels of transcripts for the cognate receptor for human relaxin-2 (*Rxfp1*) were increased in whole kidney and renal artery extracts in cirrhotic rats (Fig 3). Furthermore, RXFP1 protein localized to renal vessels (endothelium and smooth muscle) and perivascular adventitial cells in both rat and human kidney (S4 Fig). Investigation of differential expression of RXFP1 and relative dilator sensitivity to serelaxin in the afferent and efferent renal arterioles (e.g., by videomicroscopy) is an interesting topic for future study. Moreover, the factors that regulate renal parenchymal and arterial expression of RXFP1 in animal models and human disease are unknown, although a recent study showed that α1- and β1-adrenoceptors regulated cardiac expression of RXFP1 in mice [34].

Encouraged by preclinical studies, we undertook an exploratory trial in patients with compensated cirrhosis and PHT. The hemodynamic changes induced by terlipressin were consistent with its known activity as a potent splanchnic and systemic vasoconstrictor and validated the use of PC-MRA in this population (Fig 7; S1 Table). Infusion of serelaxin for 120 min achieved similar steady-state serum concentrations to those observed in our 72-h rat cirrhosis models and in human heart failure following 48-h i.v. infusion [21]. Baseline RBF levels in our cirrhosis population were lower than that reported in a large series of healthy volunteers (0.838 ± 0.244 l/min), measured using PC-MRA [35]. Serelaxin treatment elicited a selective, highly significant, and clinically relevant increase in total RBF that was associated with a substantial reduction in RVR but only a small increase in superior aortic flow, as a surrogate of increased CO (Fig 7). Effects on key secondary outcomes included a nonsignificant decrease in portal venous flow (Fig 7), but, importantly, total hepatic blood flow was preserved (S1 Table)—in contrast to splanchnic vasoconstrictors such as terlipressin that further compromise hepatic perfusion in cirrhosis and may impair organ function. Additionally, there was no increase in SMA flow or azygos flow and, critically, no detrimental effects on blood pressure or increase in circulating NO levels (Fig 8). Interestingly, our demonstration of differential visceral blood flow responses in cirrhotic patients using MRI are consistent with the preclinical literature, which has revealed that serelaxin preferentially dilates constricted vessels and exerts quite heterogeneous responses across distinct regions of the vasculature [36,37].

There are limitations to our study that merit discussion. It should be acknowledged that there is no ideal animal model of renal dysfunction in cirrhosis (especially HRS), and therefore one must be cautious regarding the generalizability of preclinical observations. However, our findings in CCl_4_ and BDL rats corroborate clinical data showing that advancement of hepatic fibrosis is associated with progressive renal vasoconstriction and functional impairment—seen at its most extreme in HRS [10,38,39]. Moreover, BDL is considered a suitable model of human HRS [40] and was recently shown to share innate immune defects and clinical characteristics with acutely decompensated cirrhosis in patients [41]. Although we and others [42] observed features of ATN in rat BDL kidneys, emerging histological and biomarker studies indicate that tubular injuries are frequently present in cirrhotic patients with renal dysfunction [43,44]. The primary objectives of our clinical study were to define the hemodynamic effects and safety profile of serelaxin in patients with cirrhosis and PHT. Although this exploratory trial was (necessarily) open-label, we ensured that outcome assessors were blinded to treatment allocation to avoid potential bias. Additionally, MRA scans were reanalyzed by an independent clinical research organization using a fully automated approach to completely eliminate possible reader bias; automated and semi-automated data were highly correlated. It should also be noted that the study population consisted of patients with well-compensated cirrhosis and PHT, but without significant impairment of renal function. Further studies are now required in patients with more advanced cirrhosis and renal dysfunction to link the substantial increase in RBF we observed with serelaxin to improvements in renal function and patient outcomes. Recent clinical data provide encouragement in this regard. First, the pharmacokinetic and safety profiles of serelaxin were not affected in patients with mild, moderate, and severe hepatic impairment [45]. Second, serelaxin had a beneficial effect on renal biomarkers (creatinine and cystatin C) and survival in patients with acute heart failure [46].

In summary, our mechanistic findings in rat models and exploratory study in human cirrhosis suggest a potential therapeutic role for serelaxin in the treatment of renal dysfunction in cirrhosis and possibly other human conditions where RBF or endothelial function is compromised.

## Supporting information

S1 Appendix Graphical raw data. (XLSX)

S2 Appendix Blot raw data. (ZIP)

S3 Appendix Image raw data. (ZIP)

S1 Fig Characterization of rat cirrhosis models. (A) Representative rat liver stained with hematoxylin and eosin (H&E) and picrosirius red (PSR) (scale bar 200 μm) in 8- and 16-wk olive oil (OO)-treated and CCl_4_-treated rats (*n =* 6–12) showing necroinflammation (arrowheads) and accumulation of fibrillar collagen (arrows). (B) Collagen proportionate area (CPA, percent) calculated by morphometric analysis of PSR staining. (C) Alanine aminotransferase (ALT) (one-way ANOVA: F[2,19] = 3.78; *p =* 0.042) and (D) albumin levels quantified in serum (one-way ANOVA: F[2,17] = 3.8; *p =* 0.043). (E) Representative H&E- and PSR-stained rat liver (scale bar 200 μm) showing necroinflammation (arrowheads) and deposition of fibrillar collagen (arrows) after bile duct ligation (BDL) (*n =* 5–6). (F) CPA, (G) serum alkaline phosphatase (ALP), and (H) albumin levels. (I) Acute tubular necrosis (ATN) score in BDL rat kidney. Data presented as mean ± standard error of the mean, analyzed by one-way ANOVA with post hoc Bonferroni correction (*p < 0.05; **p < 0.01; ***p < 0.001; NS, not significant).
(PDF)

S2 Fig Renal vascular endothelial function in rat cirrhosis models. Concentration–response curves to acetylcholine (ACh; 10^−9^ to 10^−5^ M) in isolated segmental (A) and interlobar (B) renal arteries from 16-wk CCl_4_ and olive oil (OO) rats (*n =* 8–12) and in segmental (C) and interlobar (D) arteries from 4-wk bile duct ligation (BDL) and sham rats. Concentration–response curves to ACh (10^−9^ to 10^−5^ M) in extrarenal renal arteries from 16-wk OO (E) and CCl_4_ (F) rats co-treated with L-N^G^-nitroarginine methyl ester (L-NAME) (1 × 10^−4^ M), indomethacin (1 × 10^−5^ M), or apamin (1 × 10^−4^ apamin M) plus charybdotoxin (1 × 10^−5^ M). Data expressed as mean ± standard error of the mean, analyzed by two-way ANOVA with post hoc Bonferroni correction (*p < 0.05; *p < 0.01; ***p < 0.001).
(PDF)

S3 Fig Regulation of vasomotor factors in rat cirrhosis models. Quantification of relative p-eNOS/eNOS protein expression in whole kidney extracts from 16-wk CCl_4_ (A) and 4-wk bile duct ligation (BDL) (B) rat kidney (*n =* 4). Fold regulation of mRNA transcripts for vasoregulatory genes in kidney tissue from 16-wk CCl_4_ rats compared to olive oil (OO) rats (C) and BDL rats compared to sham rats (D) (*n =* 3–6). Genes with fold regulation >2 with *p <* 0.05 are presented (arginase-II, *Arg2*; caveolin-I, *Cav1*; endothelin receptor A, *Ednra*; endothelin receptor B, *Ednrb*). Quantification of arginase-II protein (E and F) and caveolin-I (G and H) relative to GAPDH in 16-wk CCl_4_ and 4-wk BDL rat kidney compared to controls. Data expressed as mean ± standard error of the mean, analyzed by unpaired two-tailed *t*-test (*p < 0.05; **p < 0.01; ***p < 0.001; NS, not significant).
(PDF)

S4 Fig Expression and distribution of RXFP1 in cirrhotic rat and normal human kidney. Relative *Rxfp1* transcript level (normalized to 18S rRNA) in renal artery extracts from 16-wk CCl_4_ (A; *p =* 0.059) and 4-wk bile duct ligation (BDL) (C; *p =* 0.067) rats (*n =* 3–6). Quantification of RXFP1 protein relative to GAPDH in whole kidney from 16-wk olive oil (OO) and CCl_4_ (B) and 4-wk sham and BDL (D) rats (*n =* 4). Data expressed as mean ± standard error of the mean, analyzed by unpaired two-tailed *t*-test (*p < 0.05). Representative immunofluorescence staining for RXFP1 (and isotype control) in 16-wk OO and CCl_4_ kidney, in 4-wk sham and BDL kidney (E), and in biopsy tissue from normal human kidney (F). DAPI nuclear counterstain (size bars 100 μm).
(PDF)

S5 Fig Effect of 72-h serelaxin infusion on renal vascular resistance and filtration fraction in cirrhotic rats. Renal vascular resistance (RVR; A and B) and filtration fraction (FF; C and D) in CCl_4_ and bile duct ligation (BDL) rats after 72-h subcutaneous serelaxin or vehicle (*n =* 5–8). Data presented as mean ± standard error of the mean, analyzed by unpaired *t*-test (*p < 0.05; **p < 0.01; ***p < 0.001; NS, not significant).
(PDF)

S6 Fig Effect of 72-h serelaxin infusion on serum and tissue markers in rat cirrhosis models. Serum total nitrite levels in 16-wk CCl_4_ (A) and 4-wk bile duct ligation (BDL) (B) rats after 72-h subcutaneous serelaxin or vehicle infusion. Serum alanine aminotransferase (ALT) levels in 16-wk CCl_4_ rats (C) and alkaline phosphatase (ALP) levels in 4-wk BDL (D) rats. Data expressed as mean ± standard error of the mean (SEM) (*n =* 6–8), analyzed by unpaired two-tailed *t*-test (NS, not significant). Collagen proportionate area (CPA, percent) measured by morphometric analysis of picrosirius-red-stained liver sections in 16-wk CCl_4_ (E) and 4-wk BDL (F) rats (*n =* 4). Data expressed as mean ± SEM, analyzed by unpaired two-tailed *t*-test.
(PDF)

S7 Fig Effect of L-N^G^-nitroarginine methyl ester administration on nitric oxide bioavailability and mean arterial pressure in serelaxin-treated CCl_4_ rats. Quantification of eNOS protein (relative to GAPDH loading control) in whole kidney extracts from subgroups of 16-wk CCl_4_ rats randomized to treatment with serelaxin ± L-N^G^-nitroarginine methyl ester (L-NAME) (A) or vehicle ± L-NAME (B) (*n =* 4). Data expressed as mean ± standard error of the mean (SEM), analyzed by unpaired two-tailed *t*-test (*p < 0.05; ***p < 0.001) (C). Nitric oxide synthase (NOS) activity in whole kidney extracts (*n =* 4–8). Mean arterial pressure (MAP) response (D) (*n =* 4–8). Data expressed as mean ± SEM, analyzed by one-way ANOVA with post hoc Bonferroni correction (*p < 0.05; **p < 0.01; ***p < 0.001; NS, not significant).
(PDF)

S8 Fig Hemodynamic effects of acute serelaxin treatment in CCl_4_ cirrhotic rats. Heart rate (HR; beats per min, bpm) measured in 16-wk CCl_4_ rats (*n =* 7) randomized to intravenous (i.v.) serelaxin (4 μg in 200 μl, A) or vehicle (200 μl, B) for 60 min by femoral artery catheter. Cardiac output by M-mode Doppler (C) (*n =* 6–9). HR data presented as individual rats at each time point; CO data plotted at baseline and 60 min for individual rats. HR data analyzed using one-way ANOVA and CO data by two-way ANOVA, both with post hoc Bonferroni correction (***p < 0.001; NS, not significant). Deoxygenated hemoglobin levels (R2*) in renal cortex measured by blood-oxygen-level-dependent MRI in 8-wk CCl_4_-treated rats (*n =* 7) at baseline and 30 min and 60 min following i.v. serelaxin (4 μg in 200 μl) or vehicle (200 μl) (D). Data expressed as mean ± standard error of the mean, analyzed by two-way ANOVA with post hoc Bonferroni correction (*p < 0.05).
(PDF)

S9 Fig Hemodynamic effects of a classical nitrovasodilator in CCl_4_ cirrhotic rats. Effect of acute intravenous boluses of sodium nitroprusside (SNP; dose range 0.09–9 μg) on mean arterial pressure (MAP; A) in 16-wk CCl_4_ rats at serial time points, and the percentage change in renal blood flow (RBF) from baseline (B) over a 10-min observation period (*n =* 6). Data expressed as mean ± standard error of the mean (SEM), analyzed by one-way ANOVA with post hoc Bonferroni correction. Dose–response effect of SNP at baseline compared to 10-min MAP (C) and RBF (D). Data points represent mean ± SEM, analyzed by paired two-tailed *t*-test (*p < 0.05).
(PDF)

S10 Fig Effect of 72-h serelaxin infusion on serum TNFα and renal vasoconstrictor gene expression. Serum TNFα levels measured by ELISA in 4-wk sham and bile duct ligation (BDL) (A) and 16-wk olive oil (OO) and CCl_4_ (B) rats treated for 72 h with subcutaneous serelaxin or vehicle (*n =* 5–8). Data presented as individual values with mean ± standard error of the mean, analyzed by one-way ANOVA with post hoc Bonferroni correction (*p < 0.05; *p < 0.01; ***p < 0.001). Fold regulation of mRNA transcripts for vasoregulatory genes in kidney tissue from 16-wk CCl_4_ rats compared to OO rats (*n =* 5) (C). Genes with a fold regulation of >2 and significance of *p <* 0.05 are presented.
(PDF)

S11 Fig Magnetic resonance angiography. (A) Position of hepatic artery flow measurement (taken from 3-D magnetic resonance reconstruction of arterial system). (B) Phase contrast velocity-encoded images acquired from selected vessels were analyzed for peak and average flow over the cardiac cycle.
(PDF)

S1 Table Summary of hemodynamic effects of serelaxin and terlipressin in patients with cirrhosis and portal hypertension. Total renal artery flow = left + right renal artery flow; total liver flow = hepatic artery + portal vein flow. Renal vascular resistance = mean arterial pressure/total renal arterial blood flow.
(DOCX)

S2 Table Comparative analysis of the change in blood flow from baseline in response to serelaxin and terlipressin treatment. An unpaired *t*-test was used to compare the difference in blood flow change from baseline induced by serelaxin and terlipressin in prespecified vessels. Mean differences, 95% CIs, and *p-*values are presented in the table.
(DOCX)

S1 Text Clinical trial protocol. (PDF)

S2 Text CONSORT checklist. (DOC)

S3 Text NC3Rs ARRIVE checklist. (PDF)

## Abbreviations

ACh: acetylcholine
AE: adverse event
AKI: acute kidney injury
ANOVA: analysis of variance
ATN: acute tubular necrosis
BDL: bile duct ligation
BOLD-MRI: blood-oxygen-level-dependent magnetic resonance imaging
CI: confidence interval
CO: cardiac output
CRC: concentration–response curve
FF: filtration fraction
GFR: glomerular filtration rate
HR: heart rate
HRS: hepatorenal syndrome
i.v: intravenous
L-NAME: L-N^G^-nitroarginine methyl ester
MAP: mean arterial pressure
MRI: magnetic resonance imaging
NO: nitric oxide
NOS: nitric oxide synthase
OO: olive oil
PC-MRA: phase contrast magnetic resonance angiography
PHT: portal hypertension
PP: portal pressure
PSS: physiological salt solution
RBF: renal blood flow
RVR: renal vascular resistance
s.c.: subcutaneous
SAE: serious adverse event
SD: standard deviation
SEM: standard error of the mean
SMA: superior mesenteric artery
SNP: sodium nitroprusside
