# Supplementary figures and images for: Serelaxin as a potential treatment for renal dysfunction in cirrhosis: Preclinical evaluation and results of a randomized phase 2 trial
Source: PLoS Med. 2017 Feb 28;14(2):e1002248. doi: 10.1371/journal.pmed.1002248 (PMC5330452; doi:10.1371/journal.pmed.1002248)

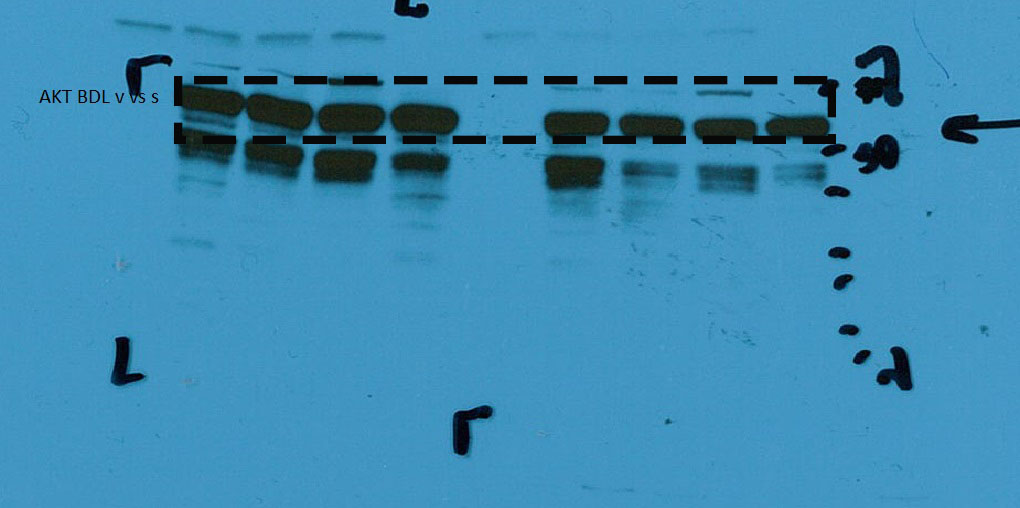

Supplement: S2 Appendix — (ZIP) [file pmed.1002248.s002.zip › AKT BDL veh vs serelaxin Fig4D.jpg]

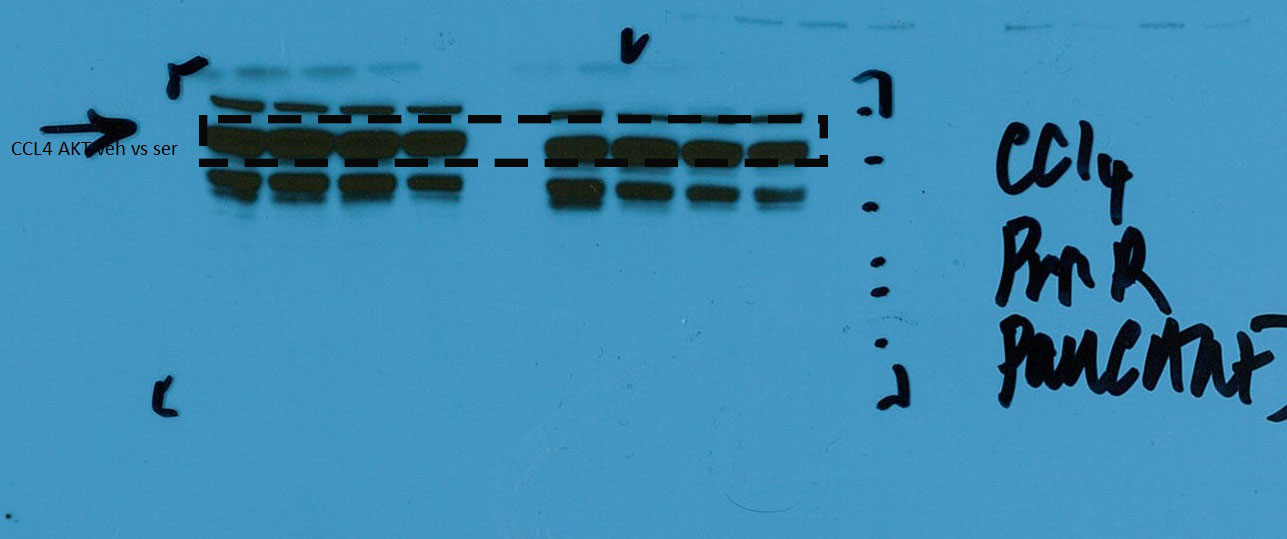

Supplement: S2 Appendix — (ZIP) [file pmed.1002248.s002.zip › AKT CCL4 veh vs serelaxin Fig4A.jpg]

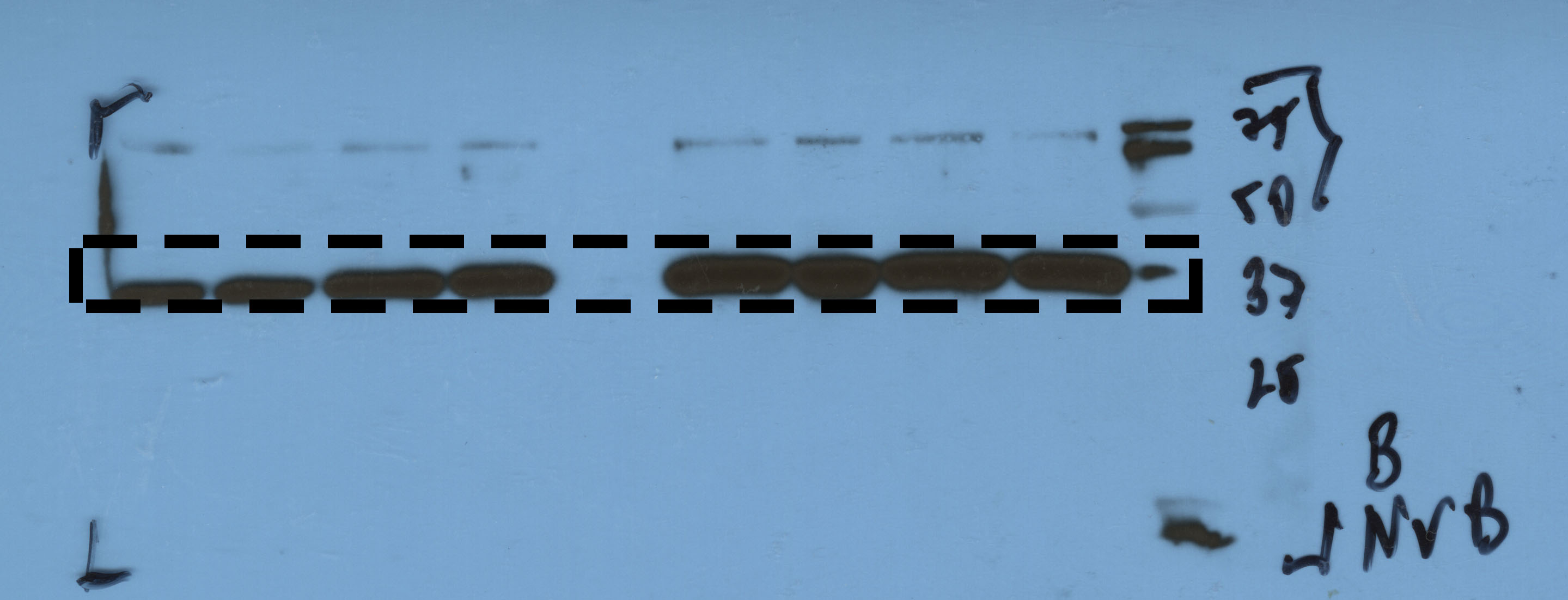

Supplement: S2 Appendix — (ZIP) [file pmed.1002248.s002.zip › Arginase 2 BDL sham vs BDL FigS3F.jpg]

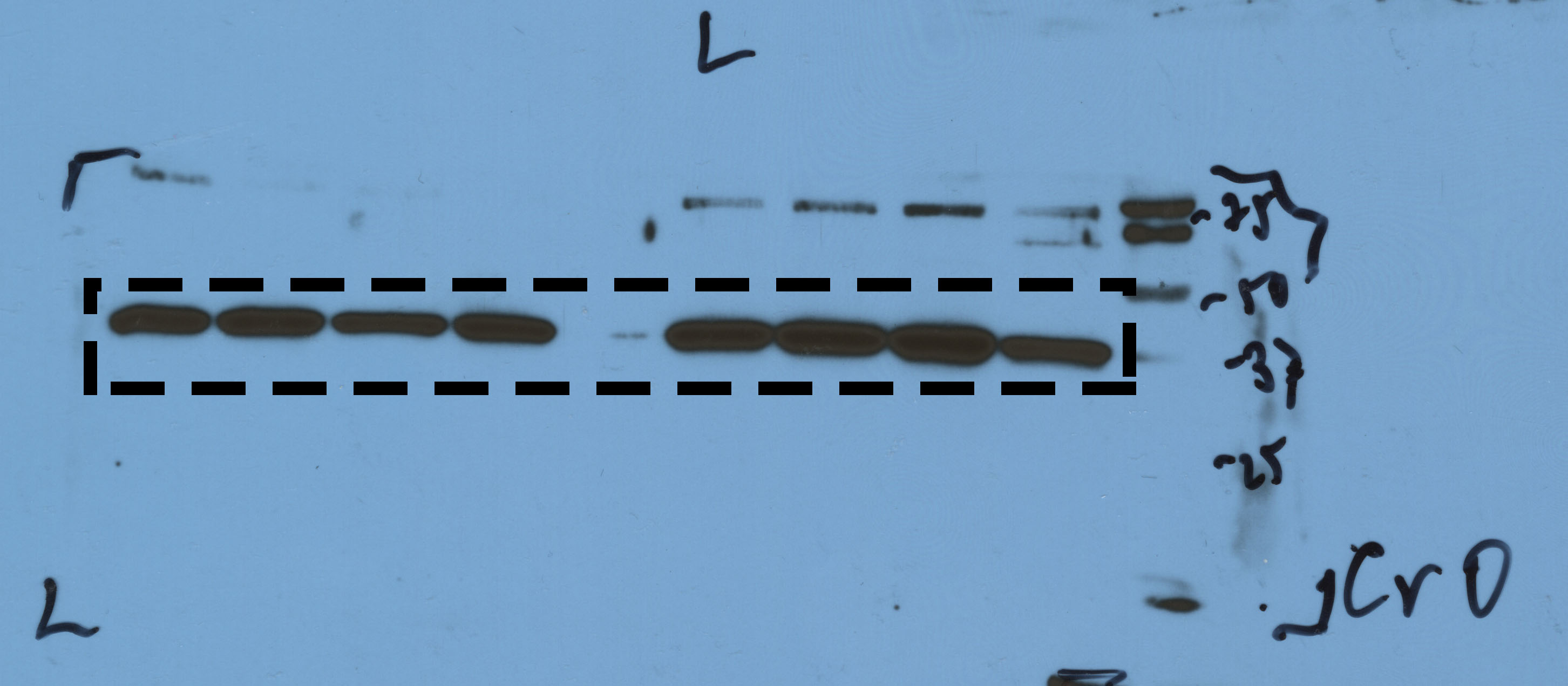

Supplement: S2 Appendix — (ZIP) [file pmed.1002248.s002.zip › Arginase 2 CCL4 olive oil vs CCL4 Fig S3E.jpg]

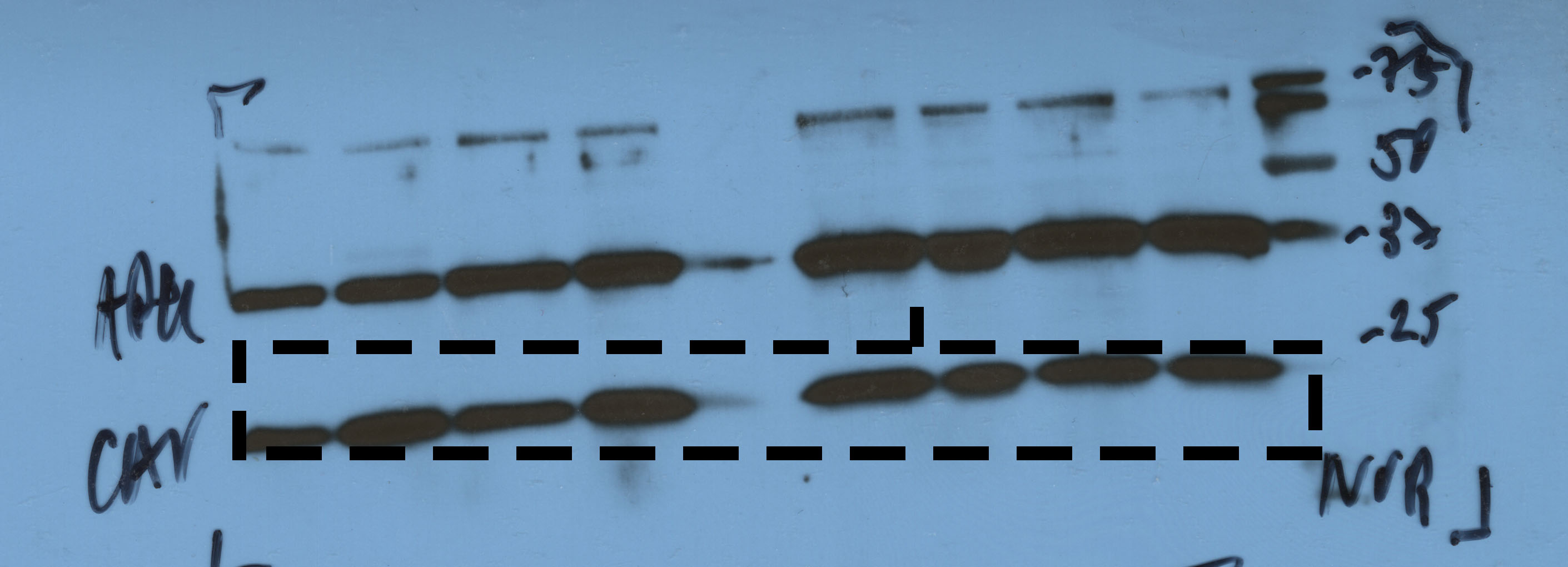

Supplement: S2 Appendix — (ZIP) [file pmed.1002248.s002.zip › Caveolin BDL sham vs BDL Fig S3H.jpg]

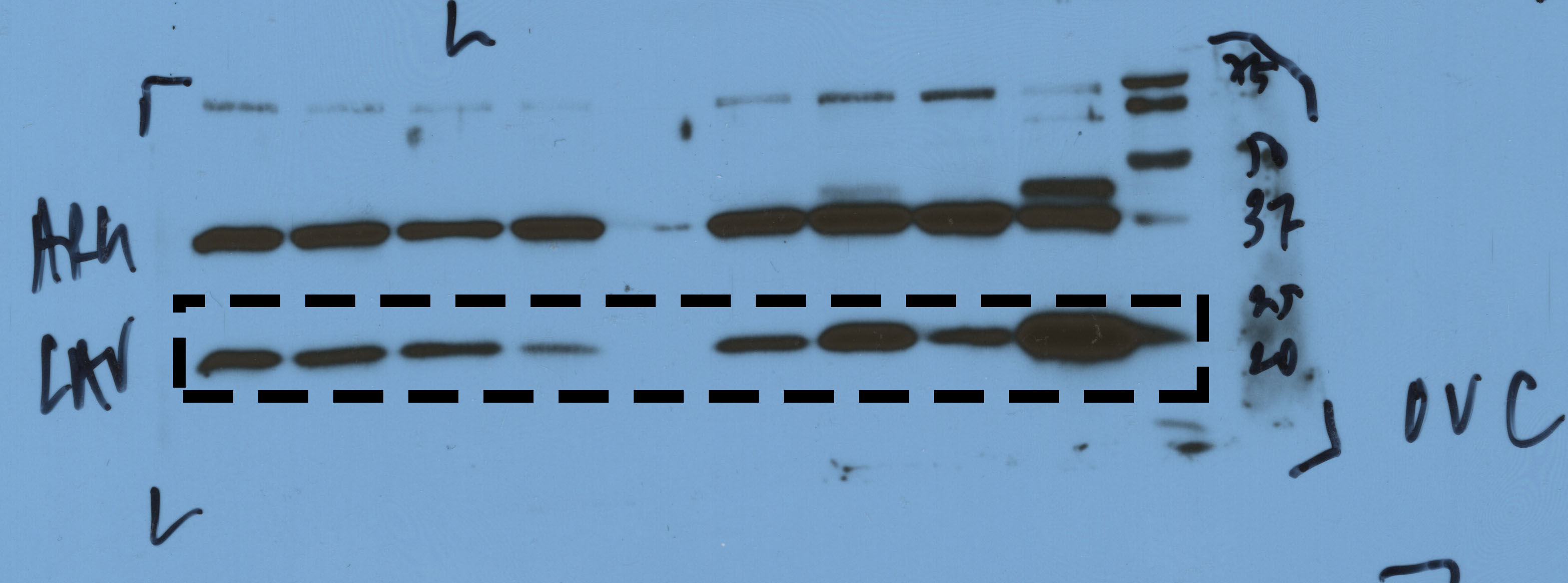

Supplement: S2 Appendix — (ZIP) [file pmed.1002248.s002.zip › Caveolin CCL4 olive oil vs CCL4 Fig S3G.jpg]

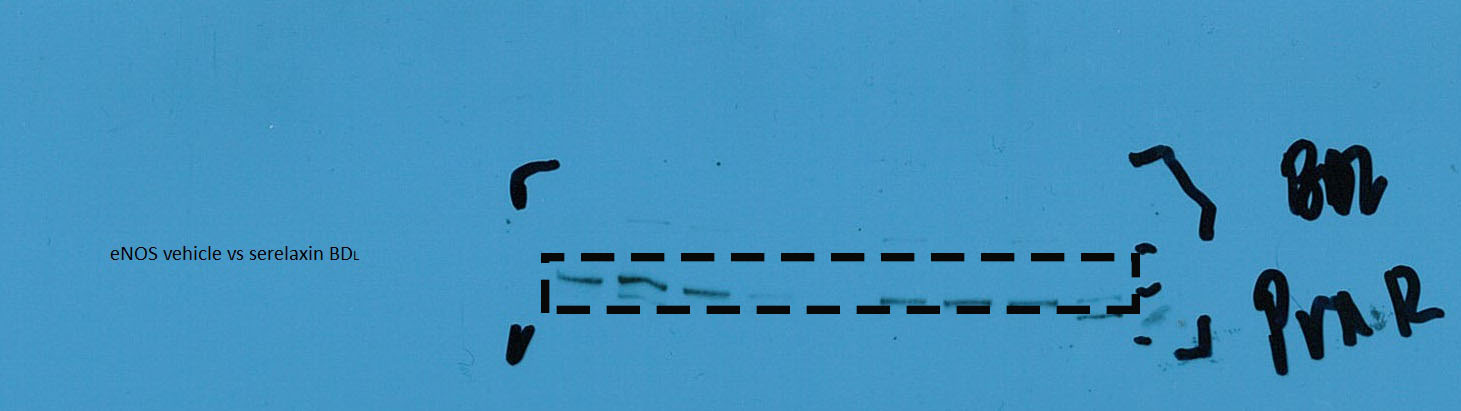

Supplement: S2 Appendix — (ZIP) [file pmed.1002248.s002.zip › eNOS BDL veh vs serelaxin Fig4D.jpg]

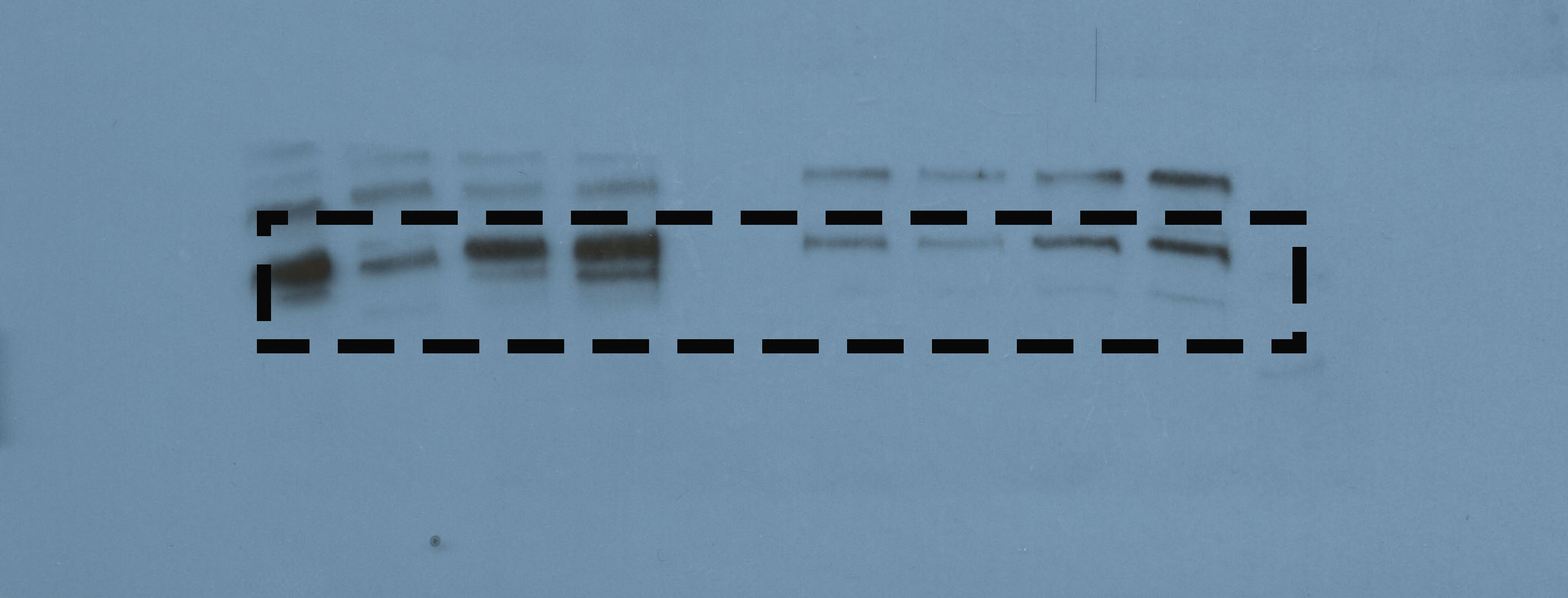

Supplement: S2 Appendix — (ZIP) [file pmed.1002248.s002.zip › ENOS CCL4 + Serelaxin vs CCL4 + Ser + LNAME FigS7A.jpg]

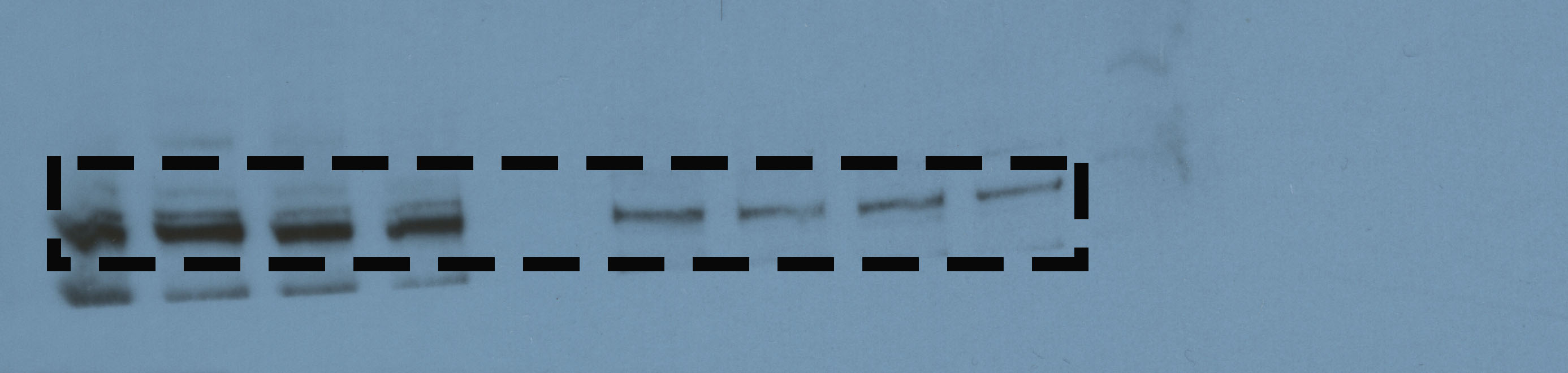

Supplement: S2 Appendix — (ZIP) [file pmed.1002248.s002.zip › ENOS CCL4 + Vehicle vs CCL4 + Veh + LNAME FigS7B.jpg]

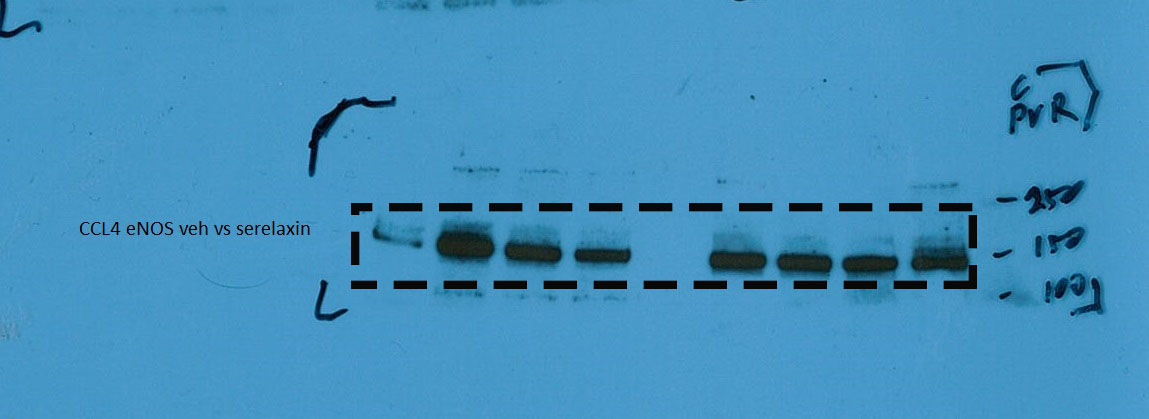

Supplement: S2 Appendix — (ZIP) [file pmed.1002248.s002.zip › eNOS CCL4 veh vs serelaxin Fig4A.jpg]

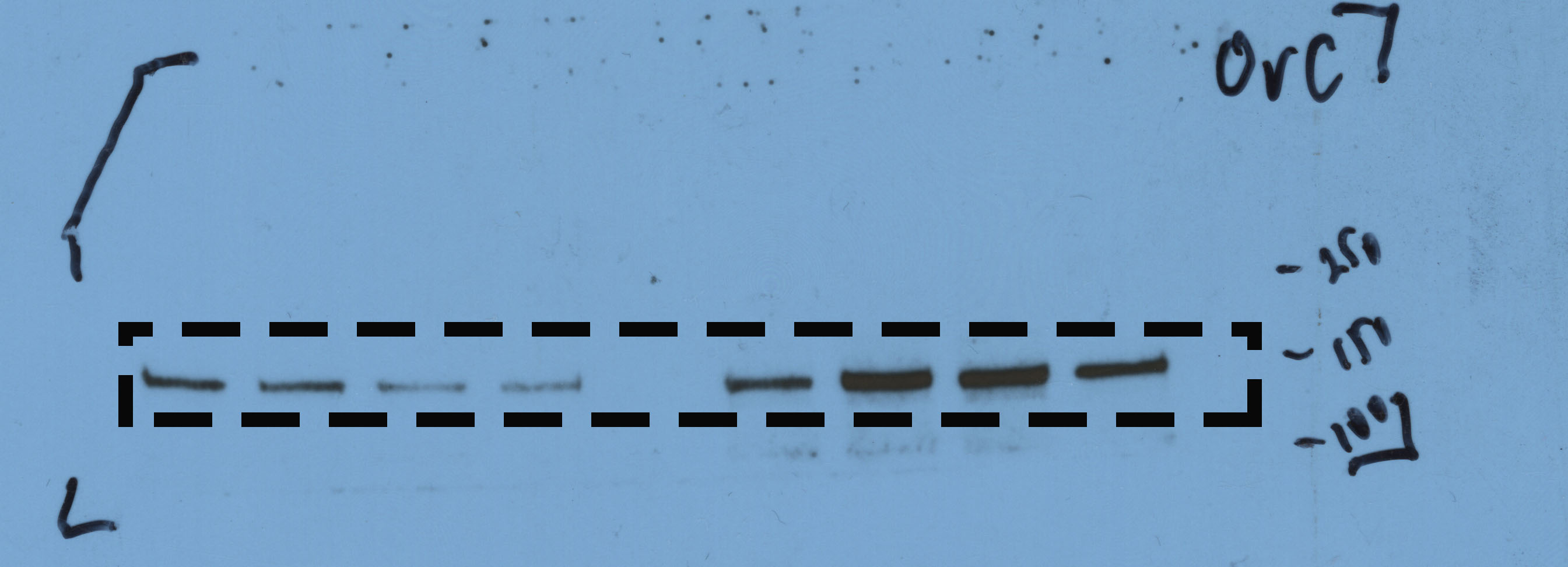

Supplement: S2 Appendix — (ZIP) [file pmed.1002248.s002.zip › ENOS olive oil vs CCL4 FigS3A.jpg]

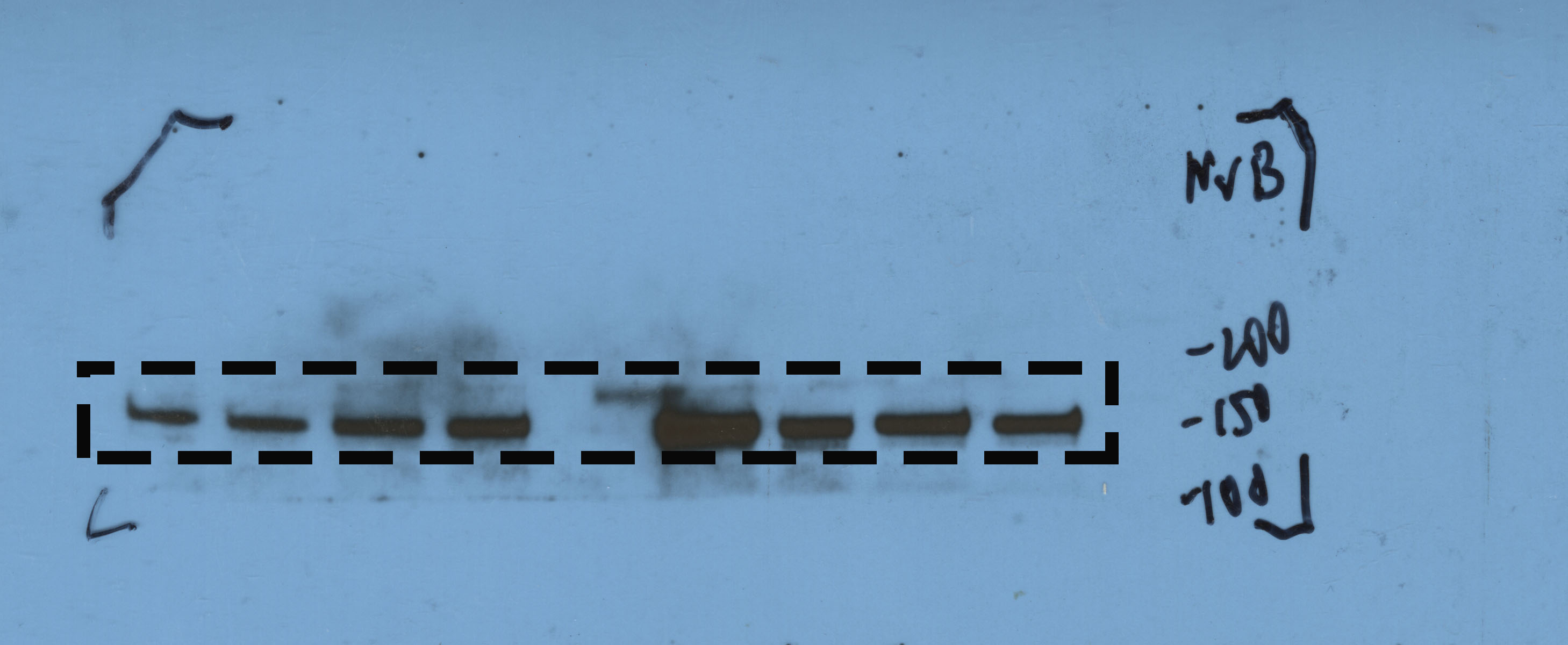

Supplement: S2 Appendix — (ZIP) [file pmed.1002248.s002.zip › ENOS sham vs BDL FigS3B.jpg]

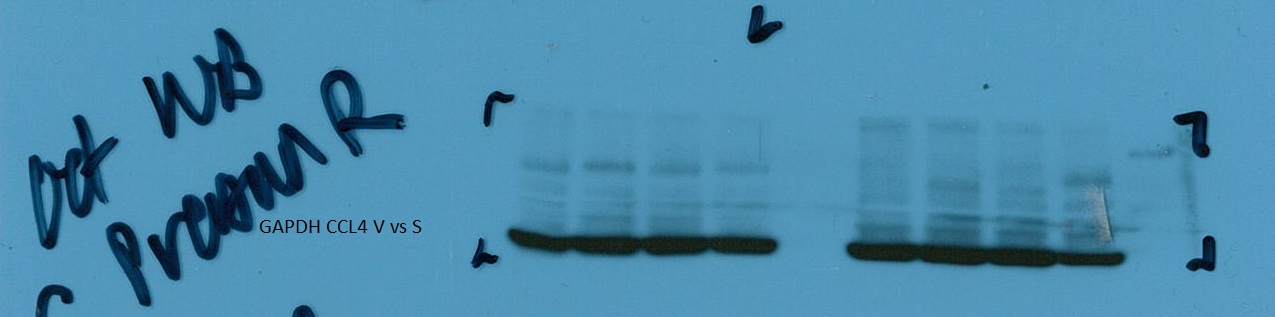

Supplement: S2 Appendix — (ZIP) [file pmed.1002248.s002.zip › GAPDH Fig4A.jpg]

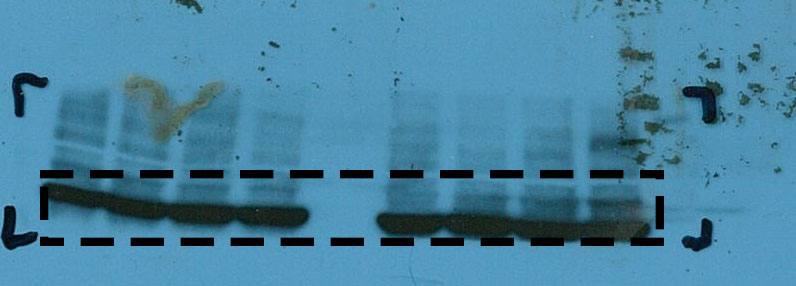

Supplement: S2 Appendix — (ZIP) [file pmed.1002248.s002.zip › GAPDH BDL veh vs serelaxin Fig4D.jpg]

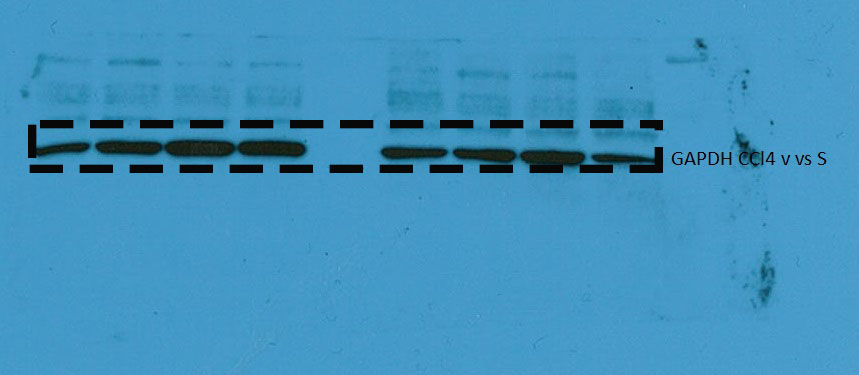

Supplement: S2 Appendix — (ZIP) [file pmed.1002248.s002.zip › GAPDH Fig4A.1.jpg]

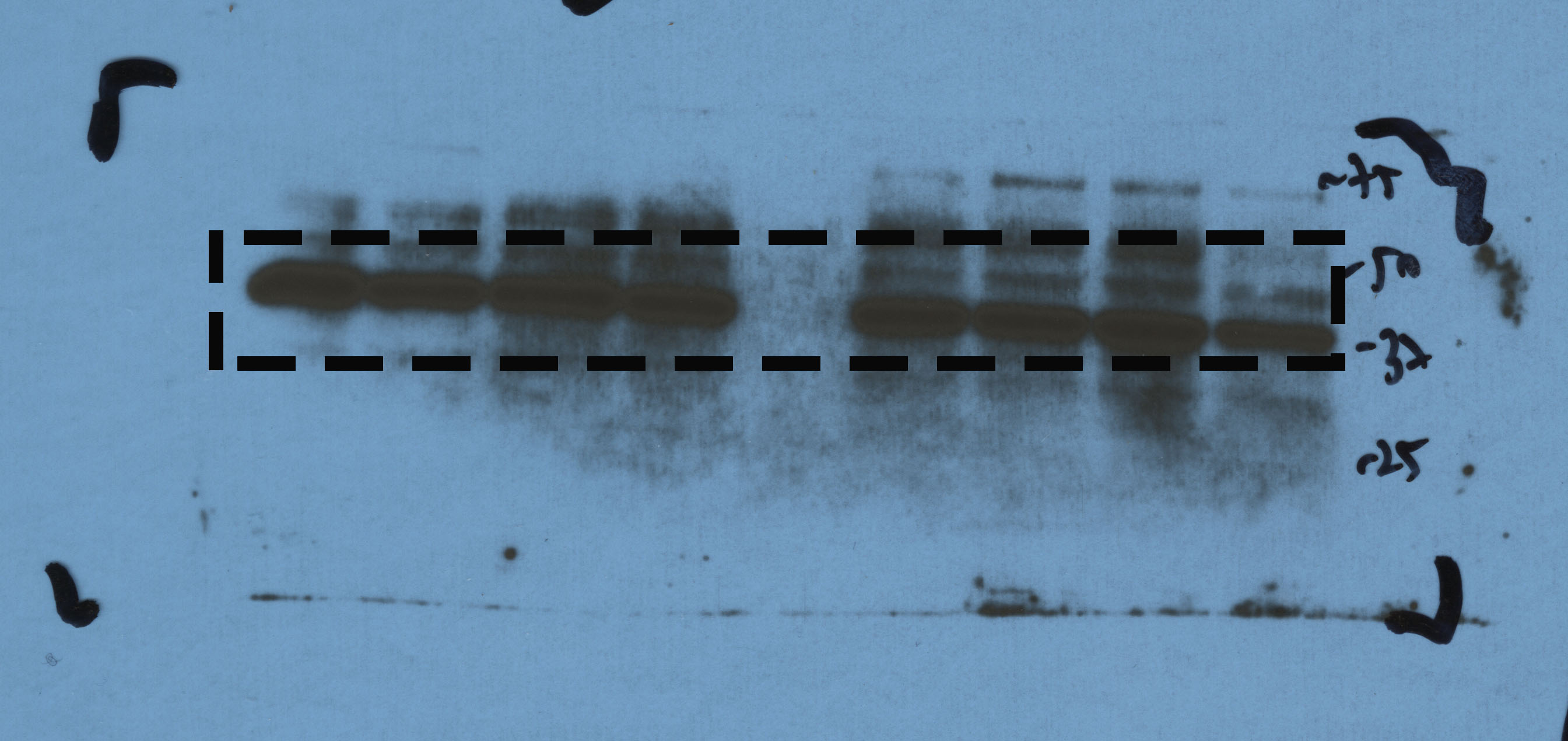

Supplement: S2 Appendix — (ZIP) [file pmed.1002248.s002.zip › GAPDH FigS3A.jpg]

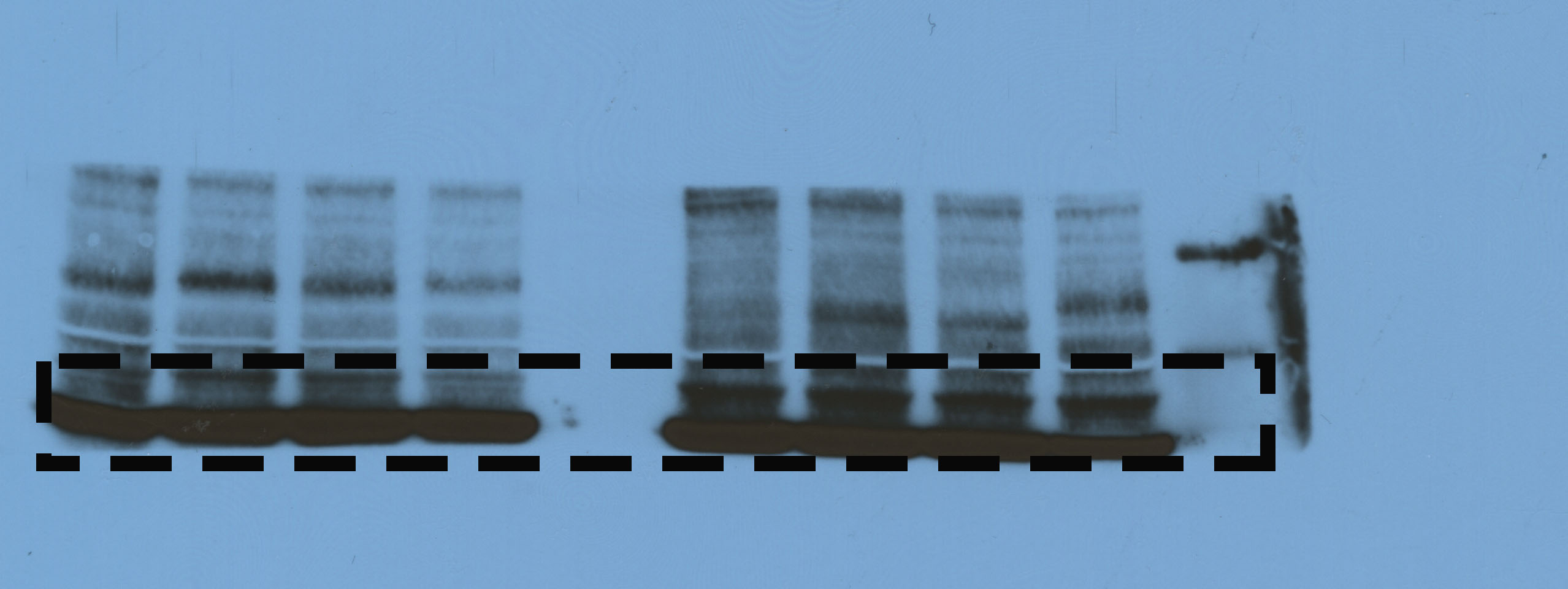

Supplement: S2 Appendix — (ZIP) [file pmed.1002248.s002.zip › GAPDH FigS3B.jpg]

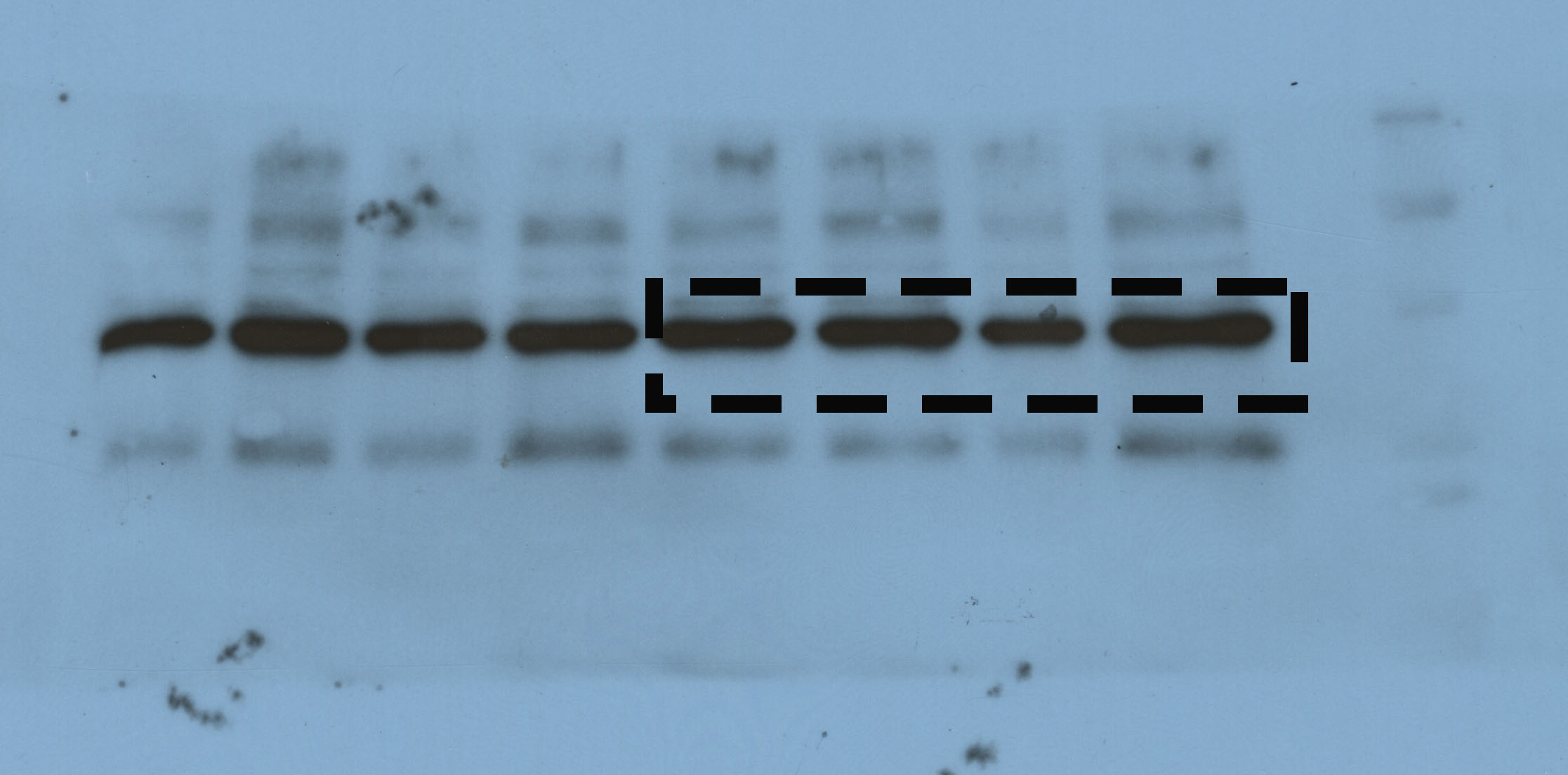

Supplement: S2 Appendix — (ZIP) [file pmed.1002248.s002.zip › GAPDH FigS4B.jpg]

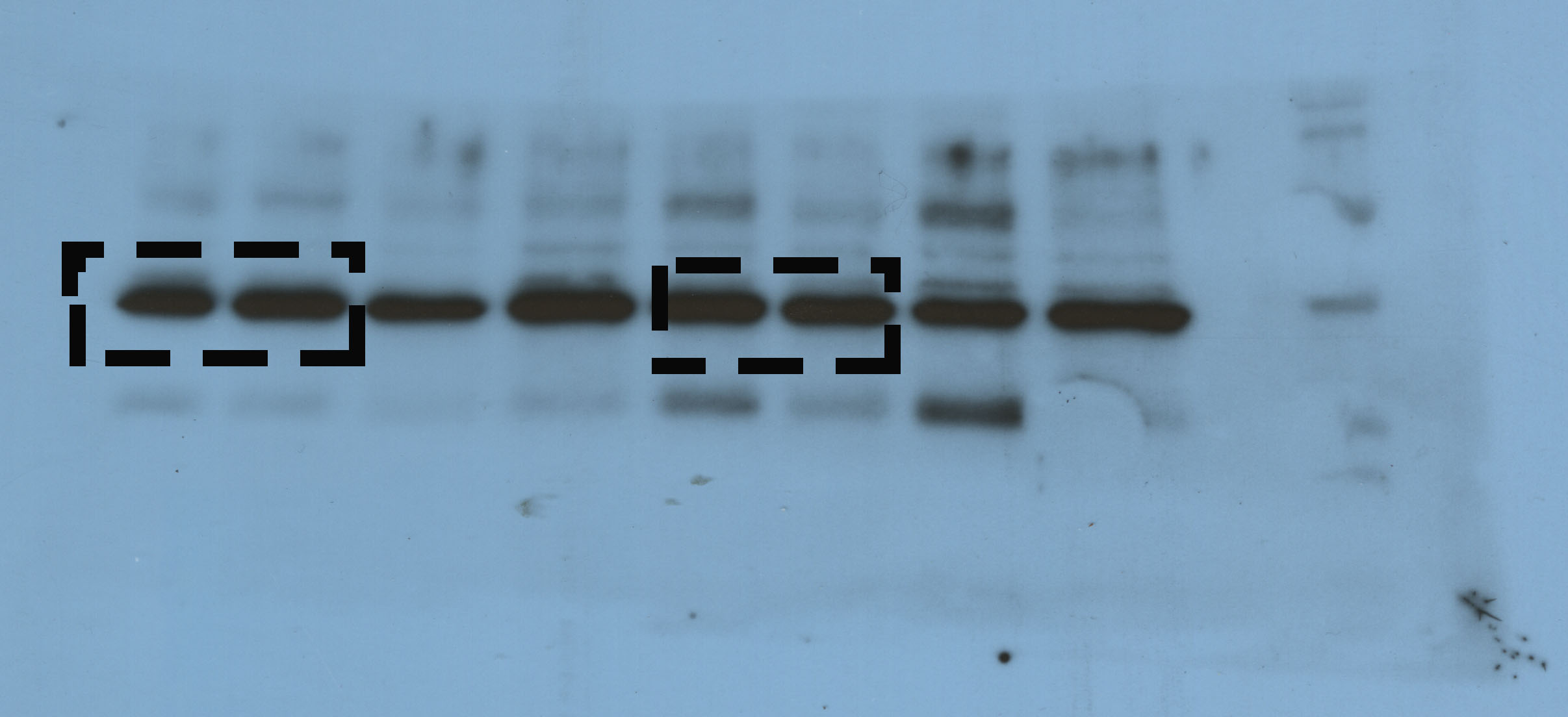

Supplement: S2 Appendix — (ZIP) [file pmed.1002248.s002.zip › GAPDH FigS4D.jpg]

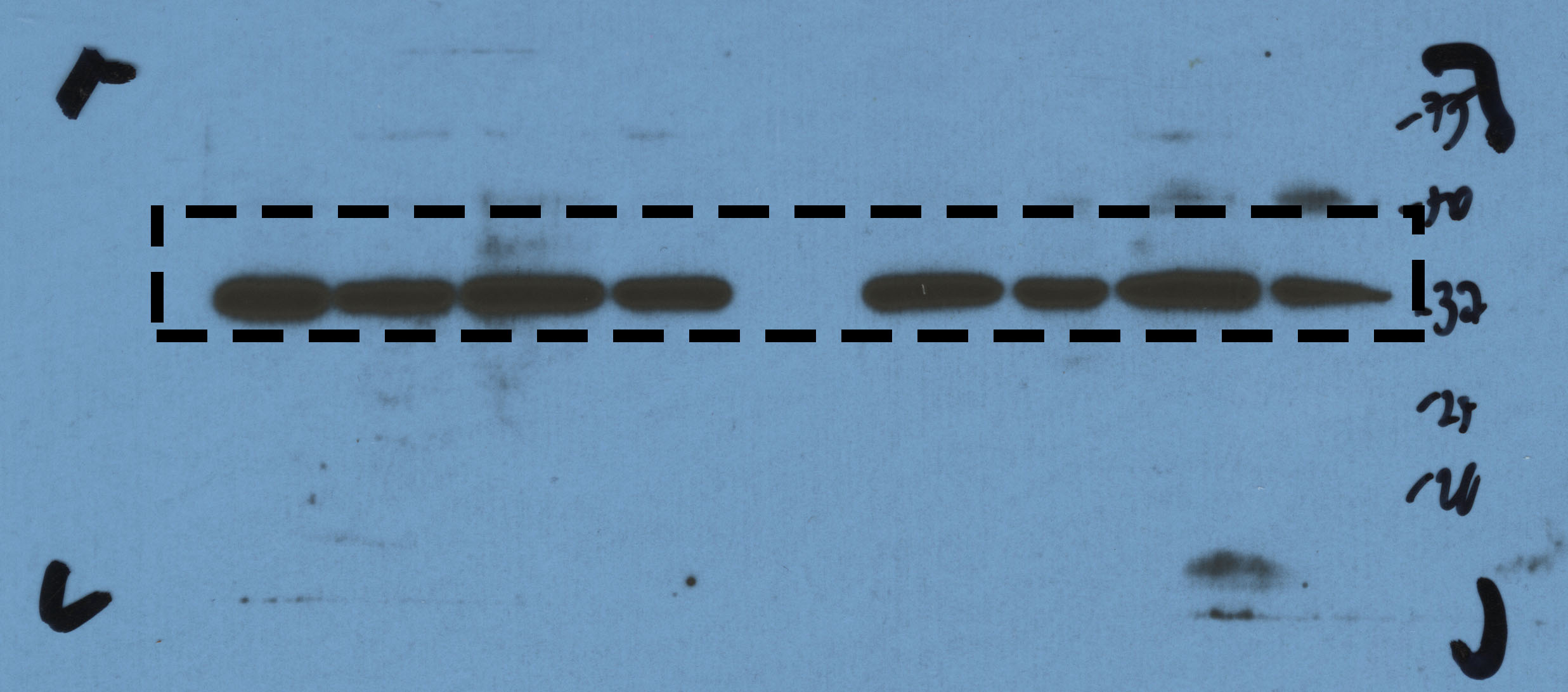

Supplement: S2 Appendix — (ZIP) [file pmed.1002248.s002.zip › GAPDH FigS7A.jpg]

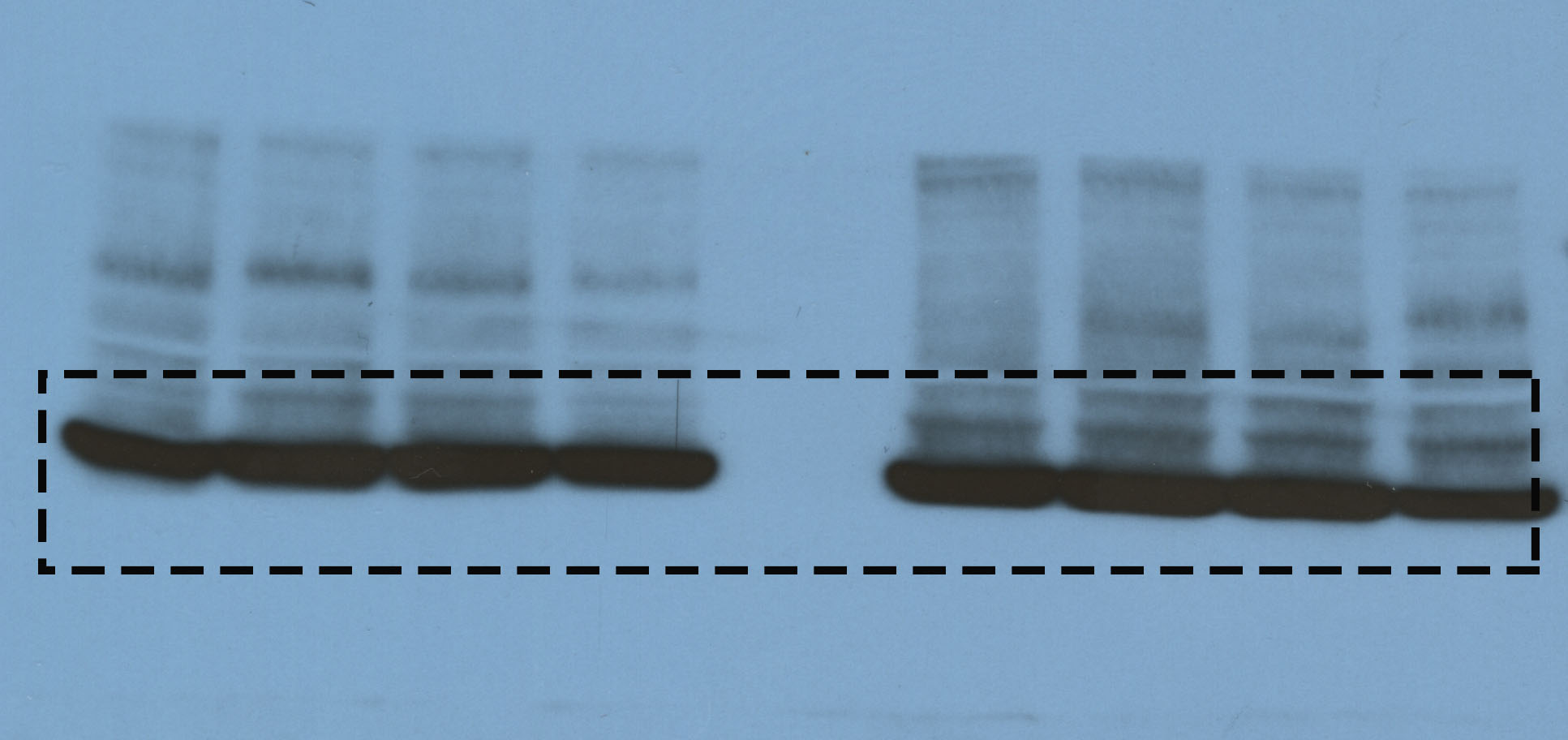

Supplement: S2 Appendix — (ZIP) [file pmed.1002248.s002.zip › GAPDH FigS7B.jpg]

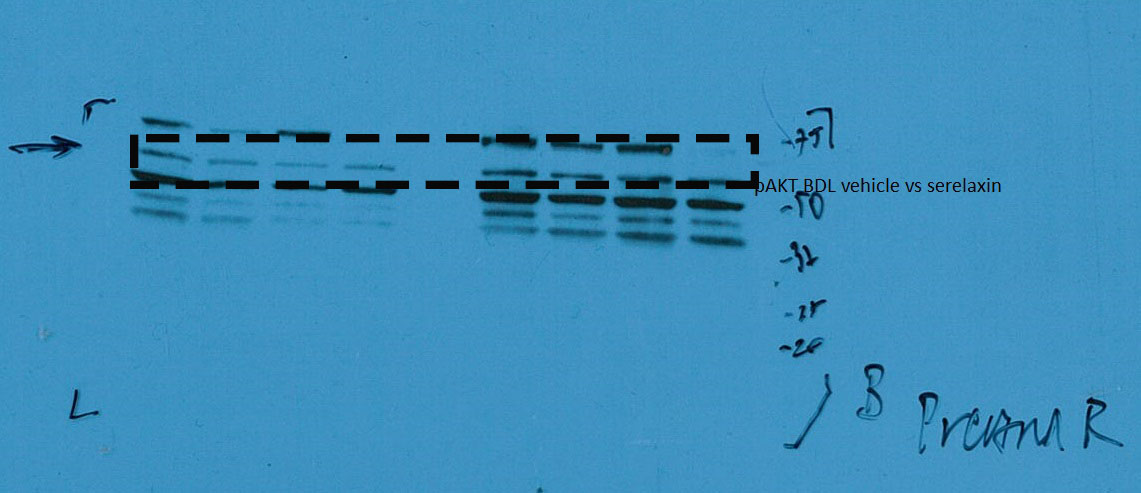

Supplement: S2 Appendix — (ZIP) [file pmed.1002248.s002.zip › p-AKT BDL veh vs serelaxin Fig4D.jpg]

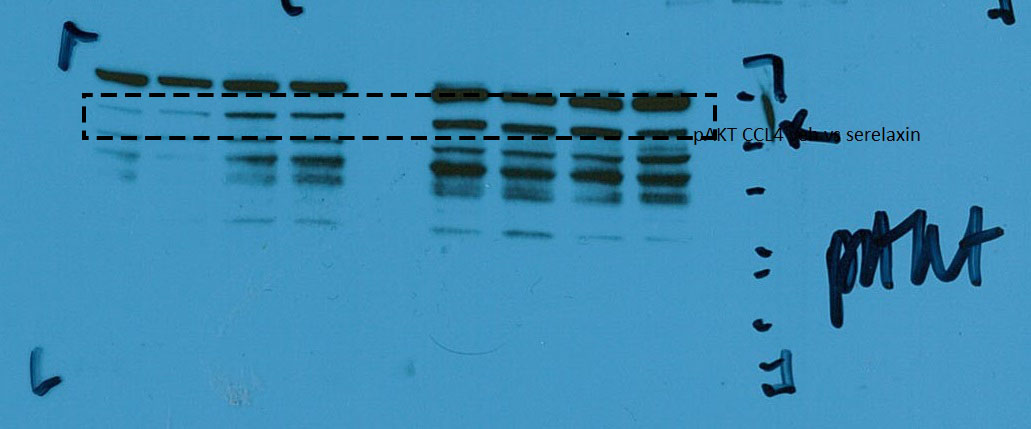

Supplement: S2 Appendix — (ZIP) [file pmed.1002248.s002.zip › p-AKT CCL4 veh vs serelaxin Fig4A.jpg]

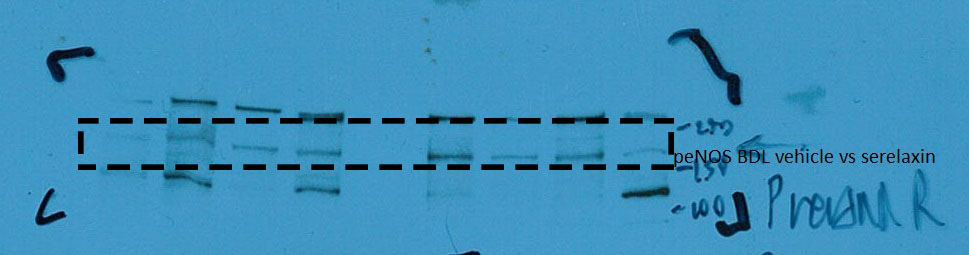

Supplement: S2 Appendix — (ZIP) [file pmed.1002248.s002.zip › p-eNOS BDL veh vs serelaxin Fig4D.jpg]

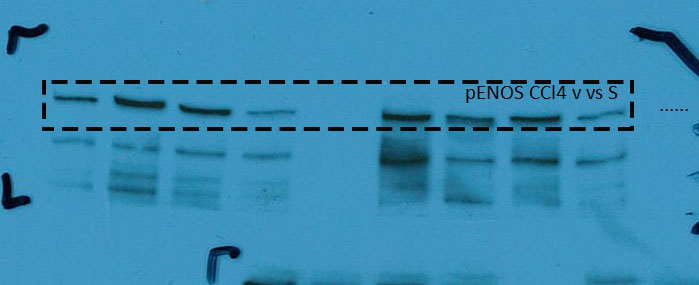

Supplement: S2 Appendix — (ZIP) [file pmed.1002248.s002.zip › p-eNOS CCL4 veh vs serelaxin Fig4A.jpg]

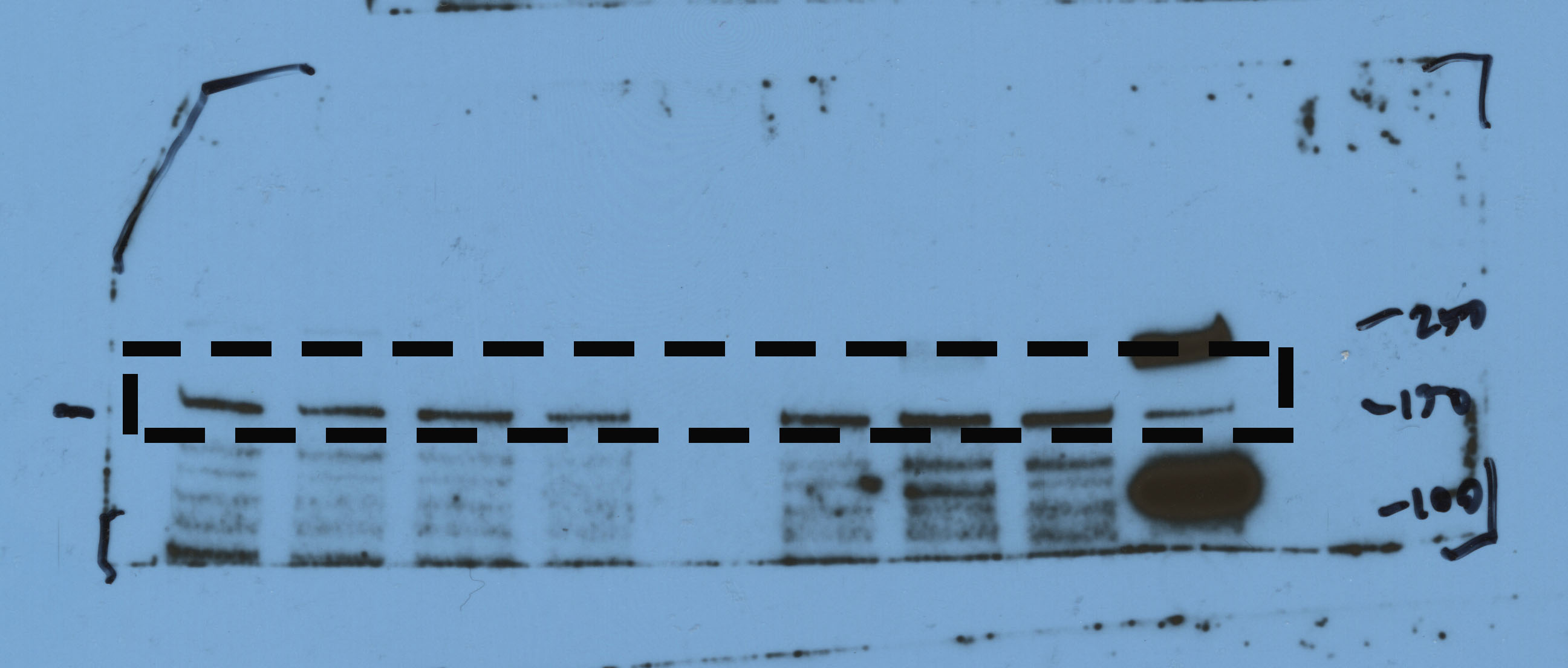

Supplement: S2 Appendix — (ZIP) [file pmed.1002248.s002.zip › p-ENOS olive oil vs CCL4 FigS3A.jpg]

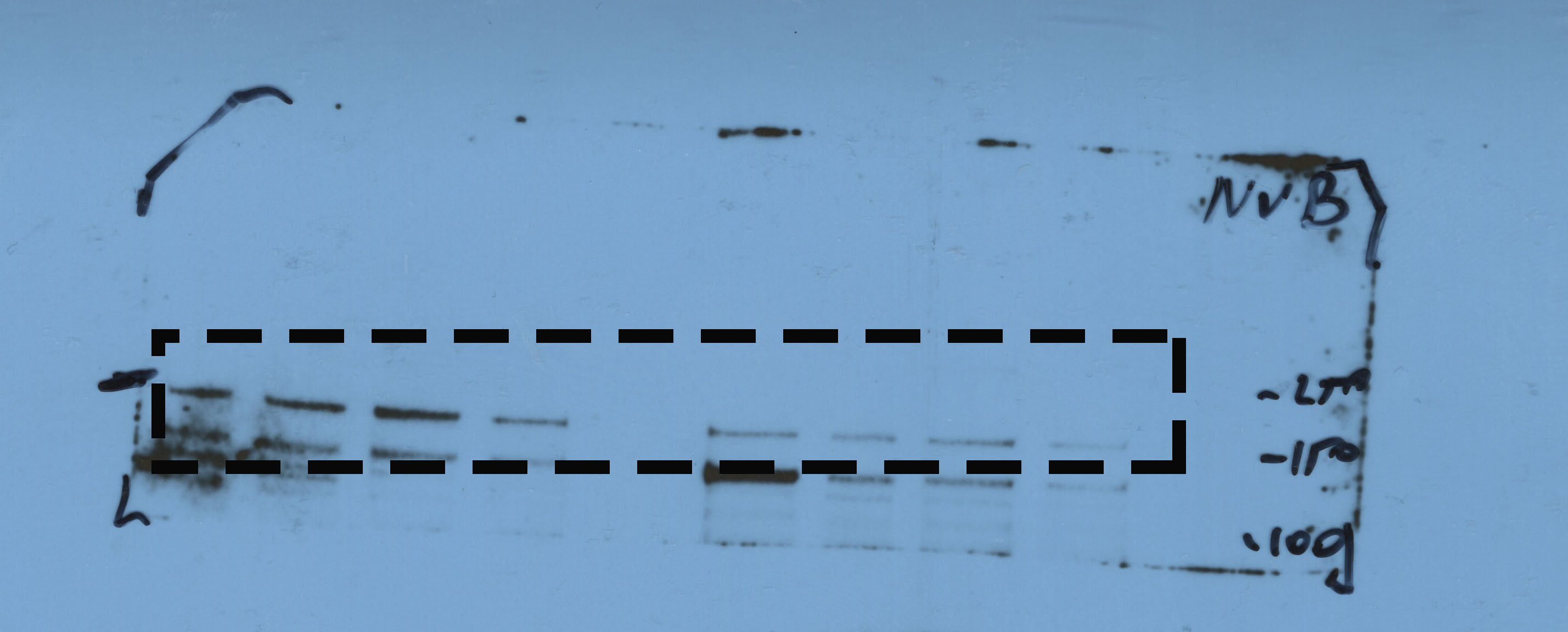

Supplement: S2 Appendix — (ZIP) [file pmed.1002248.s002.zip › p-ENOS sham vs BDL FigS3B.jpg]

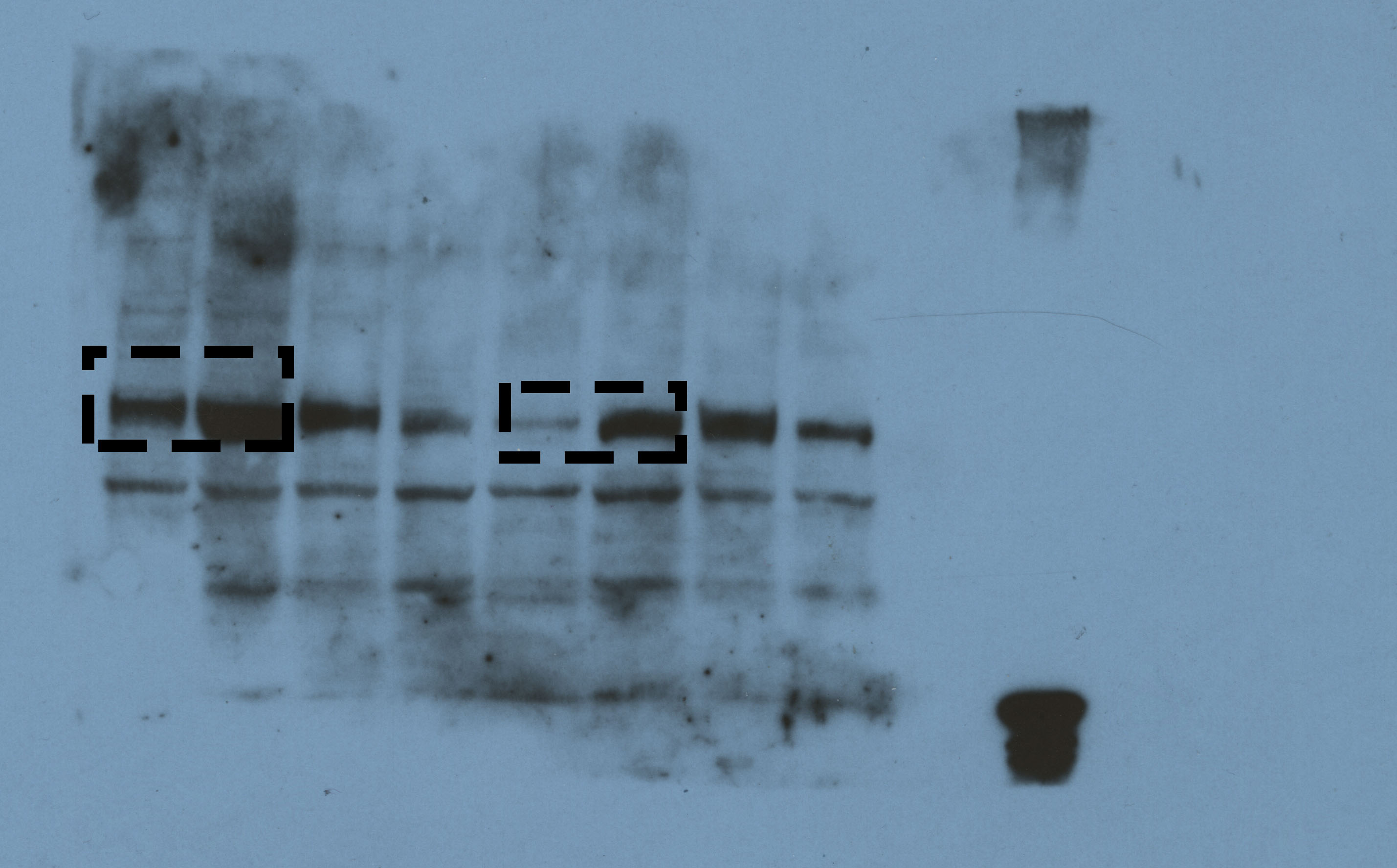

Supplement: S2 Appendix — (ZIP) [file pmed.1002248.s002.zip › RXFP BDL sham vs BDL Fig S4D.jpg]

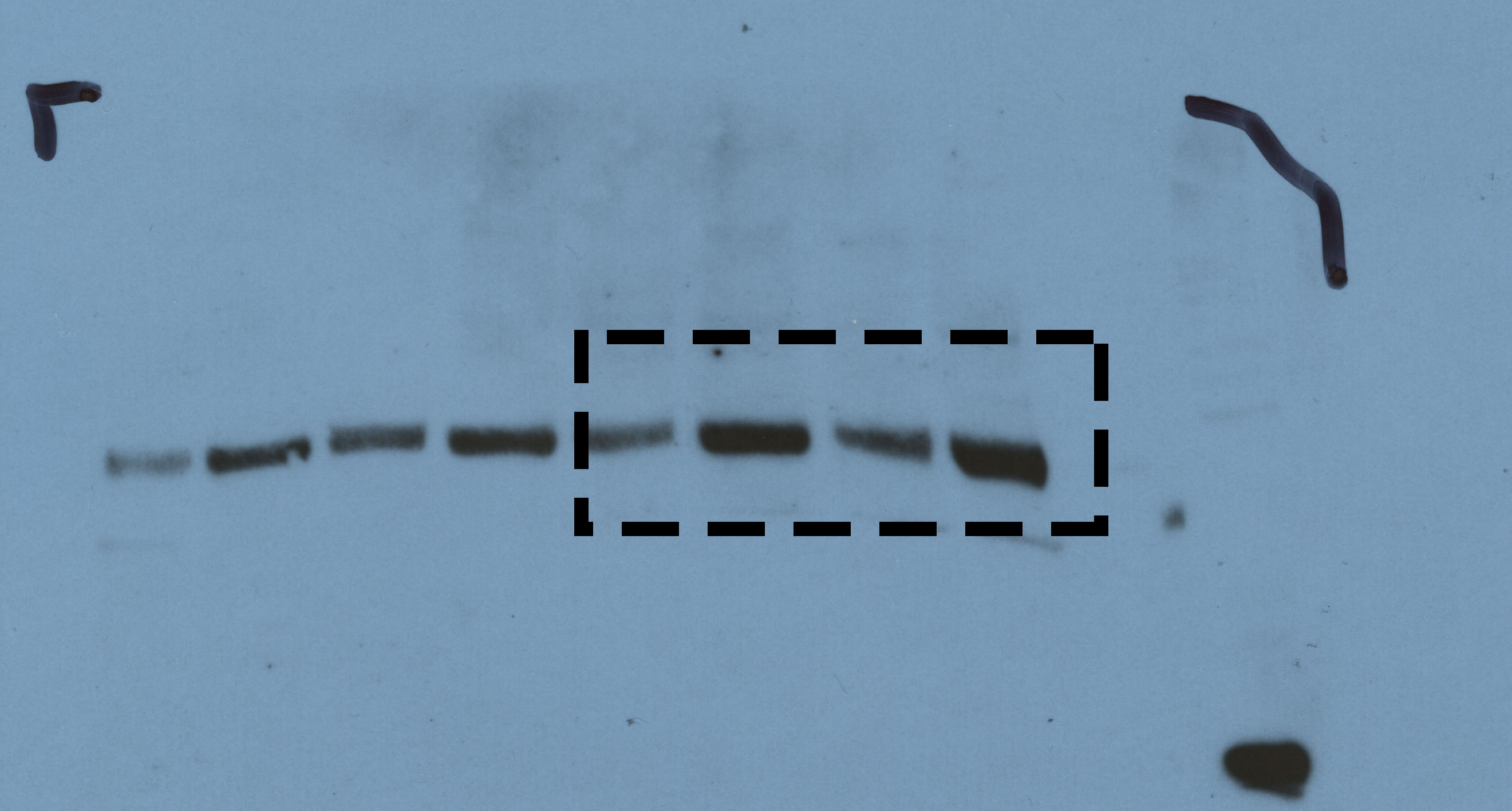

Supplement: S2 Appendix — (ZIP) [file pmed.1002248.s002.zip › RXFP CCl4 olive oil vs CCL4 Fig S4B.jpg]

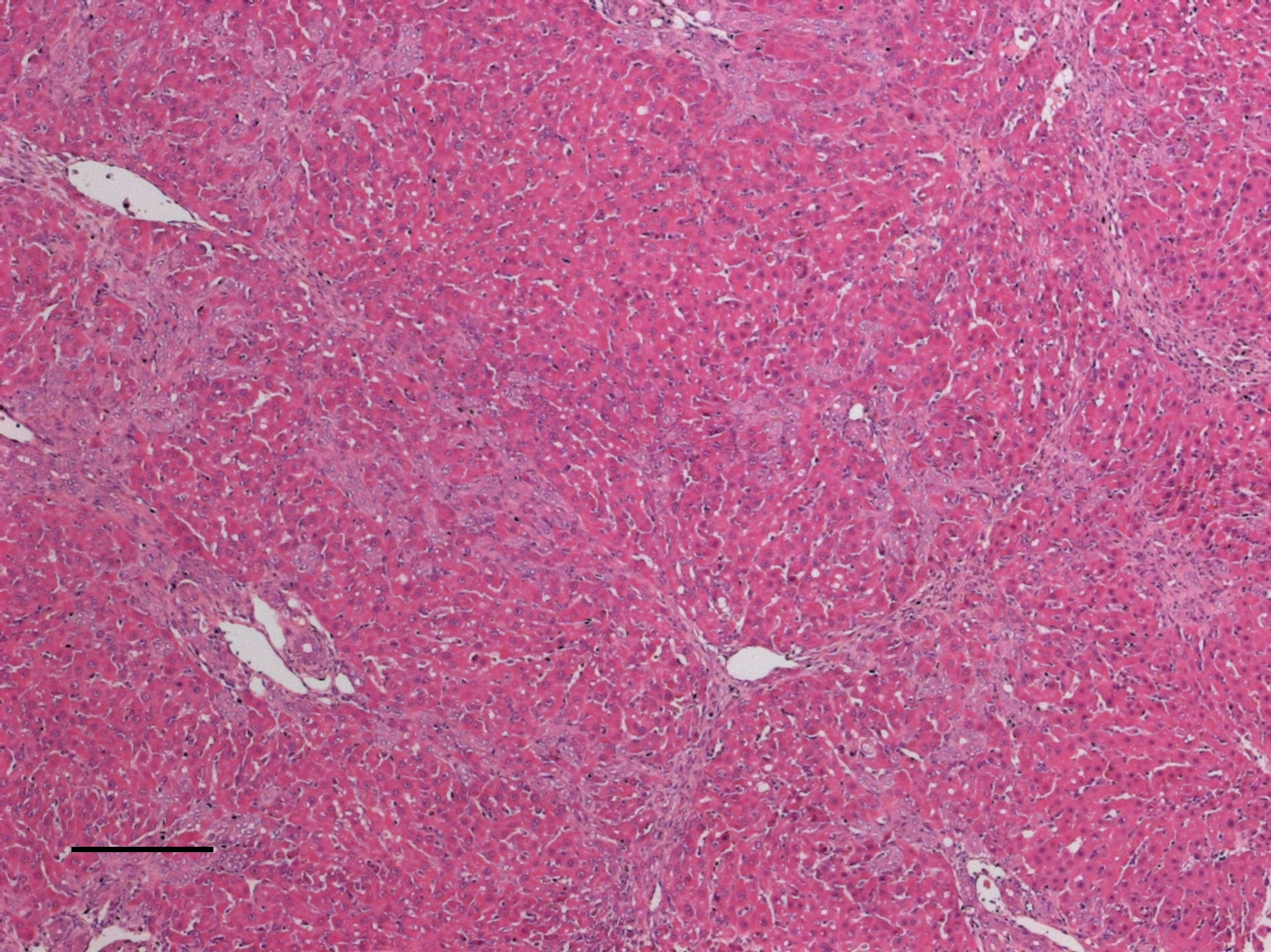

Supplement: S3 Appendix — (ZIP) [file pmed.1002248.s003.zip › BDL wk2 h&e.jpg]

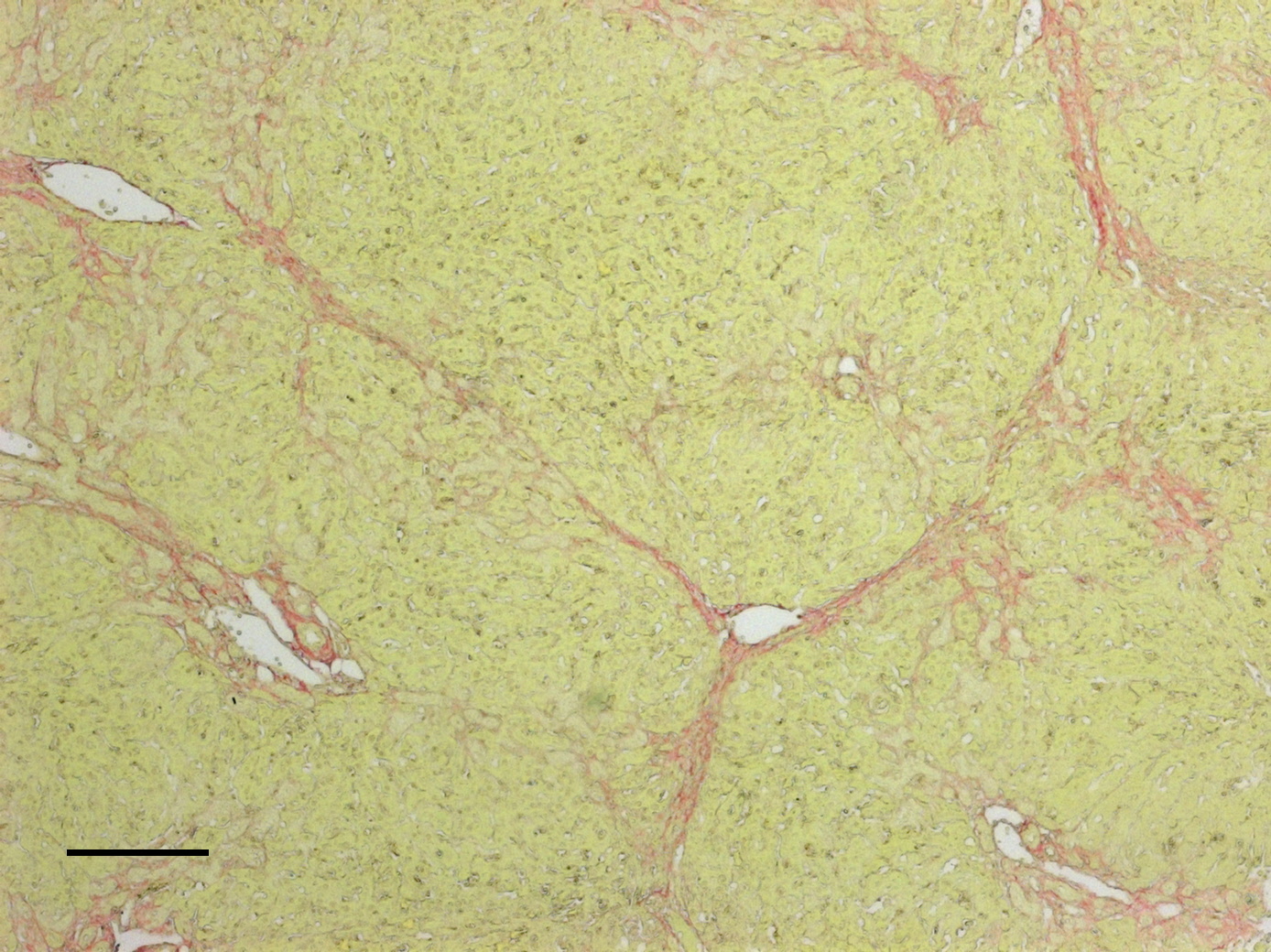

Supplement: S3 Appendix — (ZIP) [file pmed.1002248.s003.zip › BDL wk2 psr.jpg]

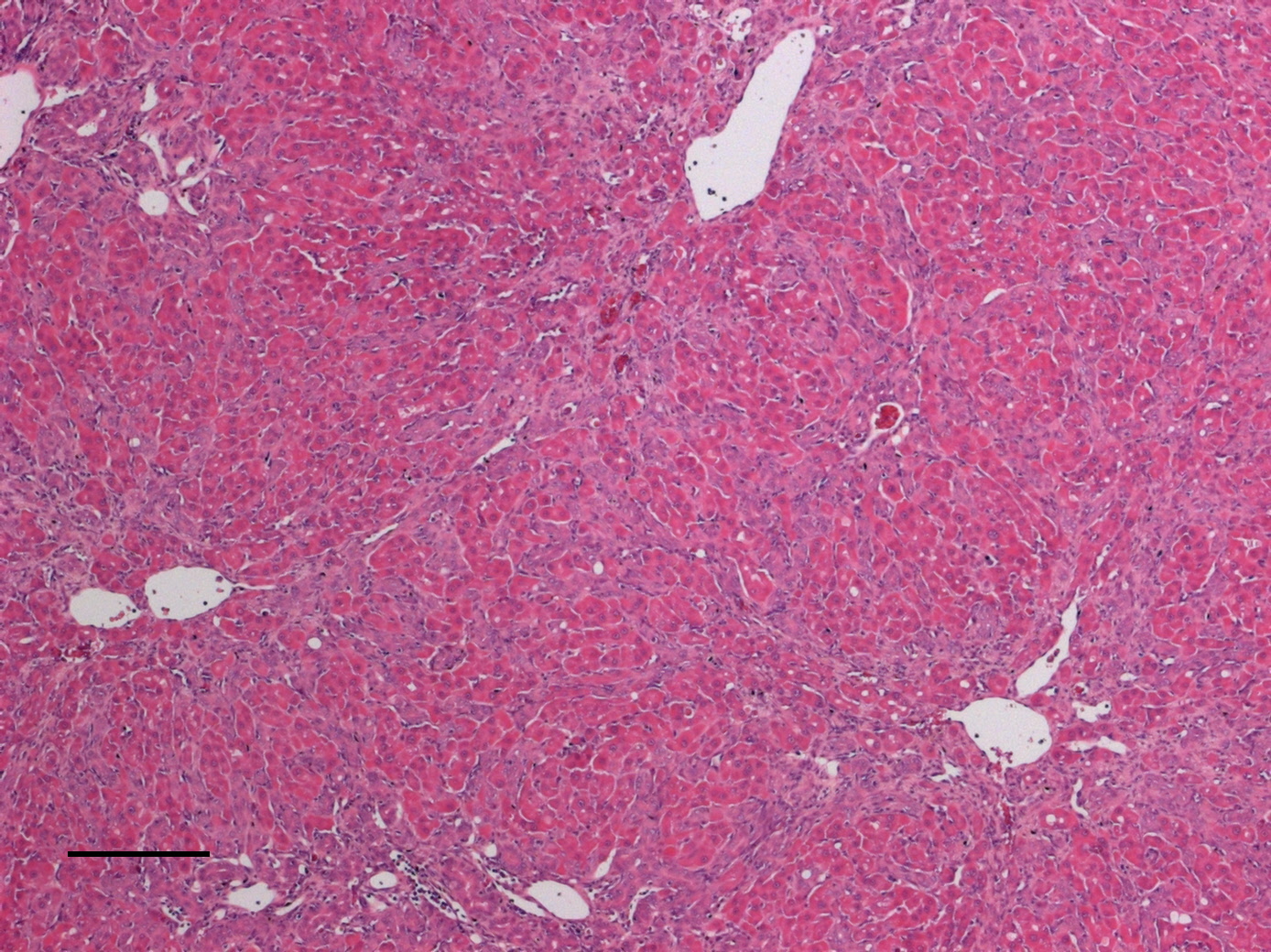

Supplement: S3 Appendix — (ZIP) [file pmed.1002248.s003.zip › BDL wk3 h&e.jpg]

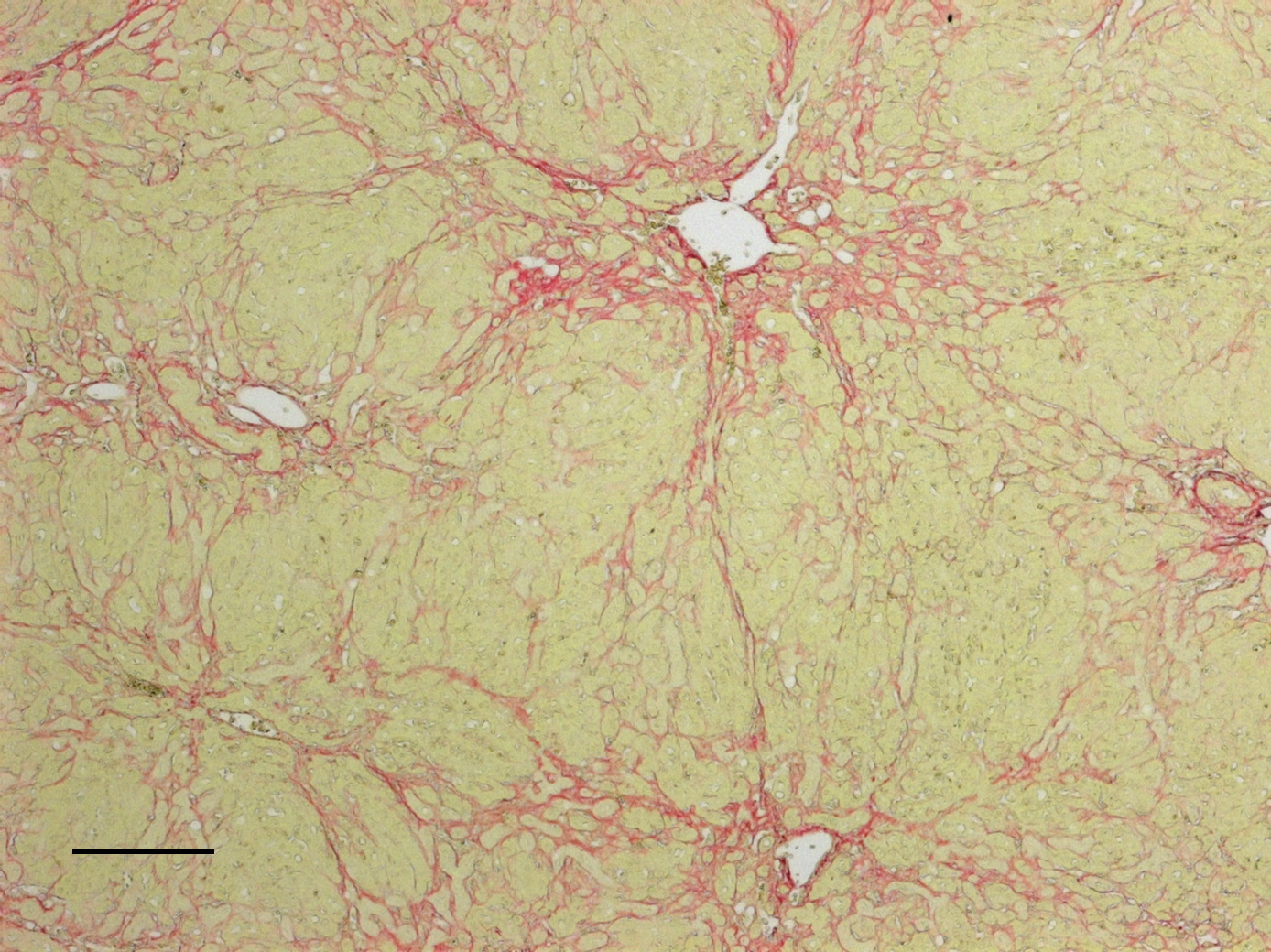

Supplement: S3 Appendix — (ZIP) [file pmed.1002248.s003.zip › BDL wk3 psr.jpg]

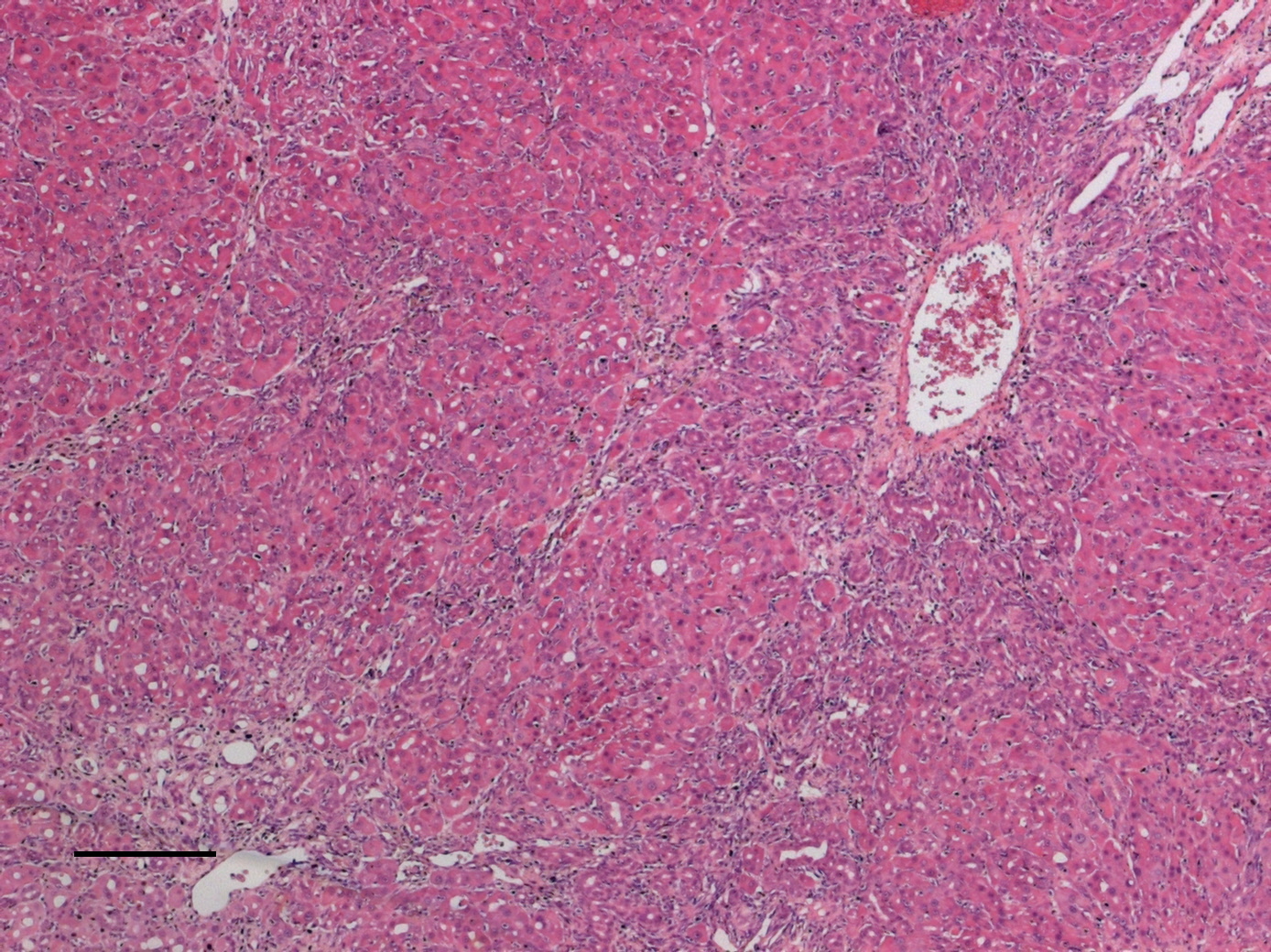

Supplement: S3 Appendix — (ZIP) [file pmed.1002248.s003.zip › BDL wk4 h&e.jpg]

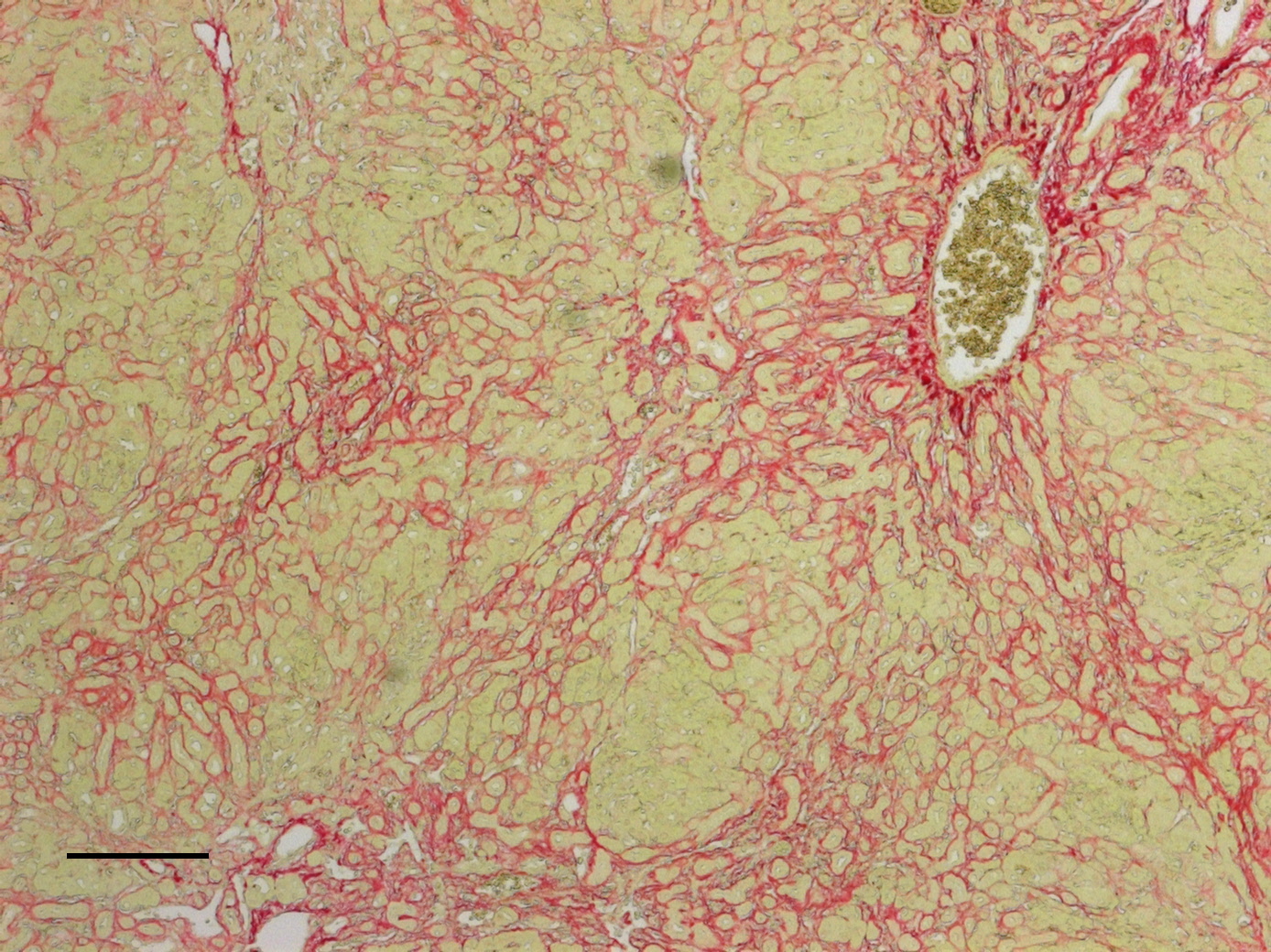

Supplement: S3 Appendix — (ZIP) [file pmed.1002248.s003.zip › BDL wk4 psr.jpg]

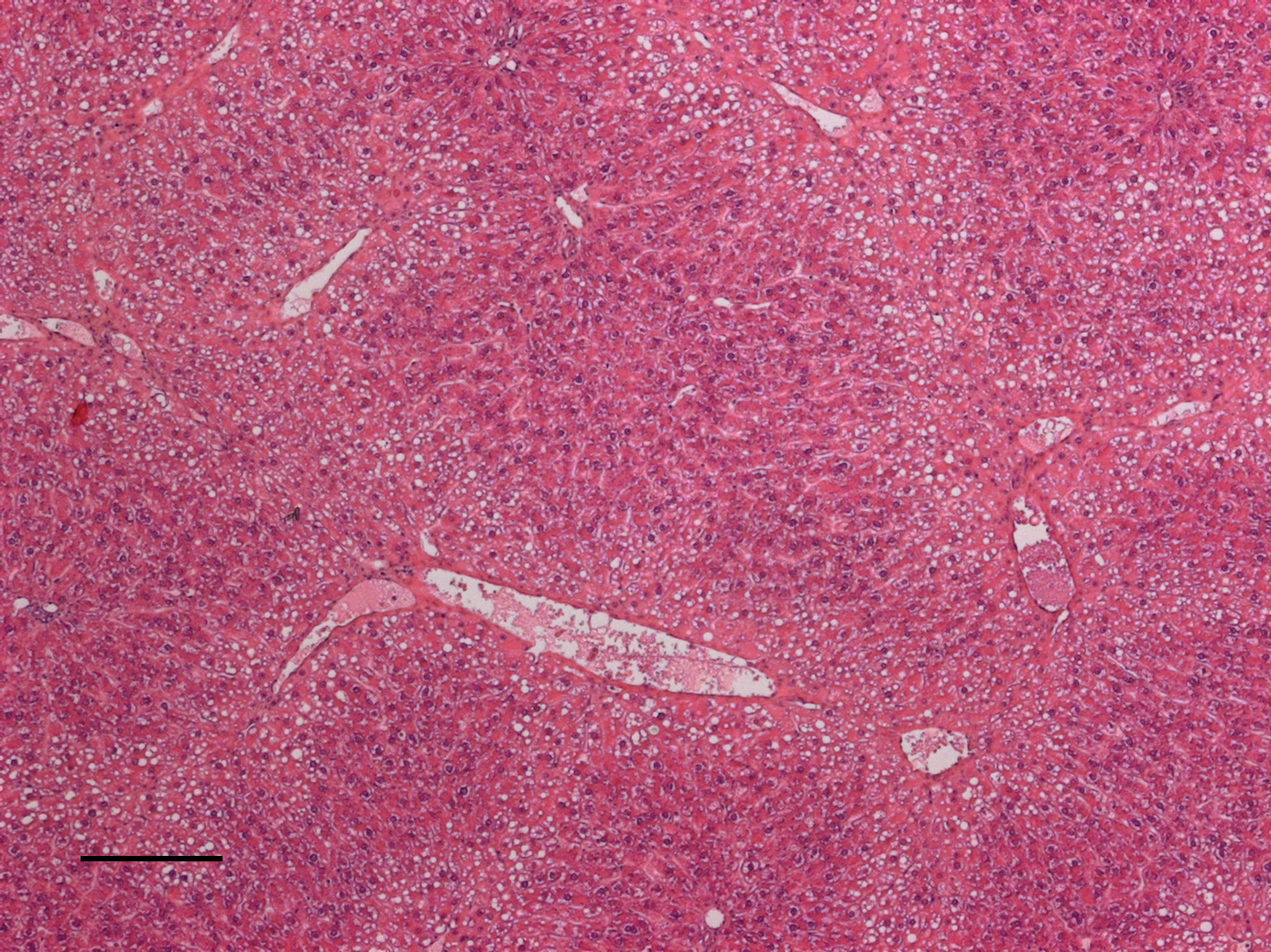

Supplement: S3 Appendix — (ZIP) [file pmed.1002248.s003.zip › CCL4 16wk he.jpg]

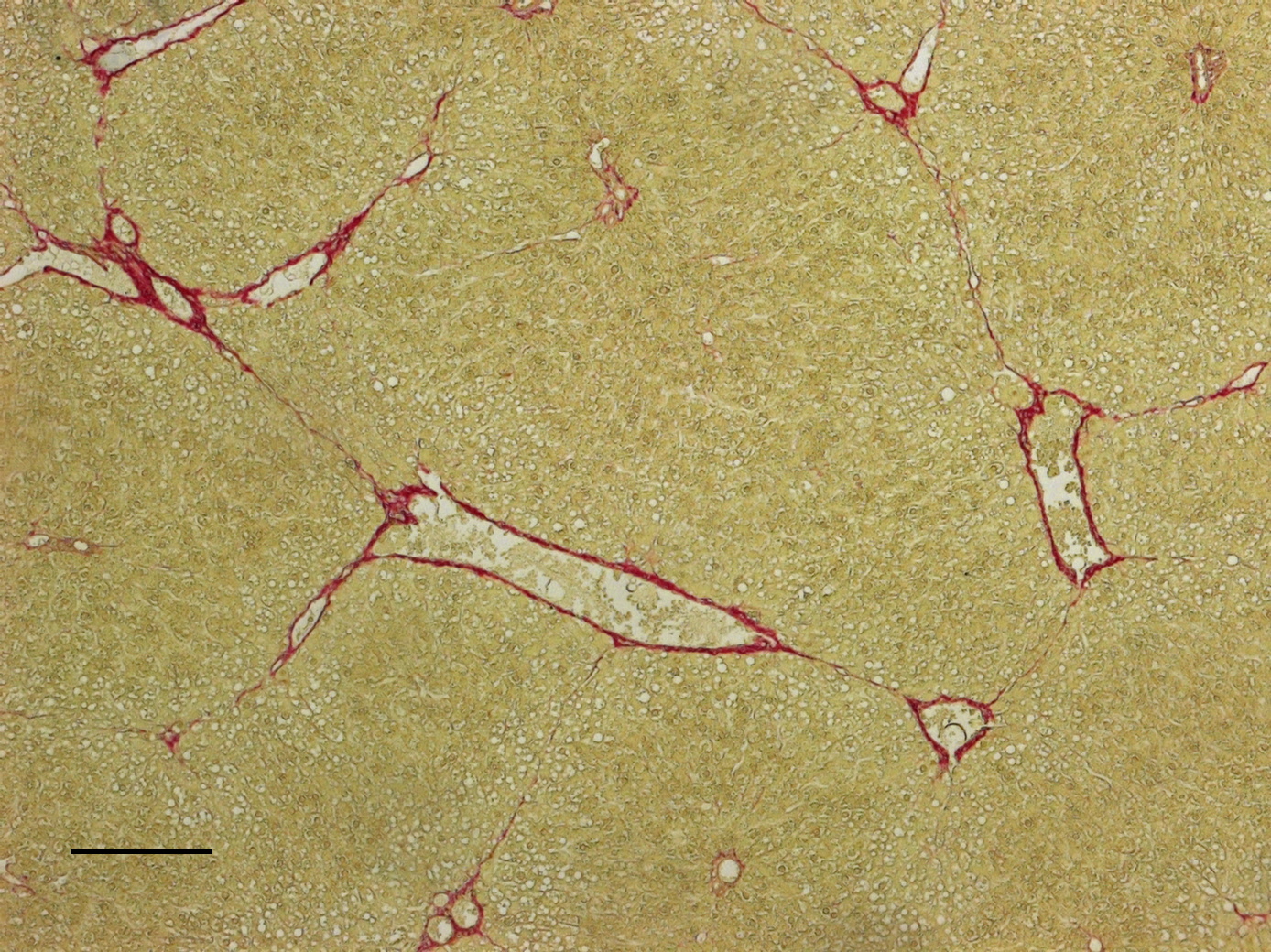

Supplement: S3 Appendix — (ZIP) [file pmed.1002248.s003.zip › CCL4 16wk psr.jpg]

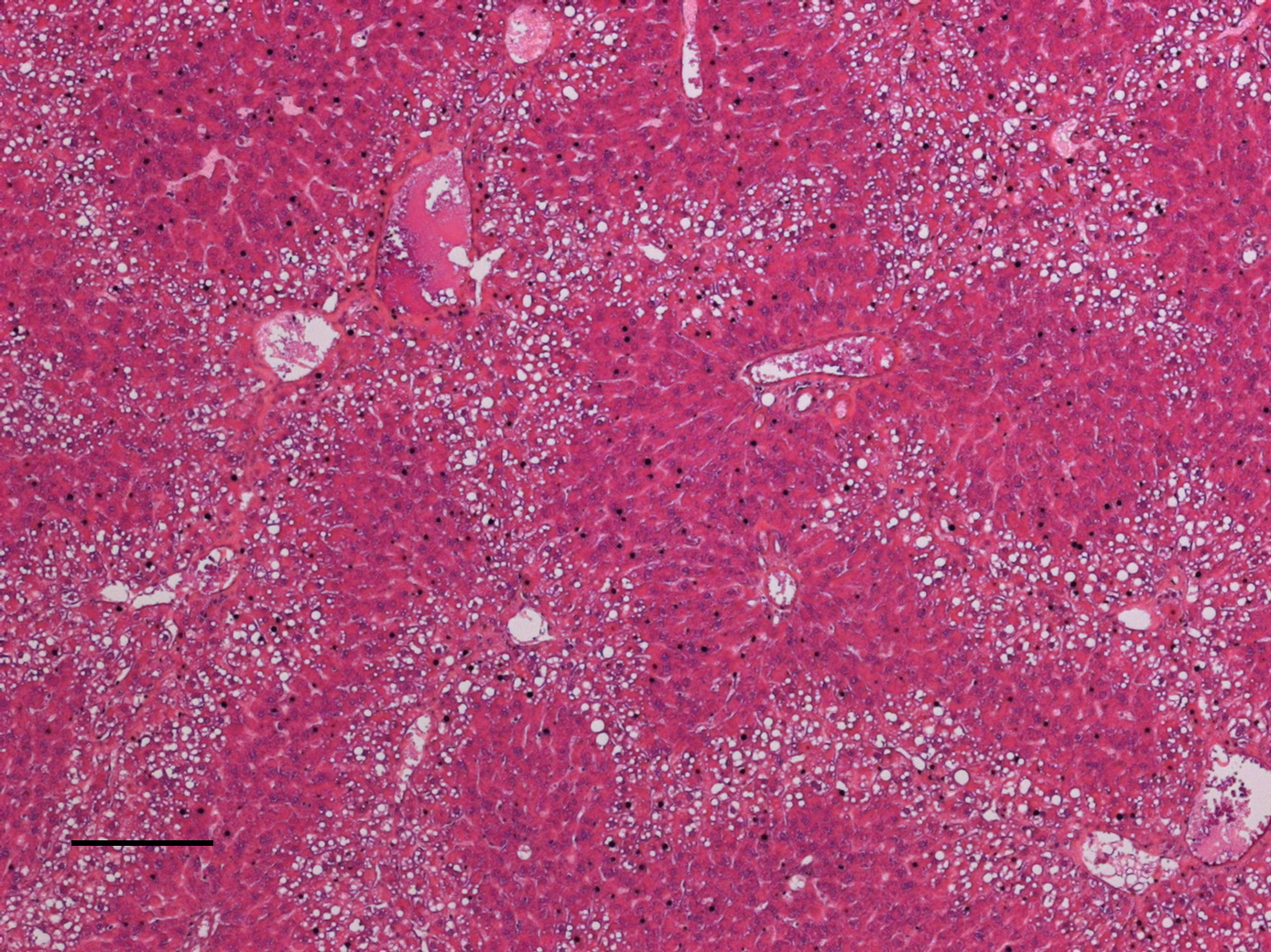

Supplement: S3 Appendix — (ZIP) [file pmed.1002248.s003.zip › CCL4 8wk he.jpg]

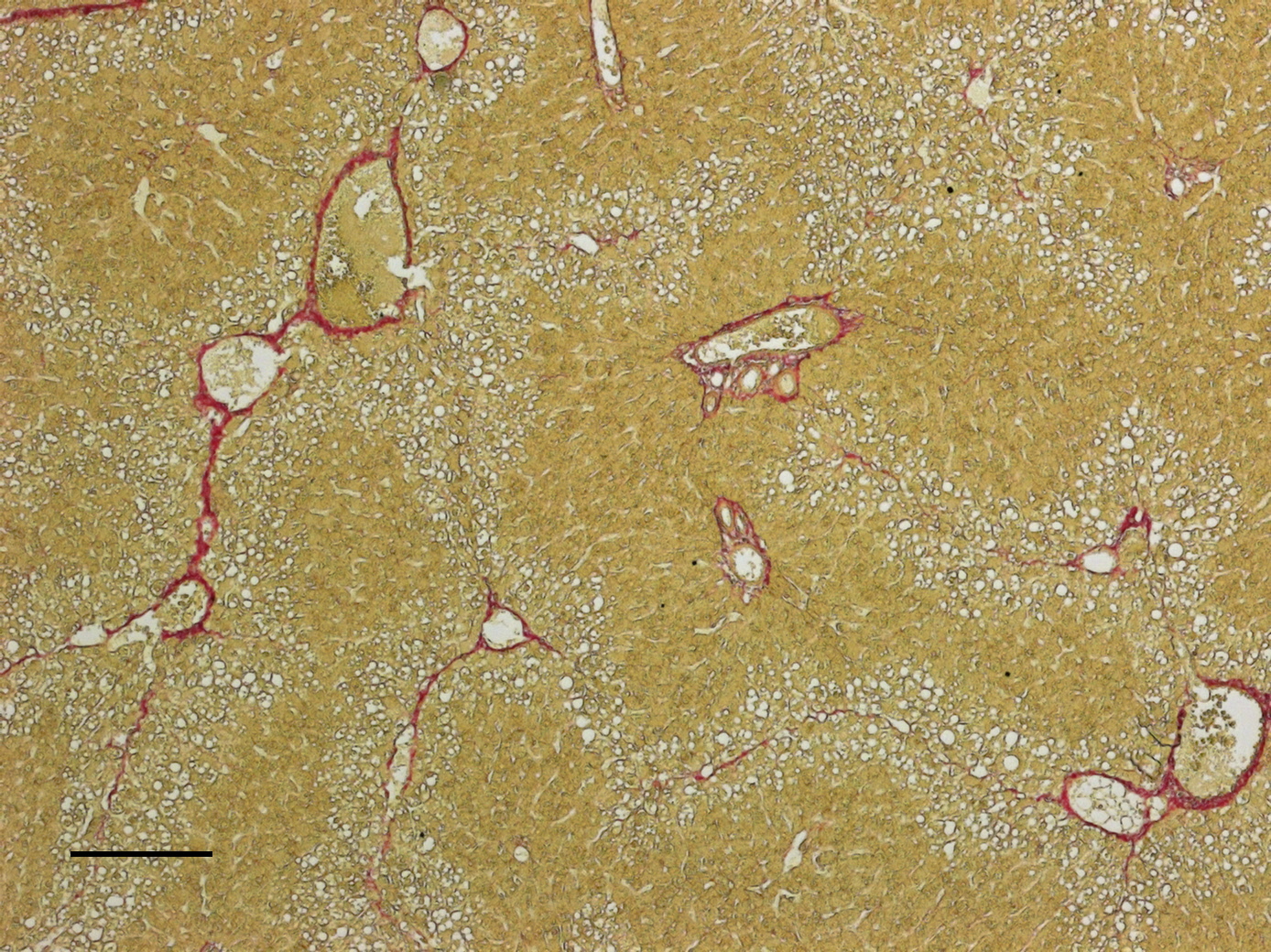

Supplement: S3 Appendix — (ZIP) [file pmed.1002248.s003.zip › CCL4 8wk psr.jpg]

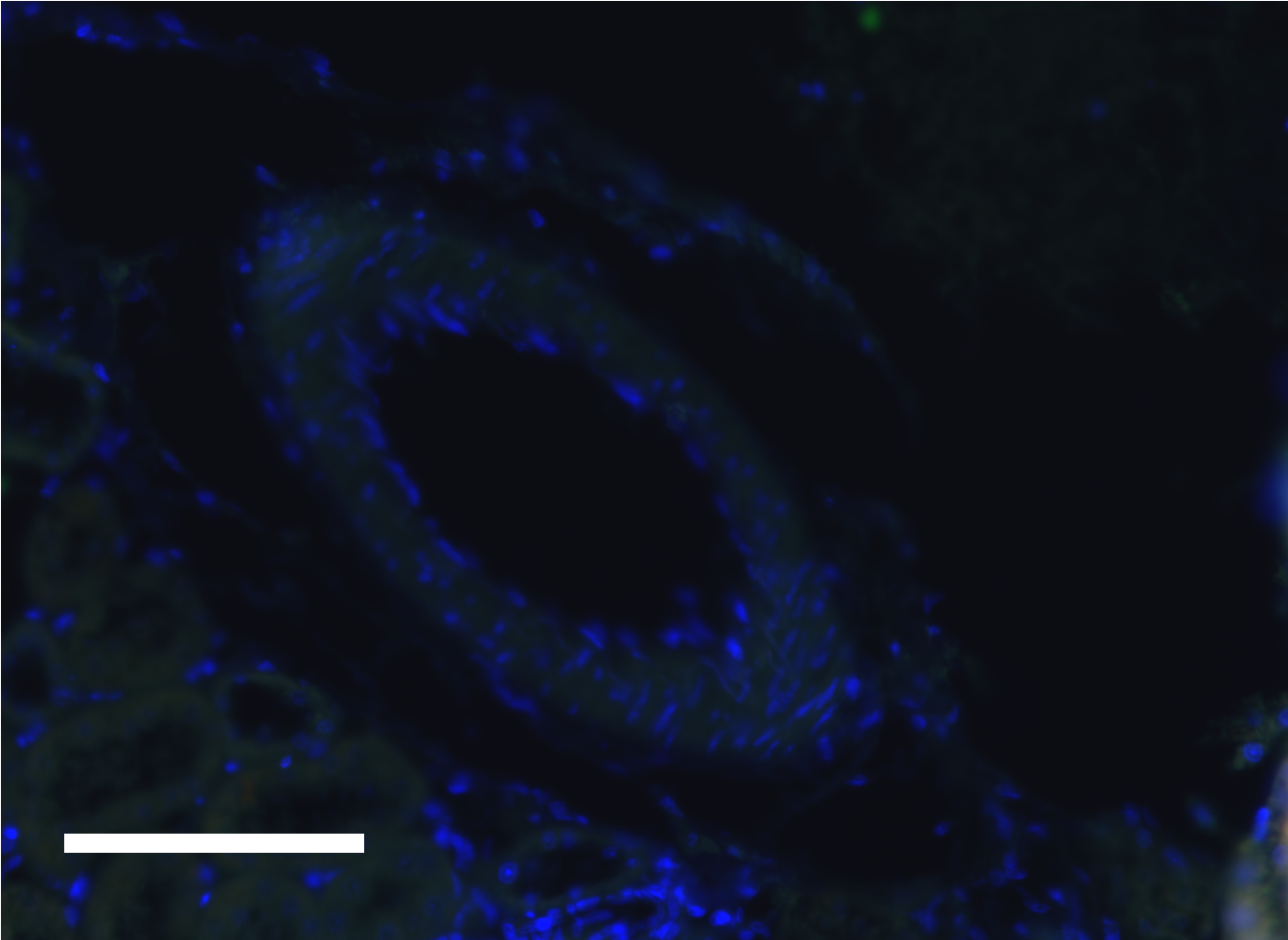

Supplement: S3 Appendix — (ZIP) [file pmed.1002248.s003.zip › Iso 4wk BDL-1.tif]

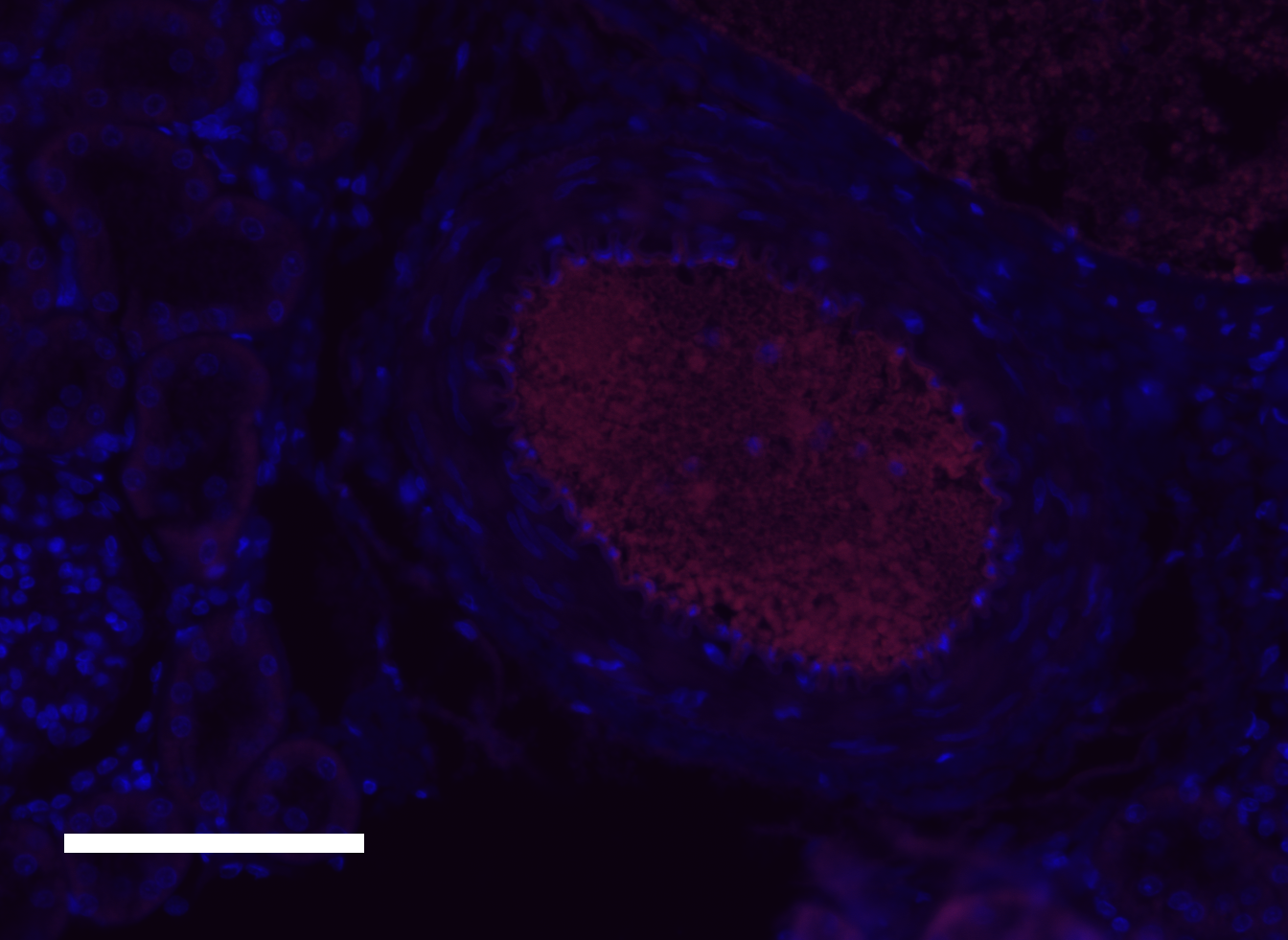

Supplement: S3 Appendix — (ZIP) [file pmed.1002248.s003.zip › Iso CCL4-16wk 5-1.tif]

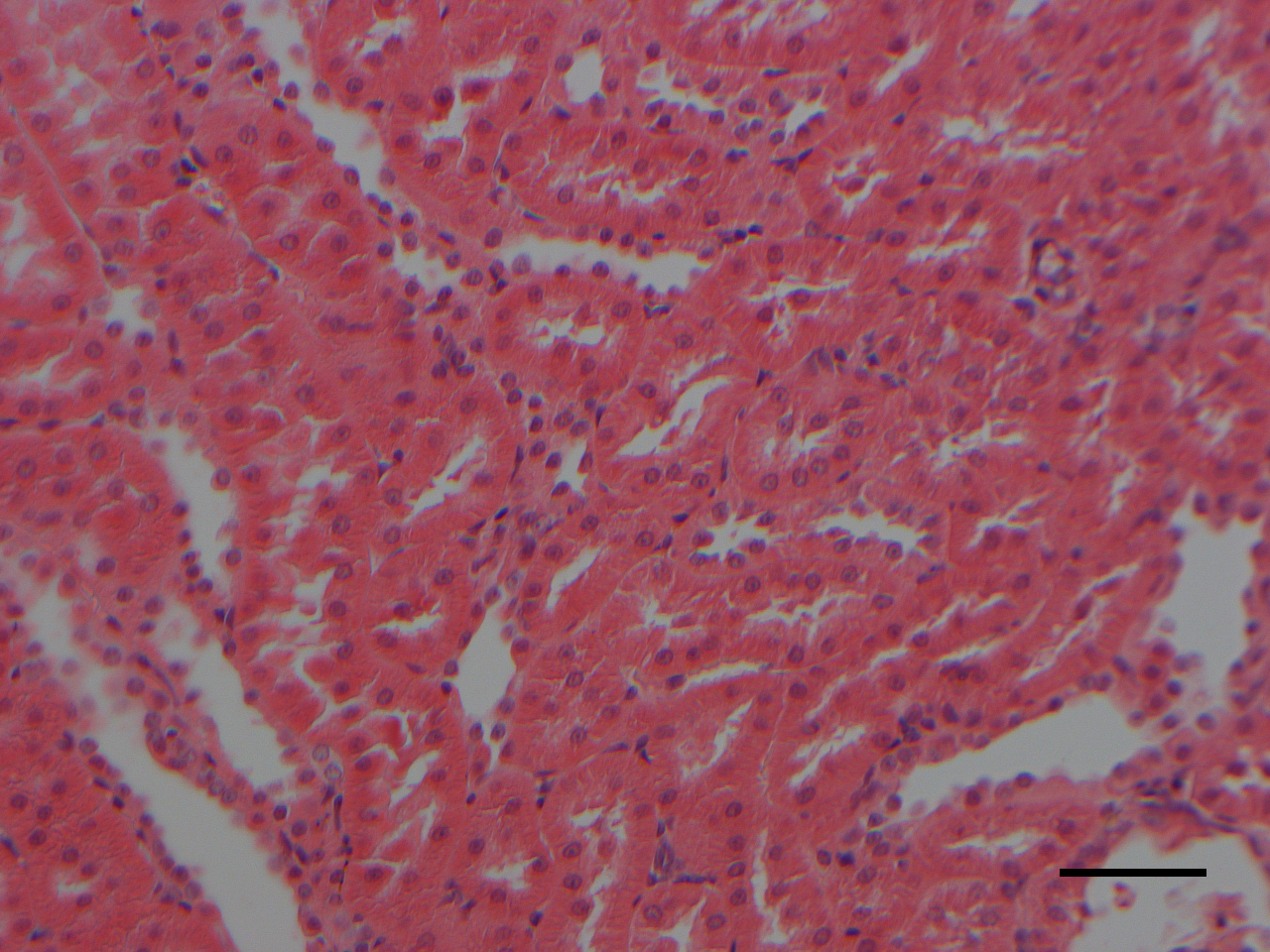

Supplement: S3 Appendix — (ZIP) [file pmed.1002248.s003.zip › Kidney H&E 12 x20 50um.tif]

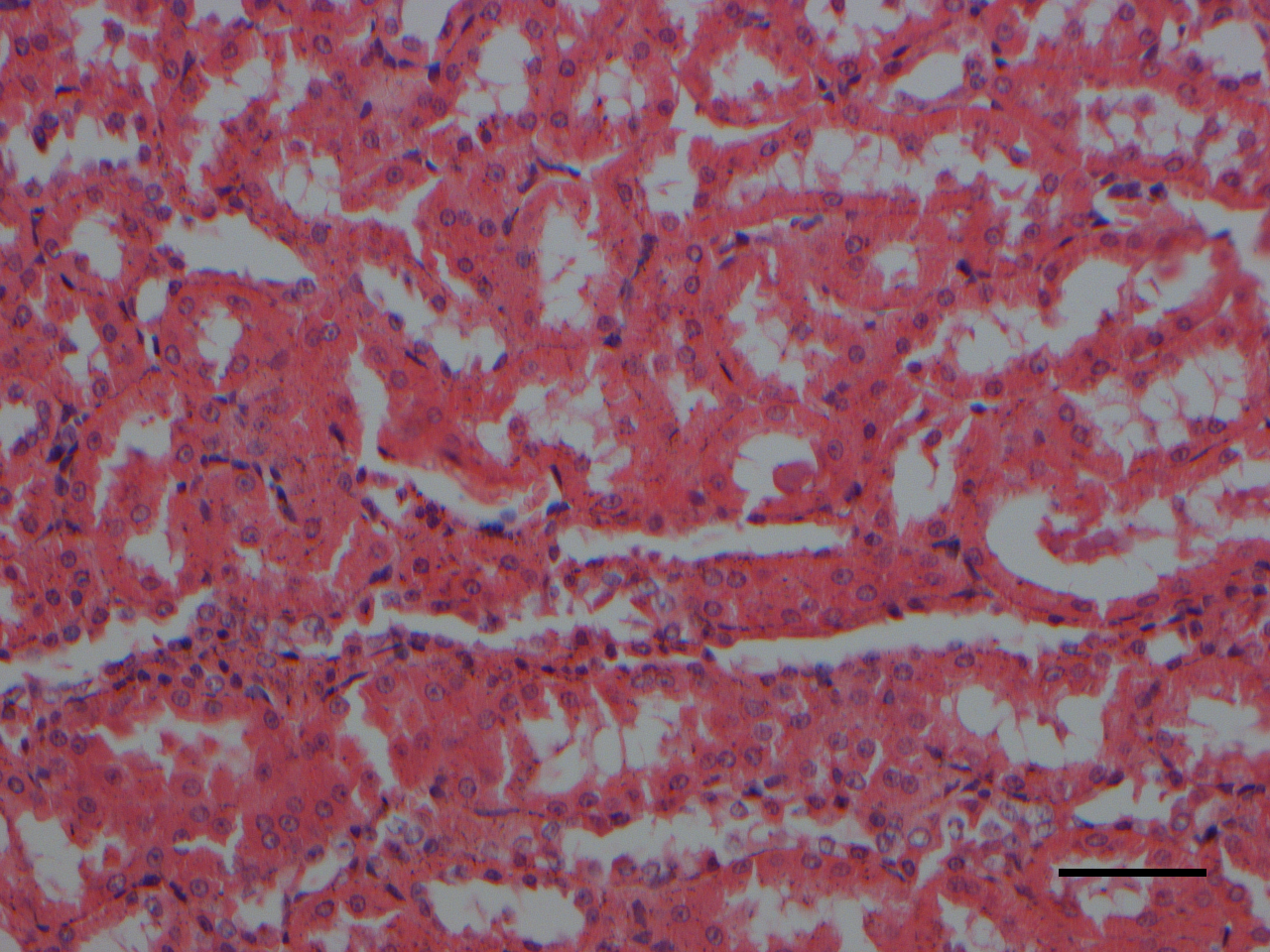

Supplement: S3 Appendix — (ZIP) [file pmed.1002248.s003.zip › Kidney H&E 24 x40b 50um.tif]

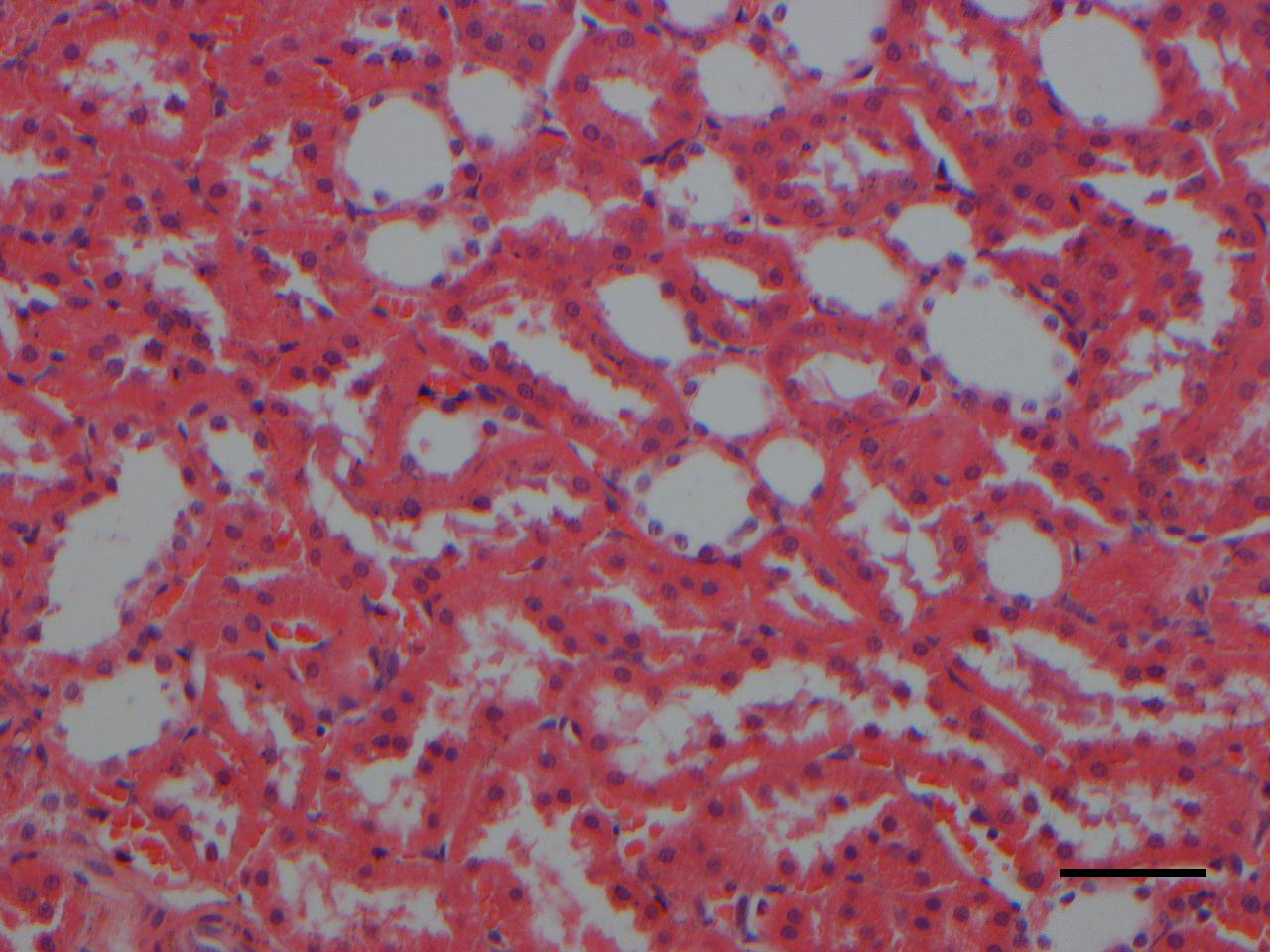

Supplement: S3 Appendix — (ZIP) [file pmed.1002248.s003.zip › Kidney H&E 26 x20 50um.tif]

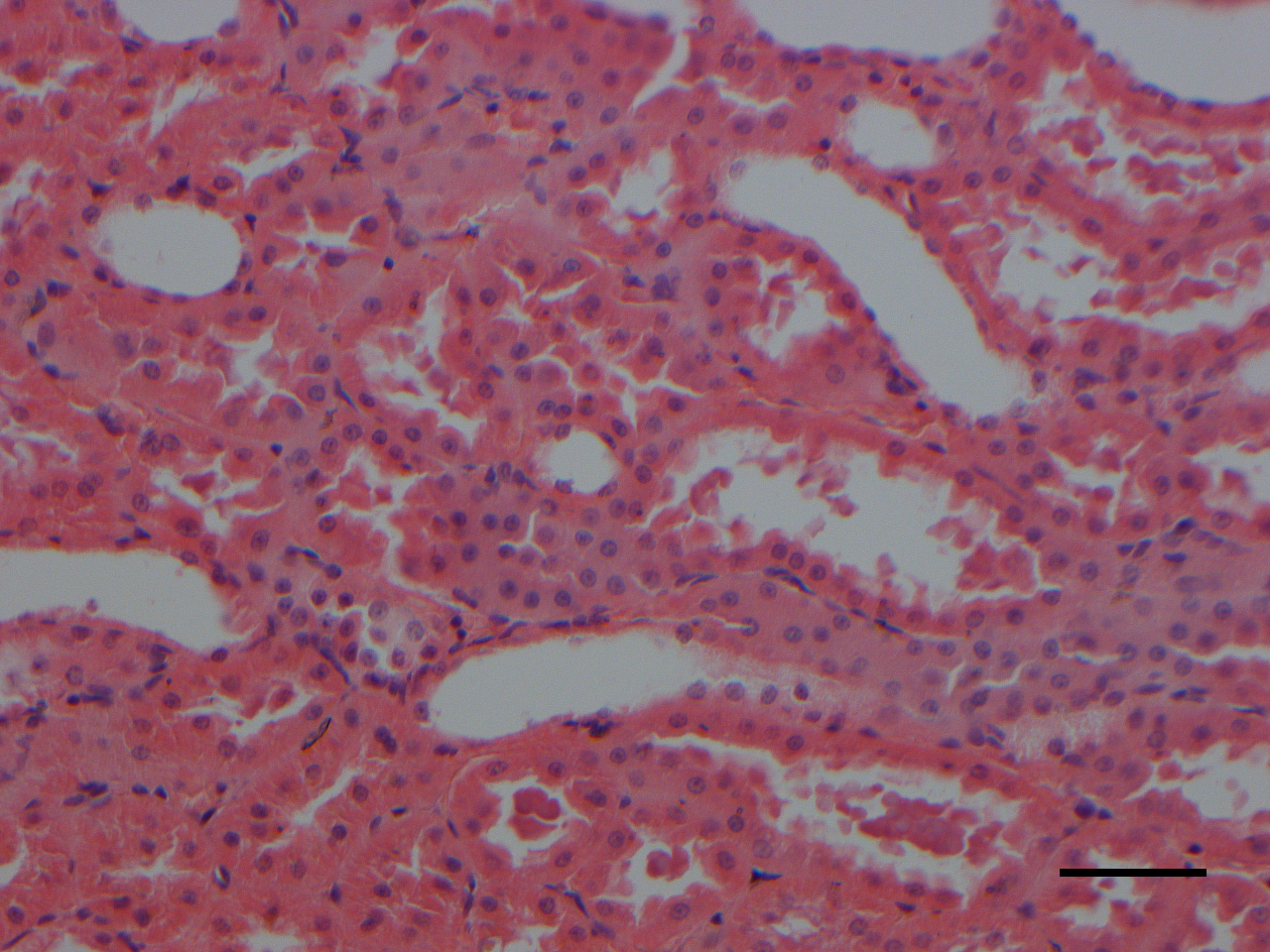

Supplement: S3 Appendix — (ZIP) [file pmed.1002248.s003.zip › Kidney H&E 7 x20 50um.tif]

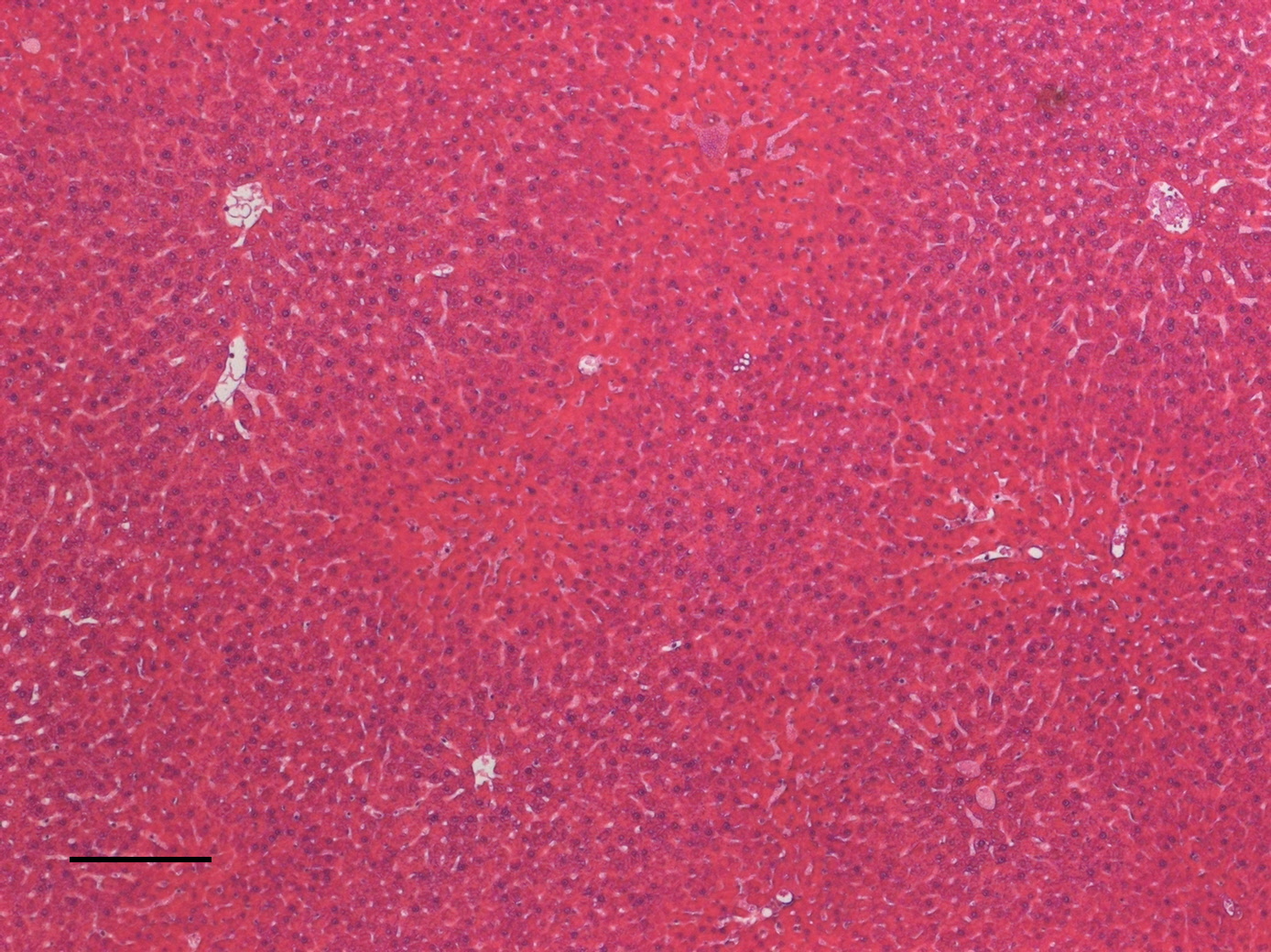

Supplement: S3 Appendix — (ZIP) [file pmed.1002248.s003.zip › oo h&e.jpg]

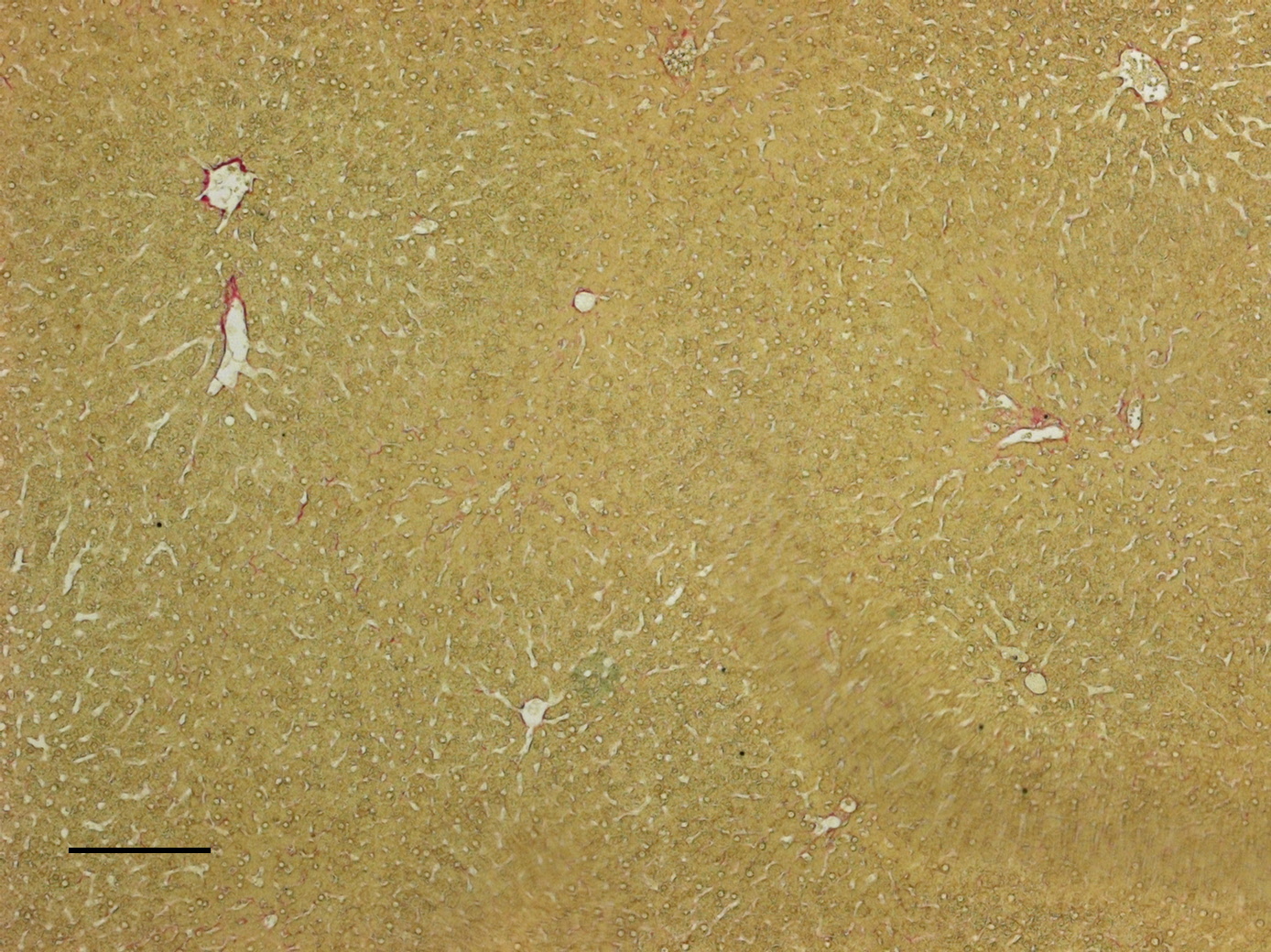

Supplement: S3 Appendix — (ZIP) [file pmed.1002248.s003.zip › oo psr.jpg]

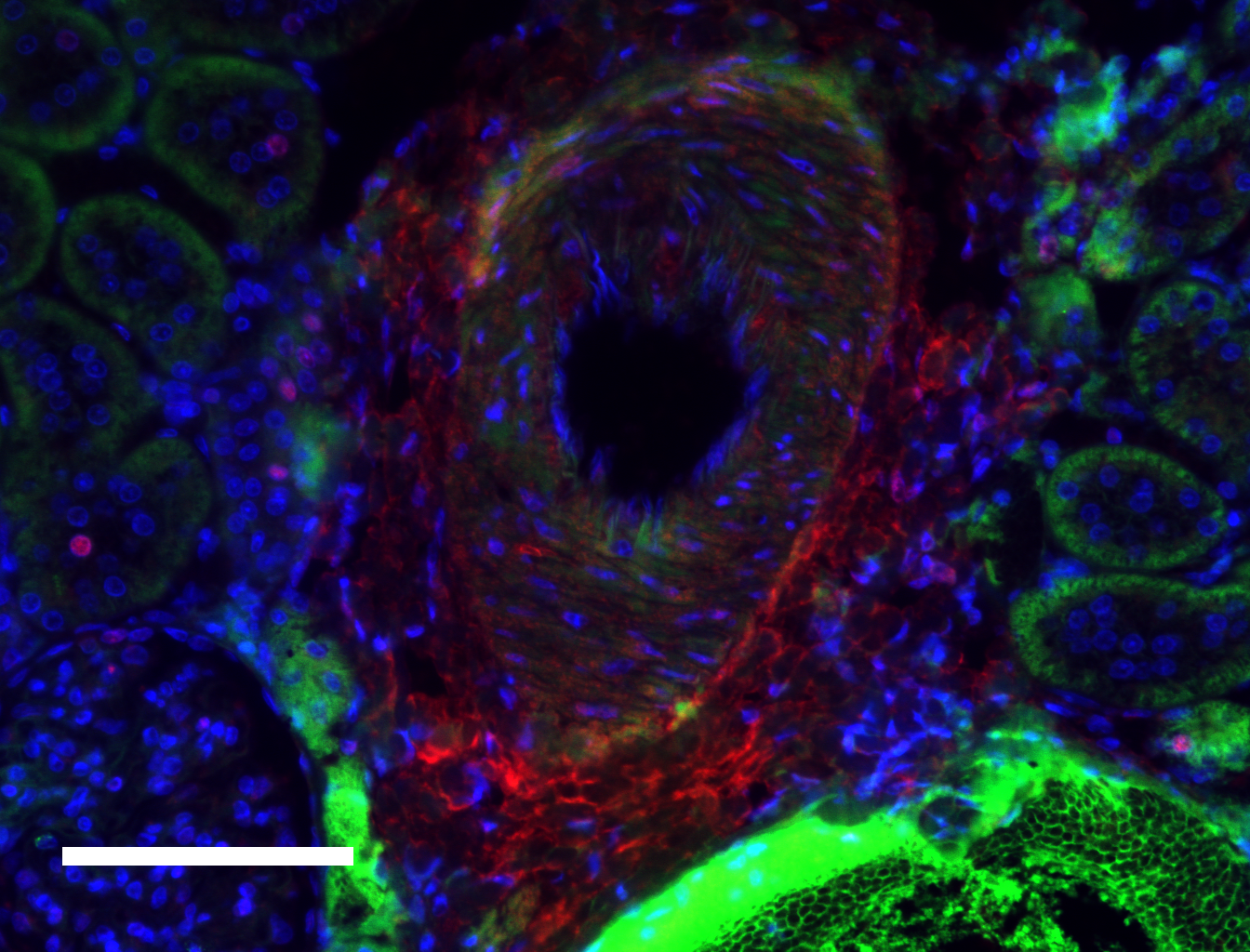

Supplement: S3 Appendix — (ZIP) [file pmed.1002248.s003.zip › RXFP1 OO 6 large vessel 9.tif (RGB).tif]

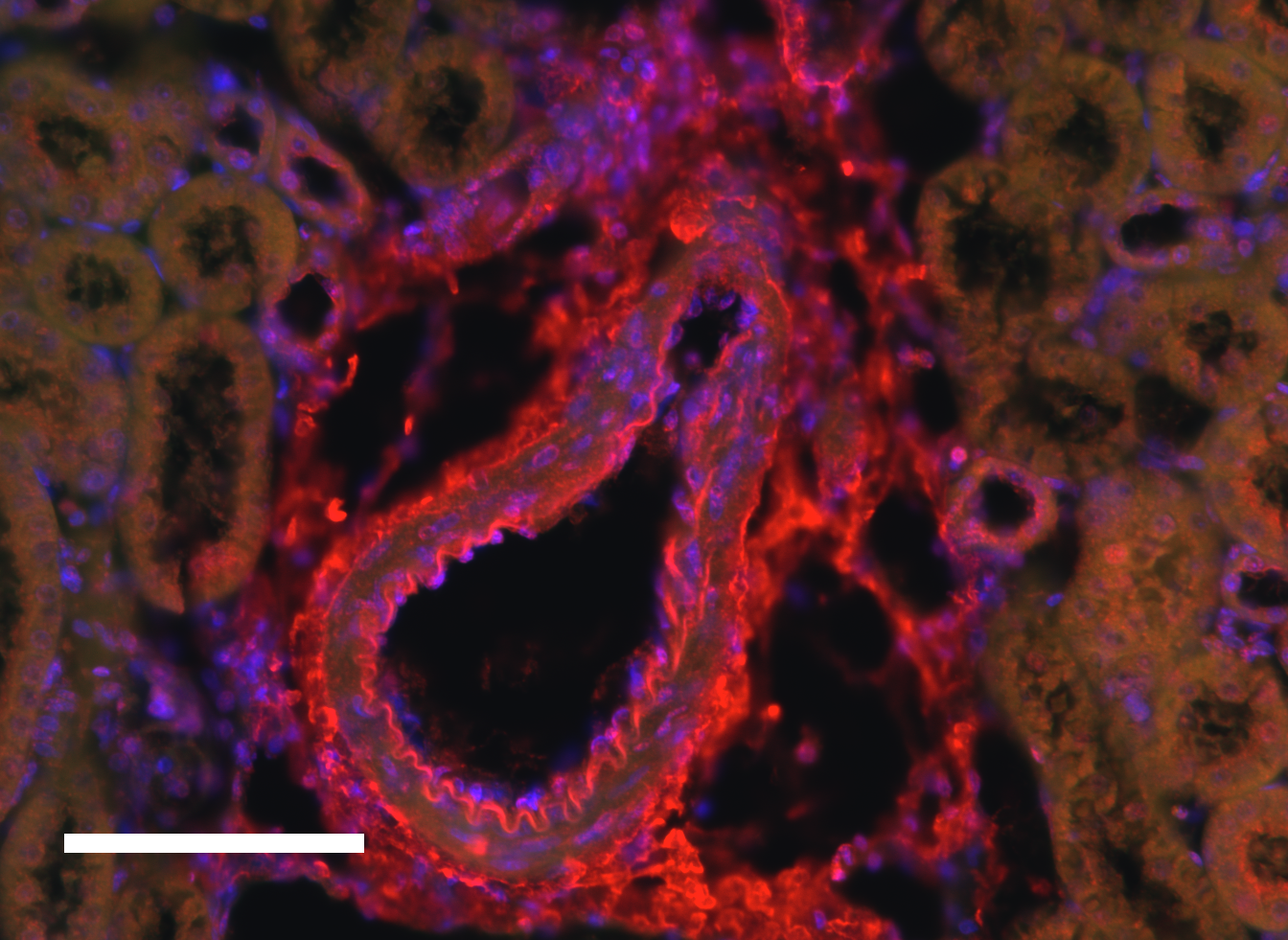

Supplement: S3 Appendix — (ZIP) [file pmed.1002248.s003.zip › RXFP1 4wk BDL 4-1.tif]

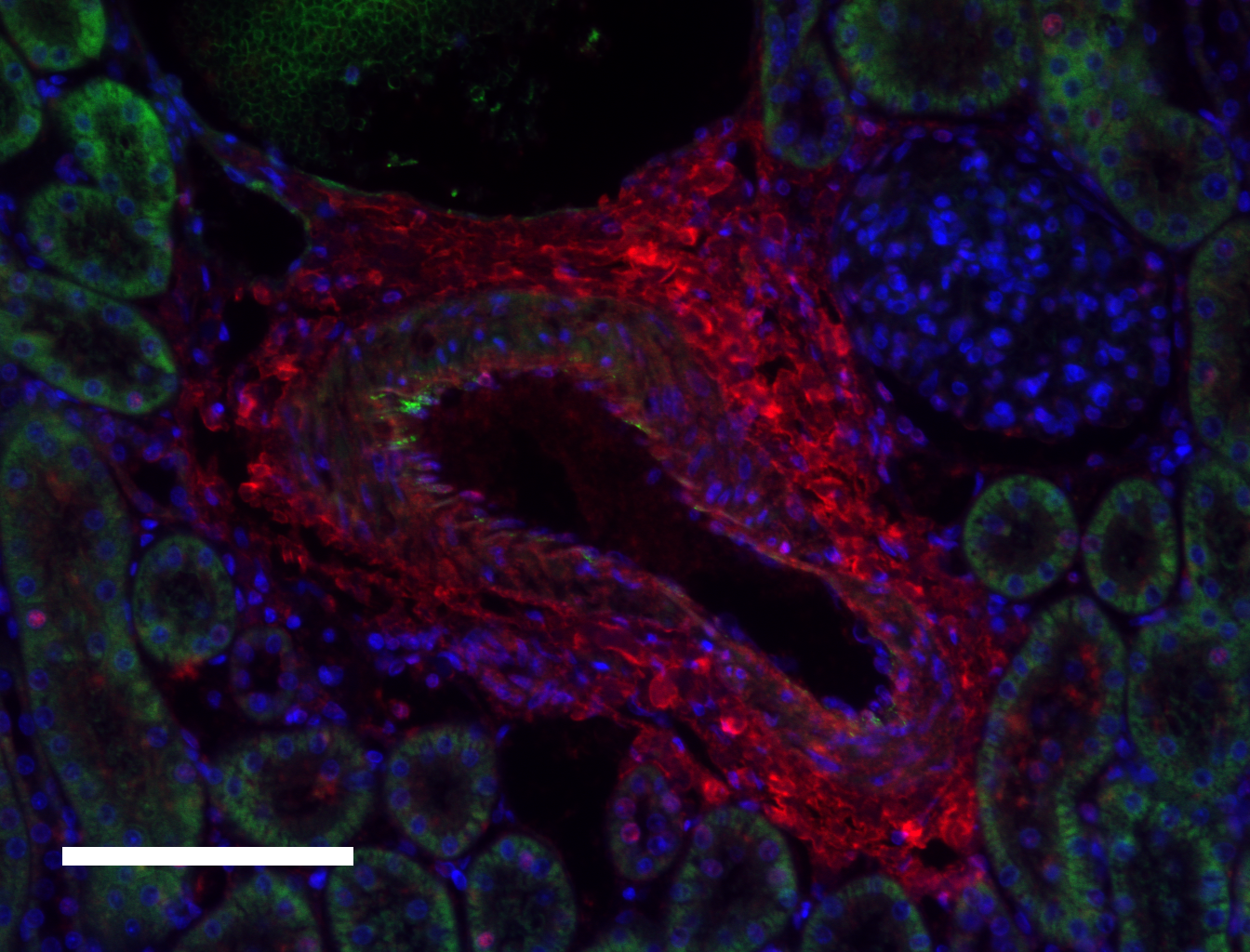

Supplement: S3 Appendix — (ZIP) [file pmed.1002248.s003.zip › RXFP1 BDL Sham large vessel 2.tif (RGB).tif]

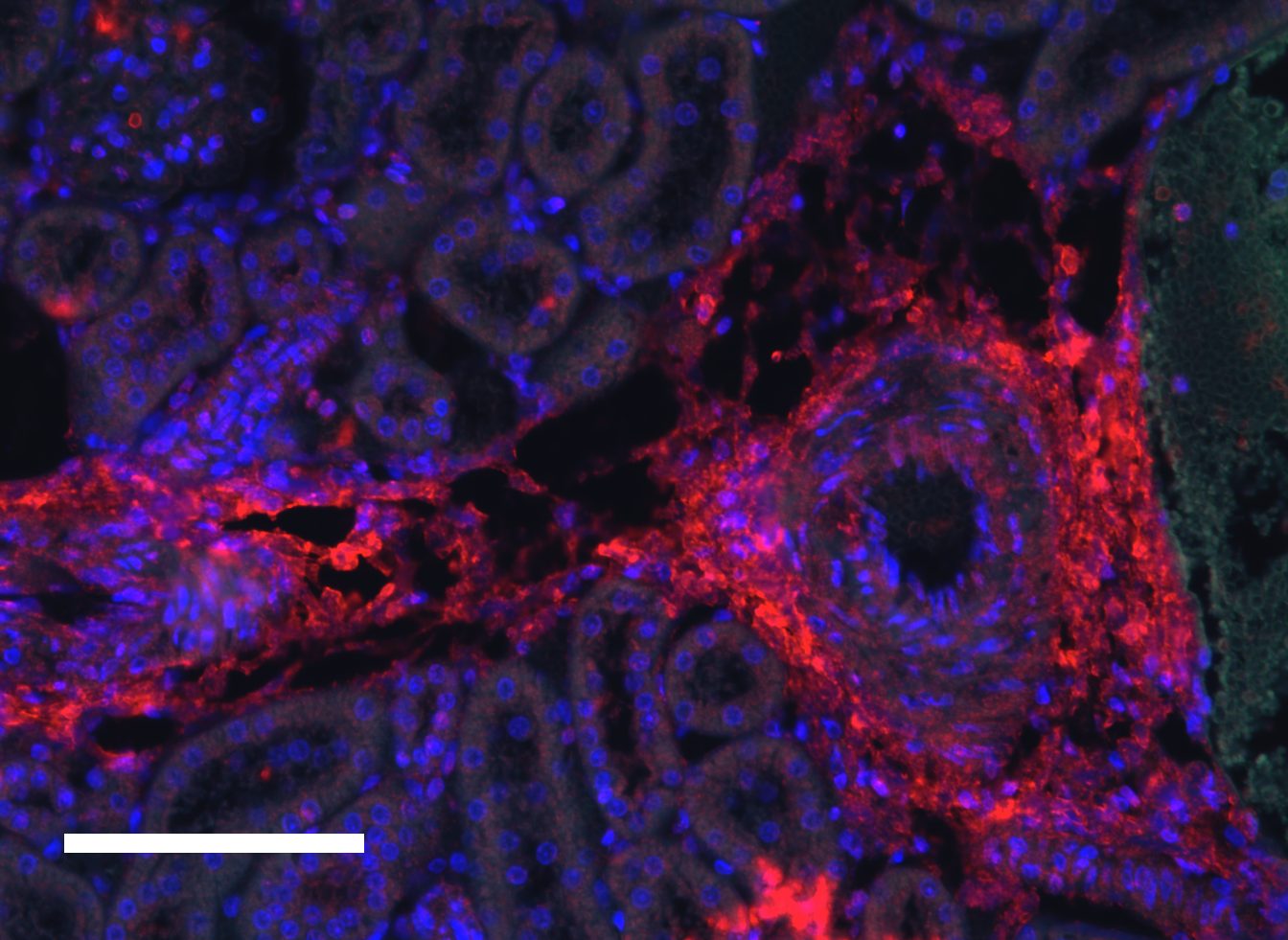

Supplement: S3 Appendix — (ZIP) [file pmed.1002248.s003.zip › RXFP1 CCL4 16wk 24-1.tif]

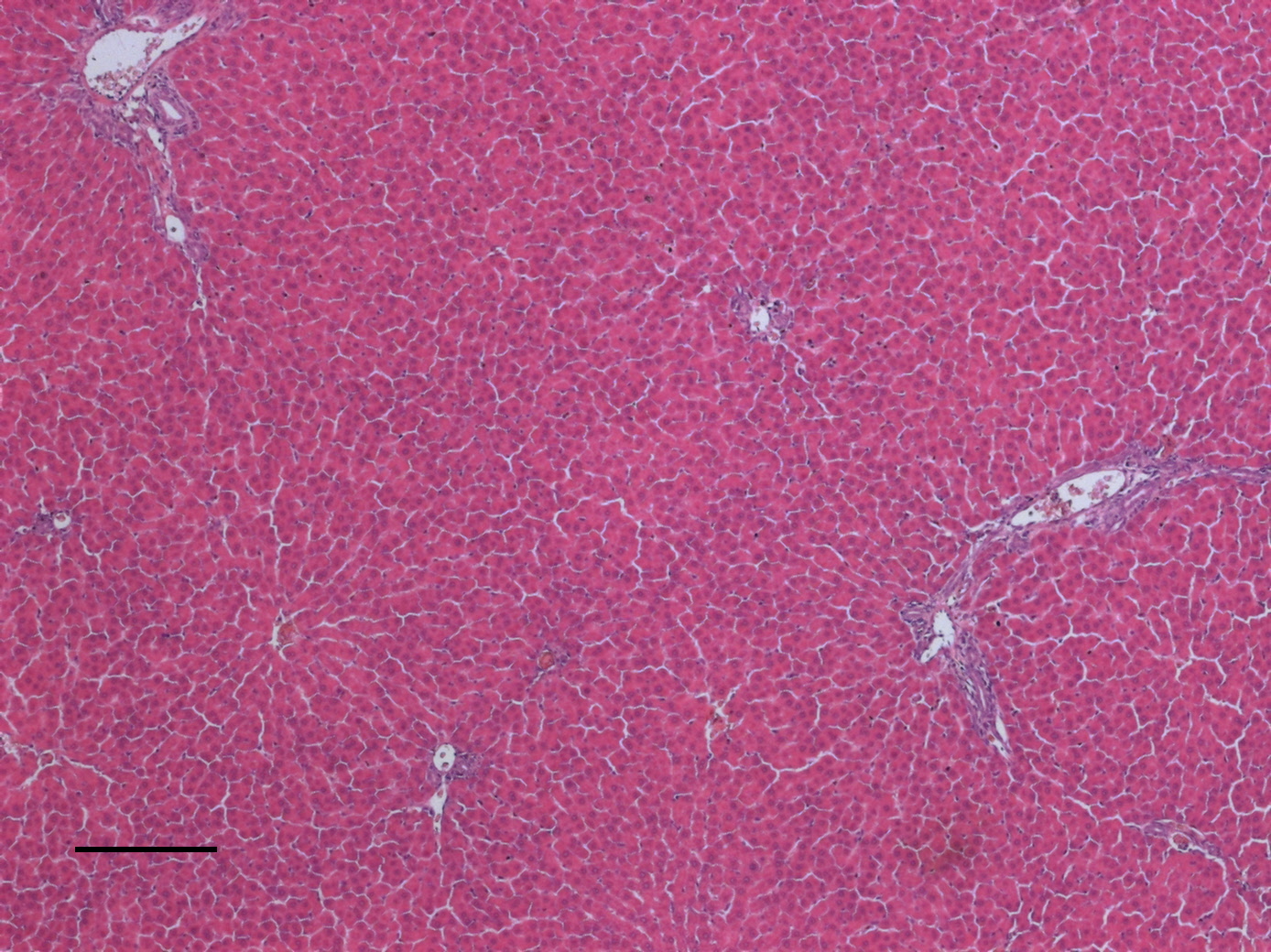

Supplement: S3 Appendix — (ZIP) [file pmed.1002248.s003.zip › sham h&e.jpg]

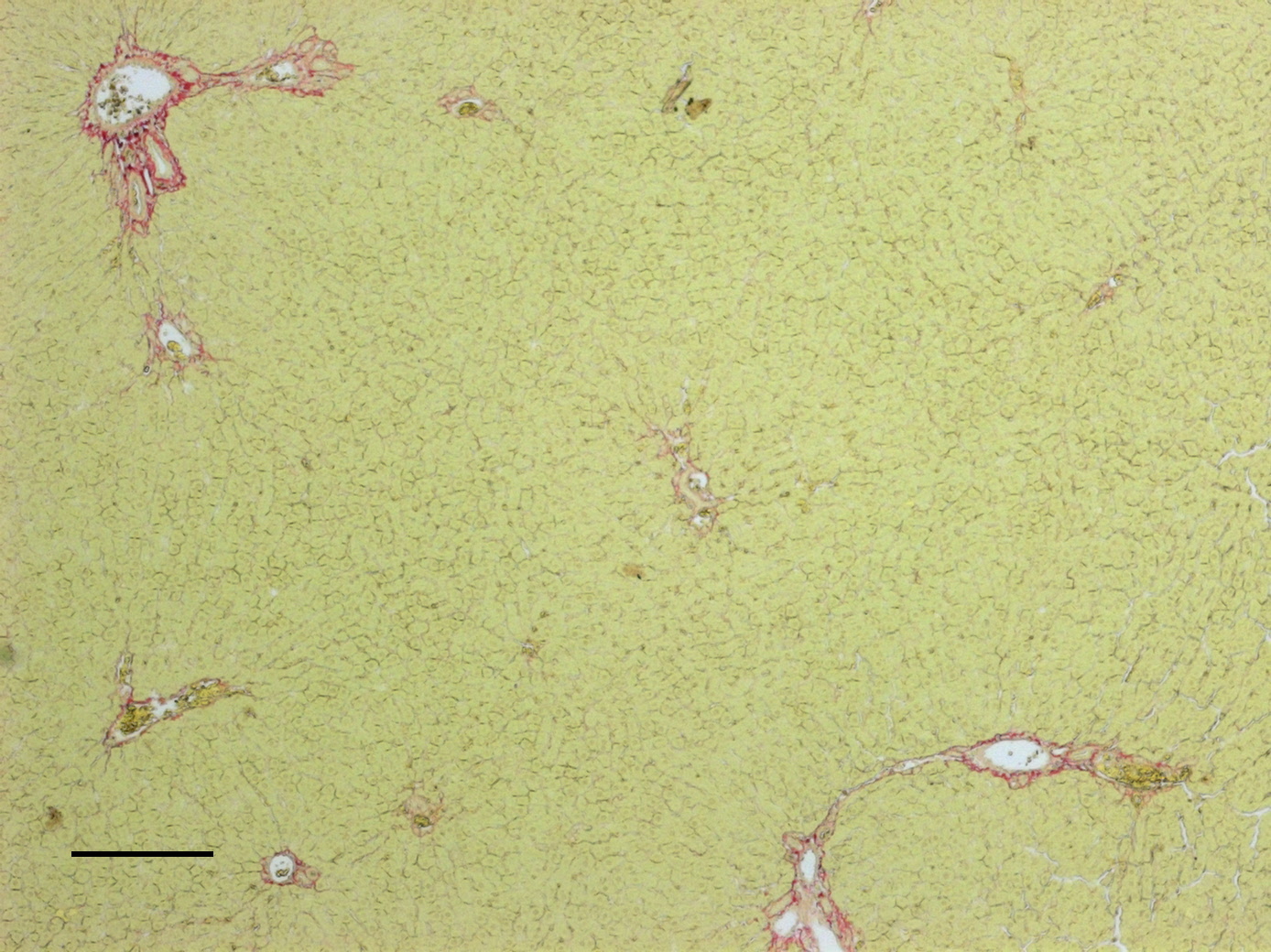

Supplement: S3 Appendix — (ZIP) [file pmed.1002248.s003.zip › sham psr.jpg]

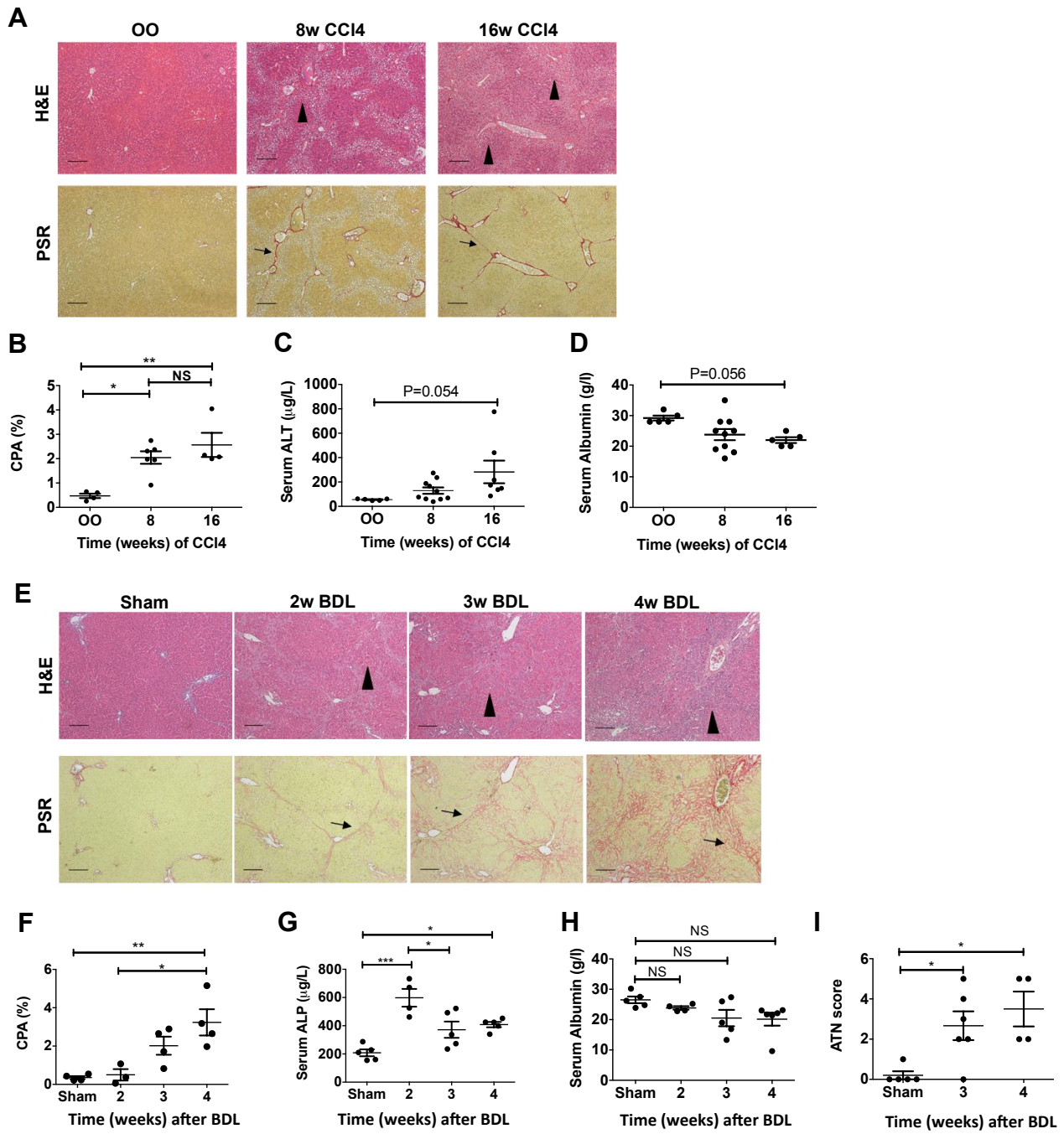

Supplement: S1 Fig — (A) Representative rat liver stained with hematoxylin and eosin (H&E) and picrosirius red (PSR) (scale bar 200 μm) in 8- and 16-wk olive oil (OO)-treated and CCl4-treated rats (n = 6–12) showing necroinflammation (arrowheads) and accumulation of fibrillar collagen (arrows). (B) Collagen proportionate area (CPA, percent) calculated by morphometric analysis of PSR staining. (C) Alanine aminotransferase (ALT) (one-way ANOVA: F[2,19] = 3.78; p = 0.042) and (D) albumin levels quantified in serum (one-way ANOVA: F[2,17] = 3.8; p = 0.043). (E) Representative H&E- and PSR-stained rat liver (scale bar 200 μm) showing necroinflammation (arrowheads) and deposition of fibrillar collagen (arrows) after bile duct ligation (BDL) (n = 5–6). (F) CPA, (G) serum alkaline phosphatase (ALP), and (H) albumin levels. (I) Acute tubular necrosis (ATN) score in BDL rat kidney. Data presented as mean ± standard error of the mean, analyzed by one-way ANOVA with post hoc Bonferroni correction (*p < 0.05; **p < 0.01; ***p < 0.001; NS, not significant). (PDF) [file pmed.1002248.s004.pdf]

**A**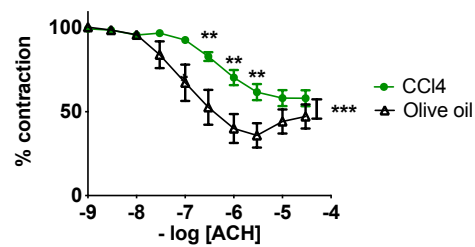**B**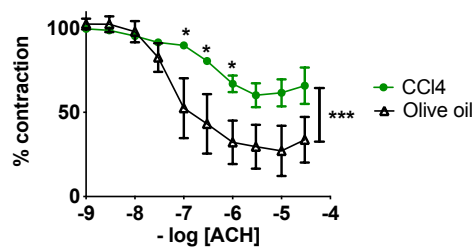**C**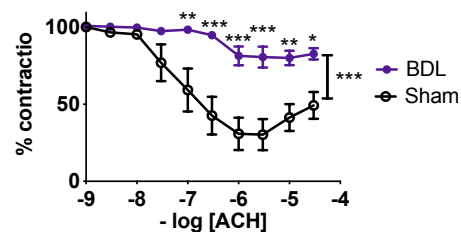**D**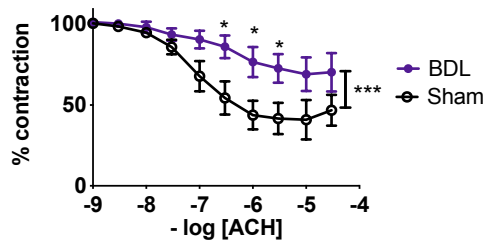**E**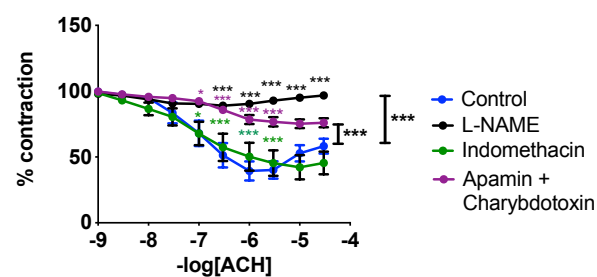**F**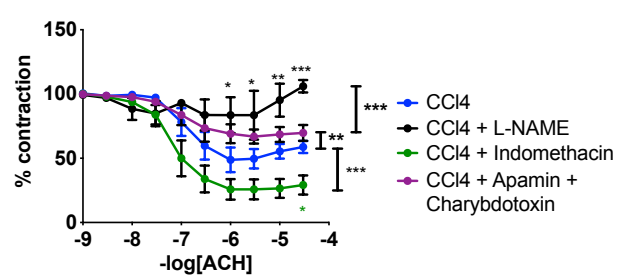

Supplement: S2 Fig — Concentration–response curves to acetylcholine (ACh; 10−9 to 10−5 M) in isolated segmental (A) and interlobar (B) renal arteries from 16-wk CCl4 and olive oil (OO) rats (n = 8–12) and in segmental (C) and interlobar (D) arteries from 4-wk bile duct ligation (BDL) and sham rats. Concentration–response curves to ACh (10−9 to 10−5 M) in extrarenal renal arteries from 16-wk OO (E) and CCl4 (F) rats co-treated with L-NG-nitroarginine methyl ester (L-NAME) (1 × 10^−4^ M), indomethacin (1 × 10^−5^ M), or apamin (1 × 10^−4^ apamin M) plus charybdotoxin (1 × 10^−5^ M). Data expressed as mean ± standard error of the mean, analyzed by two-way ANOVA with post hoc Bonferroni correction (*p < 0.05; *p < 0.01; ***p < 0.001). (PDF) [file pmed.1002248.s005.pdf]

**A**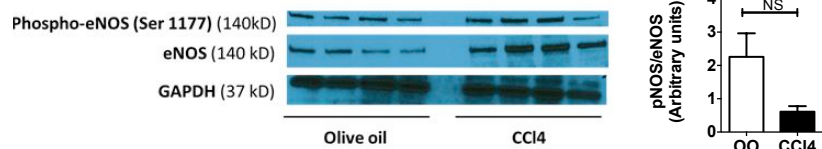**B**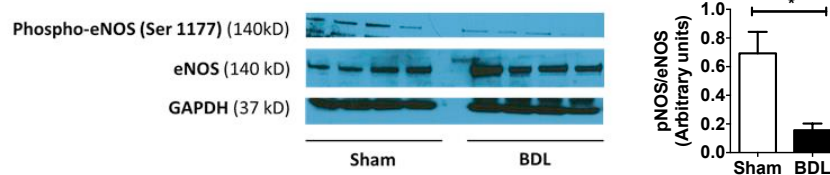**C**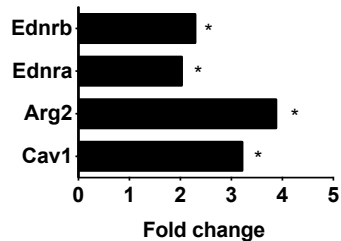**D**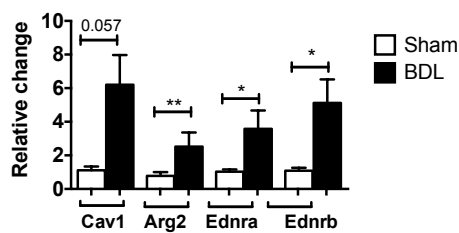**E**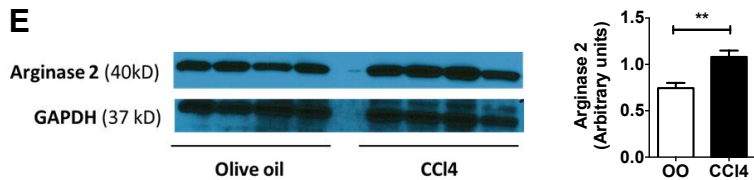**F**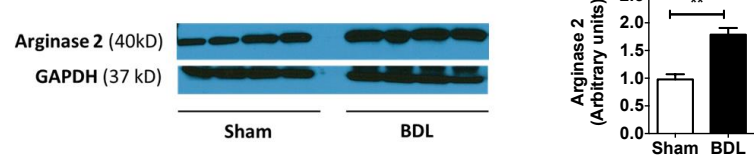**G**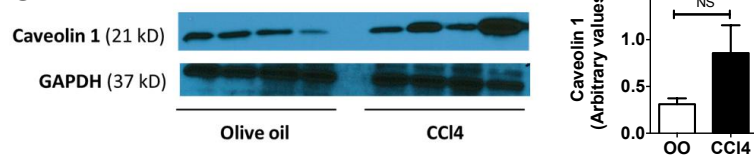**H**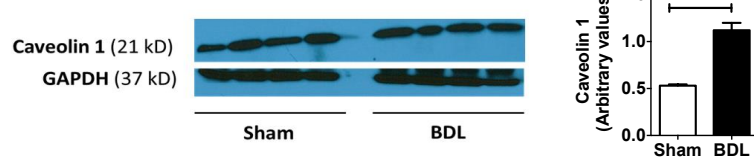

Supplement: S3 Fig — Quantification of relative p-eNOS/eNOS protein expression in whole kidney extracts from 16-wk CCl4 (A) and 4-wk bile duct ligation (BDL) (B) rat kidney (n = 4). Fold regulation of mRNA transcripts for vasoregulatory genes in kidney tissue from 16-wk CCl4 rats compared to olive oil (OO) rats (C) and BDL rats compared to sham rats (D) (n = 3–6). Genes with fold regulation >2 with p < 0.05 are presented (arginase-II, Arg2; caveolin-I, Cav1; endothelin receptor A, Ednra; endothelin receptor B, Ednrb). Quantification of arginase-II protein (E and F) and caveolin-I (G and H) relative to GAPDH in 16-wk CCl4 and 4-wk BDL rat kidney compared to controls. Data expressed as mean ± standard error of the mean, analyzed by unpaired two-tailed t-test (*p < 0.05; **p < 0.01; ***p < 0.001; NS, not significant). (PDF) [file pmed.1002248.s006.pdf]

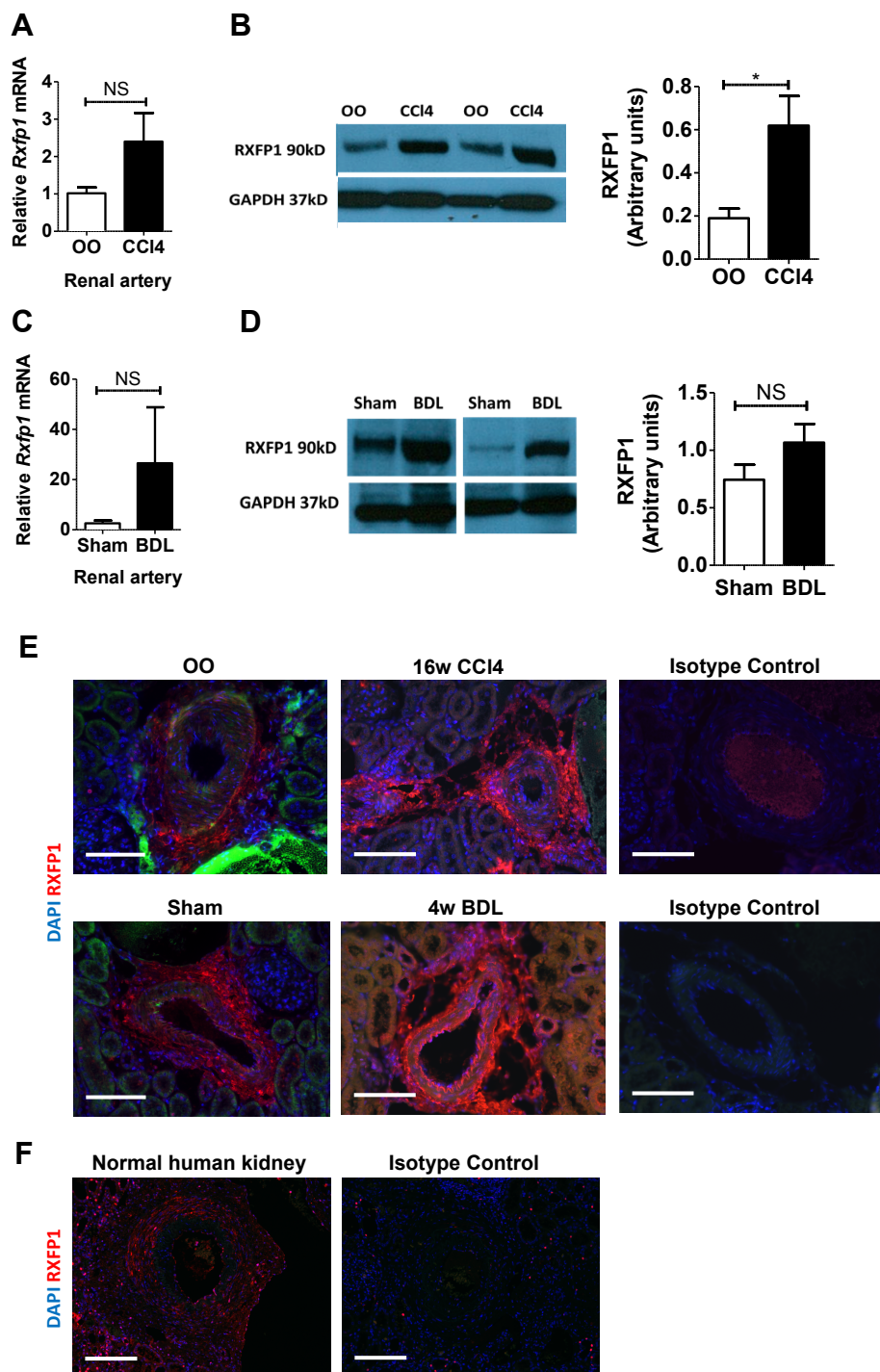

Supplement: S4 Fig — Relative Rxfp1 transcript level (normalized to 18S rRNA) in renal artery extracts from 16-wk CCl4 (A; p = 0.059) and 4-wk bile duct ligation (BDL) (C; p = 0.067) rats (n = 3–6). Quantification of RXFP1 protein relative to GAPDH in whole kidney from 16-wk olive oil (OO) and CCl4 (B) and 4-wk sham and BDL (D) rats (n = 4). Data expressed as mean ± standard error of the mean, analyzed by unpaired two-tailed t-test (*p < 0.05). Representative immunofluorescence staining for RXFP1 (and isotype control) in 16-wk OO and CCl4 kidney, in 4-wk sham and BDL kidney (E), and in biopsy tissue from normal human kidney (F). DAPI nuclear counterstain (size bars 100 μm). (PDF) [file pmed.1002248.s007.pdf]

**A**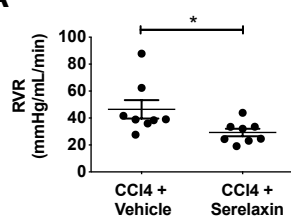**B**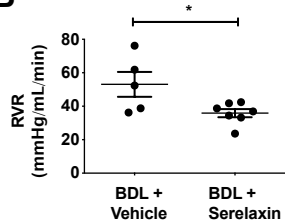**C**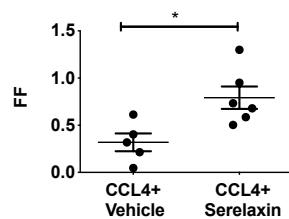**D**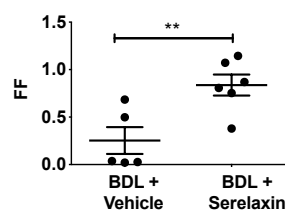

Supplement: S5 Fig — Renal vascular resistance (RVR; A and B) and filtration fraction (FF; C and D) in CCl4 and bile duct ligation (BDL) rats after 72-h subcutaneous serelaxin or vehicle (n = 5–8). Data presented as mean ± standard error of the mean, analyzed by unpaired t-test (*p < 0.05; **p < 0.01; ***p < 0.001; NS, not significant). (PDF) [file pmed.1002248.s008.pdf]

**A**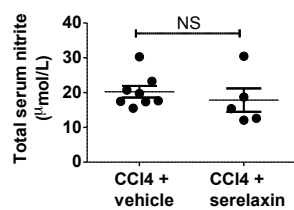**C**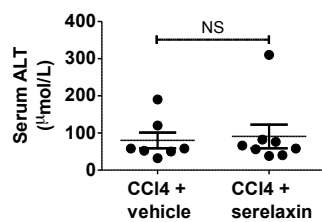**E**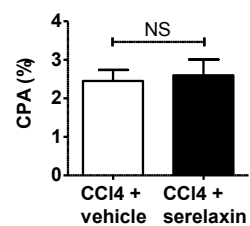**B**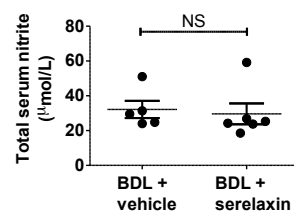**D**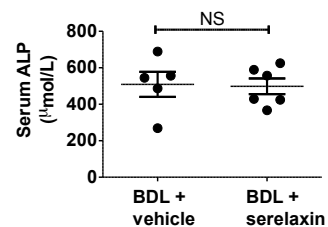**F**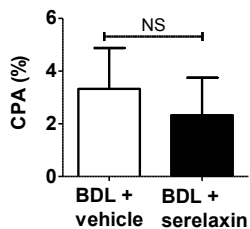

Supplement: S6 Fig — Serum total nitrite levels in 16-wk CCl4 (A) and 4-wk bile duct ligation (BDL) (B) rats after 72-h subcutaneous serelaxin or vehicle infusion. Serum alanine aminotransferase (ALT) levels in 16-wk CCl4 rats (C) and alkaline phosphatase (ALP) levels in 4-wk BDL (D) rats. Data expressed as mean ± standard error of the mean (SEM) (n = 6–8), analyzed by unpaired two-tailed t-test (NS, not significant). Collagen proportionate area (CPA, percent) measured by morphometric analysis of picrosirius-red-stained liver sections in 16-wk CCl4 (E) and 4-wk BDL (F) rats (n = 4). Data expressed as mean ± SEM, analyzed by unpaired two-tailed t-test. (PDF) [file pmed.1002248.s009.pdf]

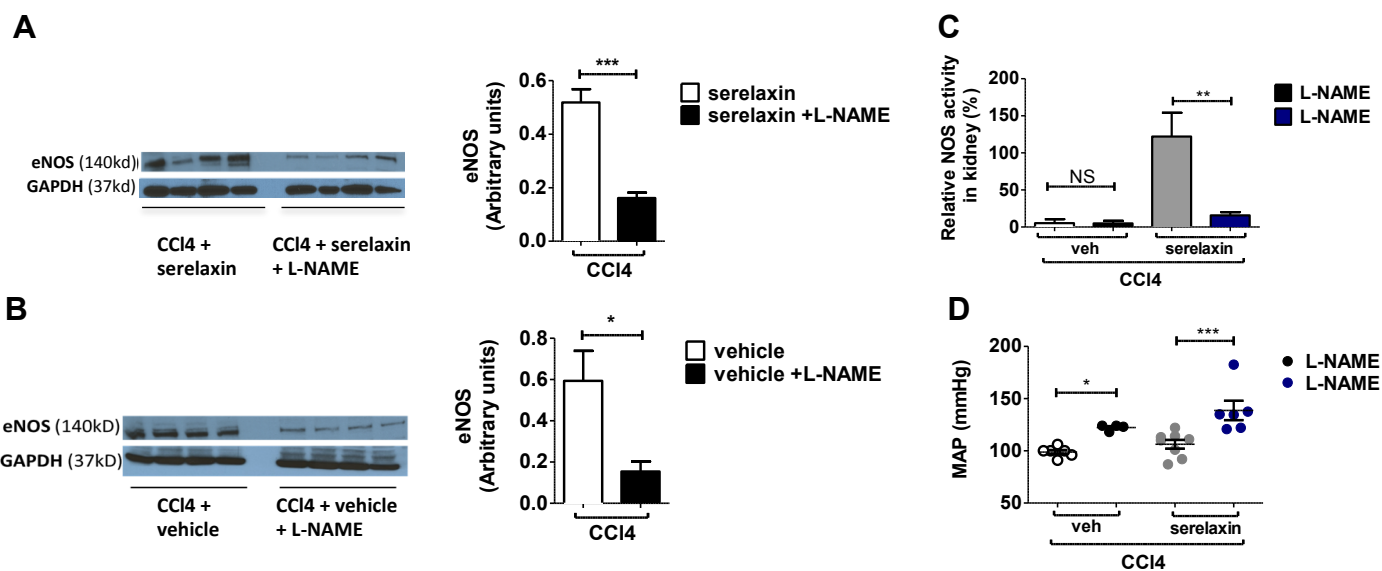

Supplement: S7 Fig — Quantification of eNOS protein (relative to GAPDH loading control) in whole kidney extracts from subgroups of 16-wk CCl4 rats randomized to treatment with serelaxin ± L-NG-nitroarginine methyl ester (L-NAME) (A) or vehicle ± L-NAME (B) (n = 4). Data expressed as mean ± standard error of the mean (SEM), analyzed by unpaired two-tailed t-test (*p < 0.05; ***p < 0.001) (C). Nitric oxide synthase (NOS) activity in whole kidney extracts (n = 4–8). Mean arterial pressure (MAP) response (D) (n = 4–8). Data expressed as mean ± SEM, analyzed by one-way ANOVA with post hoc Bonferroni correction (*p < 0.05; **p < 0.01; ***p < 0.001; NS, not significant). (PDF) [file pmed.1002248.s010.pdf]

**A**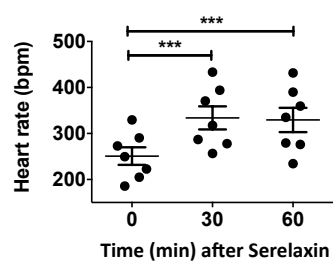**B**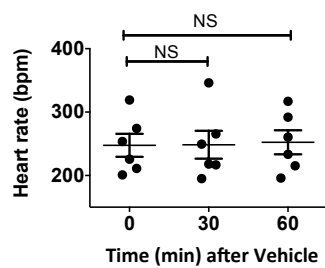**C**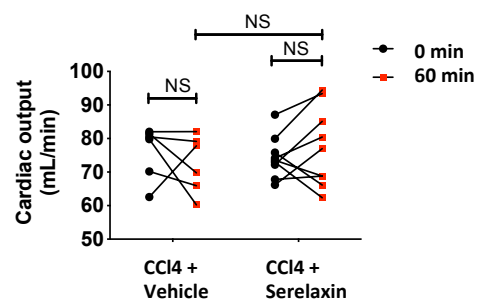**D**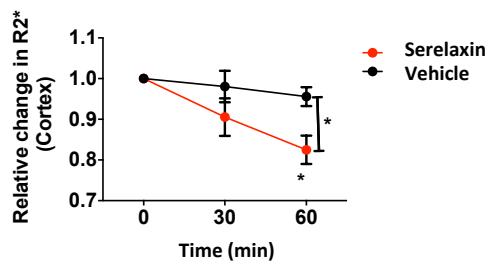

Supplement: S8 Fig — Heart rate (HR; beats per min, bpm) measured in 16-wk CCl4 rats (n = 7) randomized to intravenous (i.v.) serelaxin (4 μg in 200 μl, A) or vehicle (200 μl, B) for 60 min by femoral artery catheter. Cardiac output by M-mode Doppler (C) (n = 6–9). HR data presented as individual rats at each time point; CO data plotted at baseline and 60 min for individual rats. HR data analyzed using one-way ANOVA and CO data by two-way ANOVA, both with post hoc Bonferroni correction (***p < 0.001; NS, not significant). Deoxygenated hemoglobin levels (R2*) in renal cortex measured by blood-oxygen-level-dependent MRI in 8-wk CCl4-treated rats (n = 7) at baseline and 30 min and 60 min following i.v. serelaxin (4 μg in 200 μl) or vehicle (200 μl) (D). Data expressed as mean ± standard error of the mean, analyzed by two-way ANOVA with post hoc Bonferroni correction (*p < 0.05). (PDF) [file pmed.1002248.s011.pdf]

**A**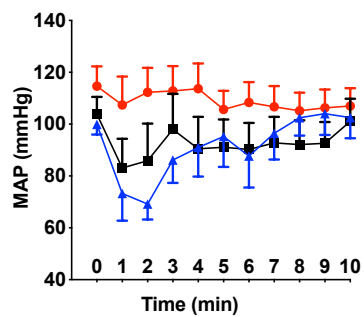**B**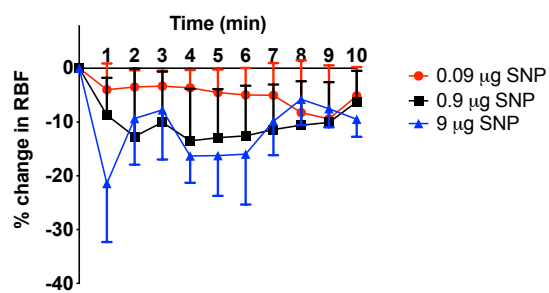**C**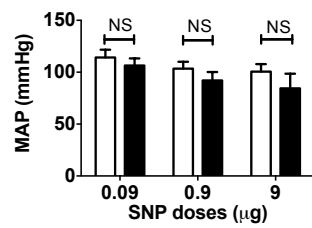**D**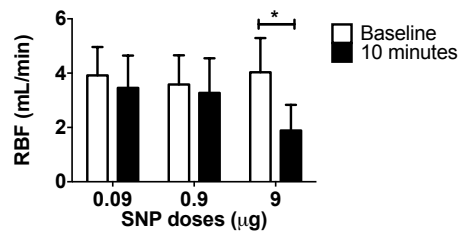

Supplement: S9 Fig — Effect of acute intravenous boluses of sodium nitroprusside (SNP; dose range 0.09–9 μg) on mean arterial pressure (MAP; A) in 16-wk CCl4 rats at serial time points, and the percentage change in renal blood flow (RBF) from baseline (B) over a 10-min observation period (n = 6). Data expressed as mean ± standard error of the mean (SEM), analyzed by one-way ANOVA with post hoc Bonferroni correction. Dose–response effect of SNP at baseline compared to 10-min MAP (C) and RBF (D). Data points represent mean ± SEM, analyzed by paired two-tailed t-test (*p < 0.05). (PDF) [file pmed.1002248.s012.pdf]

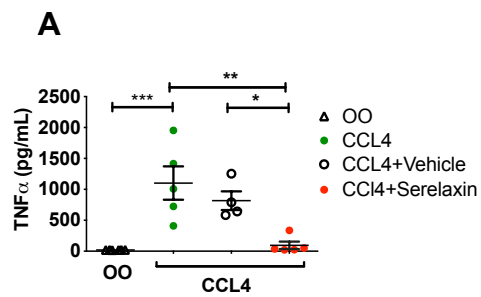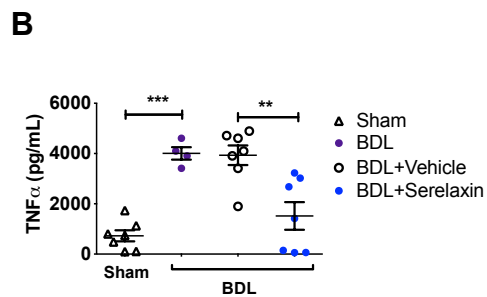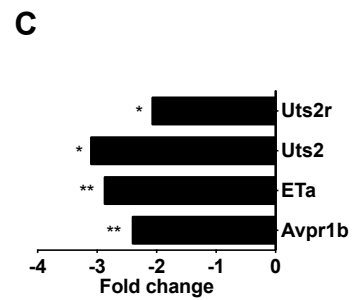

Supplement: S10 Fig — Serum TNFα levels measured by ELISA in 4-wk sham and bile duct ligation (BDL) (A) and 16-wk olive oil (OO) and CCl4 (B) rats treated for 72 h with subcutaneous serelaxin or vehicle (n = 5–8). Data presented as individual values with mean ± standard error of the mean, analyzed by one-way ANOVA with post hoc Bonferroni correction (*p < 0.05; *p < 0.01; ***p < 0.001). Fold regulation of mRNA transcripts for vasoregulatory genes in kidney tissue from 16-wk CCl4 rats compared to OO rats (n = 5) (C). Genes with a fold regulation of >2 and significance of p < 0.05 are presented. (PDF) [file pmed.1002248.s013.pdf]

A

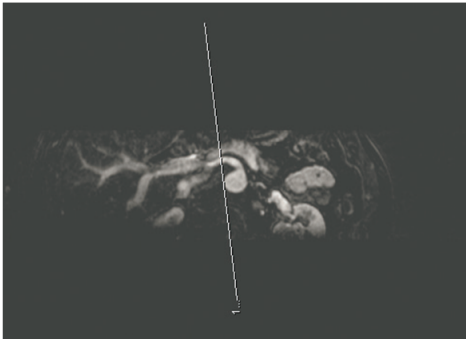

B

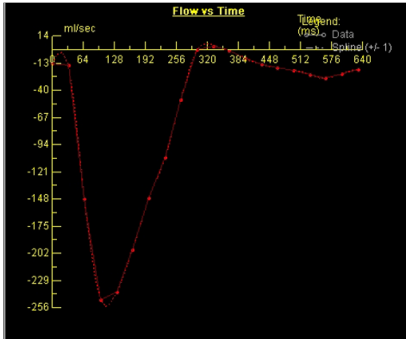

Supplement: S11 Fig — (A) Position of hepatic artery flow measurement (taken from 3-D magnetic resonance reconstruction of arterial system). (B) Phase contrast velocity-encoded images acquired from selected vessels were analyzed for peak and average flow over the cardiac cycle. (PDF) [file pmed.1002248.s014.pdf]
