# Supplementary material for: Serelaxin as a potential treatment for renal dysfunction in cirrhosis: Preclinical evaluation and results of a randomized phase 2 trial
Source: PLoS Med. 2017 Feb 28;14(2):e1002248. doi: 10.1371/journal.pmed.1002248 (PMC5330452; doi:10.1371/journal.pmed.1002248)
Supplement: S1 Table — Total renal artery flow = left + right renal artery flow; total liver flow = hepatic artery + portal vein flow. Renal vascular resistance = mean arterial pressure/total renal arterial blood flow. (DOCX) [file pmed.1002248.s015.docx]

|  | Baseline (geometric mean and 95% CI) | 120 min (geometric mean and 95% CI) | % change (geometric mean and 95% CI) | Paired *t*-test (baseline vs. 120 min) |
| --- | --- | --- | --- | --- |
| **SERELAXIN** |  |  |  |  |
| Heart Rate (beats per minute) | 71.2 (67.3, 75.4) | 75.2 (71.1, 79.6) | +5.9 (2.2, 9.6) | **P = 0.0084** |
| Systolic Blood Pressure (mmHg) | 149.1 (140.8, 157.9) | 145 (134.9, 155.8) | -2.8 (-6.3, 0.2) | P = 0.19 |
| Diastolic Blood Pressure (mmHg) | 86.5 (79.9, 93.7) | 83.8 (78, 90.1) | -3.3 (-8.8, 2.6) | P = 0.21 |
| Total Renal Artery Flow (L/min) | 0.63 (0.49,0.81) | 1.04 (0.85, 1.28) | +65.4 (40, 95) | **P = 0.000021** |
| Renal Vascular Resistance (mmHg/L/min) | 170.9 (133, 219) | 100.3 (83, 121) | -37.5 (-27,- 48) | **P=0.0003** |
| Hepatic Artery Flow (L/min) | 0.38 (0.28, 0.52) | 0.45 (0.34, 0.6) | +18.0 (-3, 44) | P = 0.11 |
| Portal Vein Flow (L/min) | 1.00 (0.86, 1.17) | 0.89 (0.73, 1.08) | -11.9 (-22, 0) | P = 0.11 |
| Total Liver Flow (L/min) | 1.45 (1.25, 1.68) | 1.44 (1.24, 1.67) | -0.5 (-8, 7) | P = 0.90 |
| Superior Mesenteric Artery Flow (L/min) | 0.45 (0.37, 0.55) | 0.45 (0.35, 0.57) | -1.5 (-18, 18) | P = 0.97 |
| Superior Abdominal Aorta Flow (L/min) | 3.42 (2.99, 3.92) | 3.69 (3.2, 4.25) | +7.8 (2, 14) | **P = 0.017** |
| Inferior Abdominal Aorta Flow (L/min) | 1.08 (0.93, 1.26) | 0.88 (0.72, 1.09) | -18.2 (-31.9, -1.2) | P = 0.42 |
| Azygos Vein Flow (L/min) | 0.20 (0.15, 0.28) | 0.19 (0.13, 0.30) | -2.9 (-32, 38) | P = 0.55 |
| **TERLIPRESSIN** |  |  |  |  |
| Heart Rate (beats per min) | 65.0 (60.7, 69.6) | 56.9 (52.8, 61.3) | -12.5 (-18.8, -5.8) | **P = 0.00003** |
| Systolic Blood Pressure (mmHg) | 144.5 (135.1, 154.5) | 153 (141.7, 165.2) | +5.9 (1.1, 12) | **P = 0.033** |
| Diastolic Blood Pressure (mmHg) | 83.7 (78.3, 89.5) | 88 (80.3, 96.4) | +5.1 (-0.5, 1) | **P = 0.049** |
| Total Renal Artery Flow (L/min) | 0.70 (0.54, 0.91) | 0.80 (0.67, 0.95) | +13.5 (3, 33) | P = 0.25 |
| Renal Vascular Resistance (mmHg/L/min) | 154 (119, 199) | 138 (119, 161) | -5 (-9, 19) | P=0.1054 |
| Hepatic Artery Flow | 0.35 (0.25, 0.49) | 0.32 (0.24, 0.43) | -7.2 (-33, 28) | P = 0.44 |
| Portal Vein Flow (L/min) | 1.19 (0.75, 1.89) | 0.72 (0.46, 1.11) | -40.0 (-57, -16) | **P = 0.01** |
| Total Liver Flow (L/min) | 1.66 (1.20, 2.30) | 1.09 (0.79, 1.49) | -34.7 (-51, -13) | **P = 0.015** |
| Superior Mesenteric Artery Flow (L/min) | 0.59 (0.49, 0.70) | 0.37 (0.29, 0.48) | -36.9 (-45, -28) | **P = 0.0000005** |
| Superior Abdominal Aorta Flow (L/min) | 4.26 (3.55, 5.12) | 3.46 (2.88, 4.16) | -18.8 (-24, -14) | **P = 0.000016** |
| Inferior Abdominal Aorta (L/min) | 1.34 (1.06, 1.69) | 0.94 (0.75, 1.18) | -25.4 (-39.16, -8.44) | **P = 0.0002** |
| Azygos Flow (L/min) | 0.25 (0.16, 0.37) | 0.17 (0.11, 0.28) | -30 (-41, -18) | **P = 0.0005** |
