# Supplementary material for: Serelaxin as a potential treatment for renal dysfunction in cirrhosis: Preclinical evaluation and results of a randomized phase 2 trial
Source: PLoS Med. 2017 Feb 28;14(2):e1002248. doi: 10.1371/journal.pmed.1002248 (PMC5330452; doi:10.1371/journal.pmed.1002248)
Supplement: S2 Table — An unpaired t-test was used to compare the difference in blood flow change from baseline induced by serelaxin and terlipressin in prespecified vessels. Mean differences, 95% CIs, and p-values are presented in the table. (DOCX) [file pmed.1002248.s016.docx]

|  | **Mean difference** | **95% CI** | ***p* value** |
| --- | --- | --- | --- |
| **Total renal artery flow** | 0.3455 ± 0.09672 | 0.1497 to 0.5413 | 0.001 |
| **Superior mesenteric artery flow** | 0.2050 ± 0.05053 | 0.1027 to 0.3073 | 0.0002 |
| **Superior abdominal aorta flow** | 1.139 ± 0.1849 | 0.7643 to 1.513 | < 0.0001 |
| **Portal vein flow** | 0.6365 ± 0.2608 | 0.1086 to 1.164 | 0.0194 |
| **Hepatic artery flow** | 0.1600 ± 0.08096 | -0.003887 to 0.3239 | 0.0554 |
