# Supplementary material for: Serelaxin as a potential treatment for renal dysfunction in cirrhosis: Preclinical evaluation and results of a randomized phase 2 trial
Source: PLoS Med. 2017 Feb 28;14(2):e1002248. doi: 10.1371/journal.pmed.1002248 (PMC5330452; doi:10.1371/journal.pmed.1002248)
Supplement: S1 Text — (PDF) [file pmed.1002248.s017.pdf]

NOVARTIS

Clinical Development

RLX030/Serelaxin

Clinical Trial Protocol CRLX030X2201

An exploratory study to investigate the haemodynamic effects of serelaxin in patients with compensated cirrhosis and portal hypertension

Authors: Protocol developed by Novartis Pharma AG [redacted]

Document type: Amended Clinical Trial Protocol

EUDRACT number: 2012-000236-26

Version number: v01 clean

Development phase: II

Release date: 10-Oct-2014

[redacted]

Template version 21-SEP-2011

PPD [redacted]

Table of contents

Table of contents ... 2
List of tables ... 5
List of figures ... 5
List of abbreviations ... 6
Glossary of terms ... 8
Protocol synopsis ... 9
Amendment 1 ... 10
1 Introduction ... 11
1.1 Background ... 11
1.2 Purpose ... 13
2 Study objectives ... 13
2.1 Primary objectives ... 13
2.2 Secondary objectives ... 13
2.3 Exploratory objectives ... 14
3 Investigational plan ... 14
3.1 Study design ... 14
3.2 Rationale of study design ... 17
3.3 Rationale of dose/regimen, duration of treatment ... 17
3.4 Rationale for choice of comparator ... 18
3.5 Purpose and timing of interim analyses/design adaptations ... 19
3.6 Risks and benefits ... 19
4 Population ... 21
4.1 Inclusion criteria ... 21
4.2 Exclusion criteria ... 22
5 Treatment ... 24
5.1 Investigational and control treatment ... 24
5.2 Treatment arms ... 24
5.3 Treatment assignment ... 24
5.4 Treatment blinding ... 25
5.5 Treating the patient ... 25
5.5.1 Patient numbering ... 25
5.5.2 Dispensing the study treatment ... 25
5.5.3 Supply, storage and tracking of study treatment ... 25
5.5.4 Instructions for administering study treatment ... 26
5.5.5 Permitted dose adjustments and interruptions of study treatment ... 26

5.5.6 Rescue medication 26
5.5.7 Concomitant treatment 26
5.5.8 Prohibited treatment 27
5.5.9 Discontinuation of study treatment and premature patient withdrawal 27
5.5.10 Emergency unblinding of treatment assignment 28
5.5.11 Study completion and post-study treatment 28
5.5.12 Early study termination 28
6 Visit schedule and assessments 28
6.1 Information to be collected on screening failures 31
6.2 Patient demographics/other baseline characteristics 31
6.3 Treatment exposure and compliance 31
6.4 Efficacy 31
6.4.1 Part A: Blood flow measured via MRA 31
6.4.2 Part B: Direct venous pressure measurement 32
6.4.3 Appropriateness of efficacy assessments 33
6.5 Safety 34
6.5.1 Physical examination 34
6.5.2 Vital signs 34
6.5.3 Local ECG 34
6.5.4 Central ECG 35
6.5.5 Laboratory safety evaluations 35
6.5.6 Appropriateness of safety measurements 36
6.6 Other assessments 36
6.6.1 Biomarkers 36
6.6.2 Immunogenicity (anti-serelaxin antibodies and neutralizing activity) 36
6.6.3 Pharmacokinetics 37
7 Safety monitoring 38
7.1 Adverse events 38
7.2 Serious adverse event reporting 39
7.3 Liver safety monitoring 40
7.4 Pregnancy reporting 41
7.5 Data Monitoring Committee 41
8 Data review and database management 41
8.1 Site monitoring 41
8.2 Data collection 42

PPD [redacted]

8.3 Database management and quality control 42
9 Data analysis 43
9.1 Analysis sets 43
9.2 Patient demographics and other baseline characteristics 44
9.2.1 Demographics and baseline characteristics 44
9.2.2 Medical history 44
9.3 Treatments (study drug, rescue medication, other concomitant therapies, compliance) 44
9.3.1 Study treatment 44
9.3.2 Prior and concomitant treatment 45
9.4 Analysis of the primary variable(s) 45
9.4.1 Variable 46
9.4.2 Statistical model, hypothesis, and method of analysis 46
9.4.3 Handling of missing values/censoring/discontinuations 46
9.4.4 Supportive analyses 46
9.5 Analysis of secondary variables 47
9.5.1 Key secondary variables 47
9.5.2 Efficacy variables 47
9.5.3 Safety variables 48
9.5.4 Resource utilization 50
9.5.5 Health-related Quality of Life 50
9.5.6 Pharmacokinetics 50
9.5.7 Pharmacogenetics/pharmacogenomics 51
9.5.8 Biomarkers 51
9.5.9 Immunogenicity 51
9.6 Sample size calculation 52
9.7 Power for analysis of key secondary variables 53
9.8 Interim analyses 53
10 Ethical considerations 53
10.1 Regulatory and ethical compliance 53
10.2 Informed consent procedures 53
10.3 Responsibilities of the investigator and IRB/IEC 54
10.4 Publication of study protocol and results 54
11 Protocol adherence 54
11.1 Protocol Amendments 55
12 References 55
13 Appendix 1: Clinically notable laboratory values and vital signs 57

PPD [redacted]

14 Appendix 2: Liver event definitions and follow-up requirements 57
15 Appendix 3: Child-Pugh Clinical Assessment Score 60
16 Appendix 4: Sample log table 61

List of tables

Table 5-1 Prohibited treatment 27
Table 6-1 Assessment schedule 29
Table 9-1 Estimated number of patients required for the 90% CI on mean change from baseline to exclude zero 52
Table 9-2 Estimated number of patients required for the 90% CI on mean change from baseline to exclude zero 53
Table 14-1 Liver Event Definitions 57
Table 14-2 Liver Event Follow Up Requirements 58
Table 16-1 Sample collection time points for pharmacokinetic (PK), immunogenicity (IG) and biomarker analyses from all patients receiving serelaxin study treatment (portal vein blood sampling in part B only) 61

List of figures

Figure 3-1 Study design 15

PPD [redacted]

List of abbreviations

ADA	Anti-drug antibody
AE	Adverse event
ALT	Alanine aminotransferase
AST	Aspartate aminotransferase
BMI	Body mass index
eCRF	Electronic case report/record form
CHF	Chronic heart failure
CRO	Contract research organization
ECG	Electrocardiogram
EDC	Electronic data capture
GGT	γ-Glutamyltransferase
HRS	Hepatorenal syndrome
HSC	Hepatic stellate cell
HVPG	Hepatic venous pressure gradient
IB	Investigator brochure
ICH	International Conference on Harmonization of Technical Requirements for Registration of Pharmaceuticals for Human Use
(I)EC	(Independent) ethics committee
IG	Immunogenicity
INR	International normalized ratio
i.v.	Intravenous
IVCP	Inferior vena cava pressure
IRB	Institutional review board
IHVR	Intrahepatic vascular resistance
MRA	Magnetic resonance angiography
MRI	Magnetic resonance imaging
PHT	Portal hypertension
PK	Pharmacokinetic
PPG	Portal pressure gradient
PT	Prothrombin time
PVP	Portal vein pressure

PPD [redacted]

RAP	Right atrial pressure
SAE	Serious adverse event
SBP	Systolic blood pressure
s.c.	Subcutaneous
SGPT	Glutamic pyruvic transaminase
TIPSS	Transjugular intrahepatic portosystemic shunt

PPD [redacted]

Glossary of terms

Assessment	A procedure used to generate data required by the study
Enrollment	Point/time of patient entry into the study; the point at which informed consent must be obtained (i.e. prior to starting any of the procedures described in the protocol)
Investigational drug	The study drug whose properties are being tested in the study; this definition is consistent with US CFR 21 Section 312.3 and is synonymous with “investigational new drug.”
Patient number	A number assigned to each patient who enrolls in the study. When combined with the center number, a unique identifier is created for each patient in the study.
Period	A minor subdivision of the study timeline; divides phases into smaller functional segments such as screening, baseline, titration, washout, etc.
Premature patient withdrawal	Point/time when the patient exits from the study prior to the planned completion of all study drug administration and assessments; at this time all study drug administration is discontinued and no further assessments are planned
Randomization number	A unique identifier assigned to each randomized patient, corresponding to a specific treatment arm assignment
Stage	A major subdivision of the study timeline; begins and ends with major study milestones such as enrollment, randomization, completion of treatment, etc.
Stop study participation	Point/time at which the patient came in for a final evaluation visit or when study drug was discontinued whichever is later
Study drug	Any drug administered to the patient as part of the required study procedures; includes investigational drug and any control drugs
Study drug discontinuation	Point/time when patient permanently stops taking study drug for any reason; may or may not also be the point/time of premature patient withdrawal
Variable	Information used in the data analysis; derived directly or indirectly from data collected using specified assessments at specified timepoints

PPD [redacted]

Protocol synopsis

Title of study: An exploratory study to investigate the haemodynamic effects of serelaxin in patients with compensated cirrhosis and portal hypertension.

Purpose and rationale: The purpose of this exploratory study is to investigate the effect of serelaxin infusion on the hepatic and renal circulation in patients with compensated cirrhosis and portal hypertension.

Using MRA in part A of the study, the effects of serelaxin on the hepatic, splanchnic, renal and systemic circulations will be evaluated. Taken together, these measurements will establish the potential for serelaxin as a therapy in cirrhosis (e.g. to reduce PHT in variceal bleeding and/or to increase renal blood flow in hepatorenal syndrome). Terlipressin (a proven haemodynamic modulator in cirrhosis) will be used in this study to establish the sensitivity and dynamic range of the employed MRI method. Although MRA enables a broad assessment of blood flow changes, it does not directly measure portal vein pressure which is associated with fewer complications and decreased mortality if kept below defined thresholds. Therefore, effects of serelaxin on portal vein pressure will be evaluated in part B of the study: Direct portal vein pressure measurements will be performed in patients with a TIPSS *in situ* at the time of portal vein catheterization for routine portography.

Objectives: Part A: To investigate whether serelaxin increases the total renal arterial blood flow in patients with cirrhosis and PHT after at least 120 min of infusion (60 min at 80 µg/kg/day and at least 60 min at 30 µg/kg/day); Part B: To investigate whether serelaxin reduces the portal pressure gradient in patients with cirrhosis, PHT and a TIPSS in situ after at least 120 min of infusion (60 min at 80 µg/kg/day and at least 60 min at 30 µg/kg/day).

Study design: This exploratory study uses a randomized, open-label, non-controlled, parallel-group design and consists of two parts that are planned to be run in parallel.

Population: Part A: A total of 40 male or female participants with compensated alcohol-related cirrhosis and portal hypertension, who do not receive or plan to use any drug to treat portal hypertension; Part B: A total of 6 male or female participants with compensated alcohol-related cirrhosis and portal hypertension, who have a fully functioning TIPSS in situ and do not receive or plan to use any drug to treat portal hypertension.

Inclusion/Exclusion criteria:

Key inclusion criteria (part A and B):

- Male and female patients 18 to 75 years of age (inclusive).
- Cirrhosis of alcohol aetiology according to physician's assessment prior to screening.

Key inclusion criteria (part A):

- Cirrhosis with clinical and/or endoscopic evidence of portal hypertension (e.g. oesophageal varices).

Key inclusion criteria (part B):

- Cirrhosis with TIPSS in situ and PPG>5mmHg.
- Fully functioning TIPSS without variceal filling as confirmed by portography at Visit 2.

Key exclusion criteria:

- Use of any drug to treat portal hypertension (e.g. vasodilators such as non-selective beta blockers or nitrates) within 1 month prior to screening and no plan for use until visit 2.
- Decompensated cirrhosis (Child-Pugh score >9 points, and/or ascites requiring diuretics, and/or hepatic encephalopathy) at visit 1.
- History of drug or alcohol abuse within 1 month prior to visit 2.

- Presence of any non-controlled and clinically significant disease that could affect the study outcome or that would place the patient at undue risk.
- History of variceal bleed within 1 month prior to visit 1.
- Sitting SBP <110 mmHg at visits 1 and/or 2.
- Severe renal impairment (eGFR <30 mL/min).

Key exclusion criteria (Part A):

- Contraindication to terlipressin acetate.
- Body mass index (BMI) (weight[kg] / height[m]^2) > 40 kg/m^2.
- Any contraindication to having an MRI scan

Investigational and reference therapy:

- Terlipressin (2mg i.v. bolus)
- Serelaxin(RLX030) (i.v. infusion: 80 µg/kg/d for 60 min followed by 30 µg/kg/d for at least 60 min)

Efficacy assessments:

- Part A: Blood flow measured via MRA
- Part B: Direct venous pressure measurement

Other assessments:

- Physical examination
- Vital signs
- Local and central ECG
- Laboratory safety evaluations (hematology and clinical chemistry)
- Biomarkers (only in patients who receive serelaxin)
- Immunogenicity (only in patients who receive serelaxin)
- Pharmacokinetics (only in patients who receive serelaxin)

Data analysis: Part A: Summary statistics (n, mean, SD, median, quartiles, min, max, geometric mean) for the baseline, post baseline and change from baseline measurements for the blood flow parameter for patients in the serelaxin group will be presented. Confidence intervals on the change from baseline for the blood flow parameter will be calculated for both the arithmetic and geometric means. Data analysis for the terlipressin acetate group will be analyzed in a similar manner as the serelaxin group. **Part B:** Summary statistics (n, mean, SD, median, quartiles, min, max, geometric mean) for the baseline, post baseline and change from baseline measurements for each parameter will be presented. Confidence intervals on the change from baseline for the parameters will be calculated for both the arithmetic and geometric means.

Amendment 1

Amendment rationale

The reasons for this amendment are to correct the minimum number of patients required for Part A and to modify the number of subjects that will be enrolled into Part B. For Part A, the changes to the minimum values do not affect the statistical rationale as these minimum numbers are well below the number currently enrolled in Part A (see Table 9-1). For Part B, 10 patients had originally been planned (above the minimum of six required), as they were expected to have been enrolled within a reasonable time frame. However, during the course of the trial, it became apparent that a significant number of patient candidates for Part B had

PPD [redacted]

advanced to a disease stage where certain exclusion criteria were now limiting factors for recruitment, including low blood pressure (SBP <110 mmHg) and requirement for diuretics. Additionally, the PPG 'window' for treatment (between 5 and 12 mmHg in most patients) has proven to be rather narrow. Thus, for clinical enrollment reasons, the decision was made to reduce the number of patients enrolled in Part B from ten to six. The statistical rationale is not affected; six patients represents the minimum required for the 90% CI on mean change from baseline to exclude zero for each endpoint (PPG gradient, PVP, RAP, see Table 9-2).

A copy of this amended protocol will be sent to the Institutional Review Board (IRB)/Independent Ethics Committee (IEC) and Health Authorities. The changes described in this amended protocol require IRB/IEC approval prior to implementation. In addition, the global sample informed consent was modified per this amendment to change the number of subjects from 10 to 6. At the time of this amendment, Part A has completed enrollment and Part B has randomized 4 subjects.

1 Introduction

1.1 Background

Cirrhosis is a consequence of chronic liver disease characterised by replacement of liver tissue by fibrosis, scar tissue and regenerative nodules leading to loss of liver function. Portal hypertension (PHT) (ie. high blood pressure in the hepatic portal vein and its tributaries), is a frequent manifestation of cirrhosis and accounts for most of the serious complications of this disease (Sanyal et al 2008). The exact prevalence of cirrhosis worldwide is unknown. It was estimated at 0.15% or 400,000 in the USA, which accounted for more than 25,000 deaths and 373,000 hospital discharges in 1998. Similar numbers have been reported from Europe and numbers are even higher in Asian and African countries where chronic viral Hepatitis B and C are common (Schuppan and Afdhal 2008). In the UK, cirrhosis mortality rate is rising (Leon and McCambridge 2006). With changing patterns of alcohol consumption and increasing rates of obesity and diabetes in Western countries, deaths from the complications of cirrhosis are expected to triple in the next decade.

In cirrhosis, PHT is initiated by increased resistance to portal blood flow and aggravated by increased portal-collateral blood flow. Increased intrahepatic vascular resistance (IHVR) is generated by two factors: firstly, mechanical obstruction due to distortion of the hepatic circulation by vascular occlusion, fibrosis, nodule formation & angiogenesis and secondly, a dynamic component mediated by active contraction of myofibroblasts predominantly derived from activated hepatic stellate cells (HSCs) around the hepatic sinusoids and in fibrous septa, and vascular smooth muscle cells (de Franchis 2000). The dynamic component accounts for ~30% of the increased IHVR in cirrhosis. This reflects a functional disturbance of the liver circulation (Bhathal and Grossman 1985), secondary to increased production of vasoconstrictors (including endothelins and angiotensin II) and reduced release of endogenous vasodilators mainly nitric oxide (NO). As such this represents an important target for future therapy (Langer and Shah 2006).

The hepatorenal syndrome (HRS) is characterised by the development of renal failure in patients with severe liver disease (acute or chronic) in the absence of any other identifiable cause of renal pathology. It carries a very poor prognosis (Arroyo et al 2008). The survival

expectancy is very low and spontaneous recovery very rare. In Type 1 HRS hospital survival is less than 10% and the expected median survival time only 2 weeks (Ginès et al 2003). In cirrhosis the annual incidence rate of HRS is estimated at 8% to 40% (Munoz 2008, Ginès et al 1993). The frequency of HRS in severe acute alcoholic hepatitis and in fulminant liver failure is about 30% and 55%, respectively (Fernández et al 2007). The hallmark of HRS is reversible renal vasoconstriction, while the kidneys are structurally normal and with intact function at least in the early part of the syndrome. The causes of renal vasoconstriction are unknown but may involve both increased vasoconstrictor and decreased vasodilator factors acting on the renal circulation. All the drugs that have been investigated in HRS to date target to increase renal blood flow either indirectly by splanchnic vasoconstriction or directly by using renal vasodilators. One of the constraints with vasoconstrictors has been the lack of agents which act purely on the splanchnic circulation; the "spill over" into the systemic circulation may actually exacerbate the intense renal vasoconstriction already present or impact other organ systems.

A reduction in portal pressure below defined thresholds is associated with fewer complications and decreased mortality (Ripoll 2007). Although non-selective beta blockers have remained the cornerstone of treatment for PHT for over 20 years, their use is limited by the small number of patients who have a haemodynamic response (20%–30%), intolerance to therapy in 10%–20% because of peripheral vasodilation and hypotension and rebound PHT (if discontinued suddenly). In the acute setting (variceal bleeding), the drug terlipressin is used extensively in the UK. Terlipressin is a synthetic vasopressin analogue that releases its active form, lysine vasopressin, after 3 glycyl residues are cleaved by endogenous proteases. Terlipressin induces splanchnic vasoconstriction (thereby reducing portal pressure), increases systemic blood pressure and has a renoprotective effect in a proportion of patients. The systemic vasoconstrictor effects of terlipressin could also lead to ischaemic complications and less than half of patients respond to this treatment (Nazar et al 2010). Repeated endoscopic variceal ligation may be needed to stop variceal bleeding.

Continued bleeding, severe early rebleeding, and repeated recurrent bleeding is managed with transjugular intrahepatic portosystemic shunt (TIPSS). TIPSS is a radiologic procedure by which a tract is created between hepatic and portal vein and this tract is kept open by deployment of a stent. Current treatment options are limited. Alternative therapies that are safer and more effective are urgently needed.

Relaxin is a peptide hormone with antifibrotic, haemodynamic and immunomodulatory effects in a variety of tissues. Treatment with relaxin exerts an antifibrotic effect *in vivo* in experimental rodent models of liver, dermal, lung, renal and cardiac fibrosis (Bennett 2009). Relaxin has also been shown to cause dilatation of blood vessels including arterioles, in a variety of tissues such as the heart, lung and kidney, irrespective of gender (Conrad 2010). In the kidney, the time course and dose response of relaxin mediated vasodilation, hyperfiltration and changes in plasma osmolality have been defined in conscious rats (Danielson and Conrad 2003). Furthermore, administration of relaxin in healthy humans increased renal blood flow by 47% in both males and females. The *in vitro* and *in vivo* models provide compelling evidence for a vasoactive role of relaxin in the liver. In addition to inhibiting HSC contractility *in vitro*, relaxin infusion induces a rapid, selective and significant reduction in portal pressure in cirrhotic, portal hypertensive rats (Fallowfield et al 2014). Relaxin had no significant effect on systemic arterial blood pressure in this model.

PPD [redacted]

In view of its homeostatic role in physiological processes and its potential therapeutic role in pathological disease states, serelaxin (recombinant human relaxin-2) has undergone clinical evaluation in a number of indications, including scleroderma, cervical ripening, pre-eclampsia and acute heart failure and information about these studies can be found in the Investigator Brochure (IB). In particular, data from the multicentre phase II PRE-RELAX trial (Teerlink et al 2009) and emerging data from the phase III RELAX trial in acute heart failure reinforce the potential utility of serelaxin as a safe and effective haemodynamic modulator.

1.2 Purpose

The purpose of this exploratory study is to investigate the effect of serelaxin infusion on the hepatic and renal circulation in patients with compensated cirrhosis and portal hypertension.

2 Study objectives

2.1 Primary objectives

Part A (Magnetic resonance angiography (MRA))

To investigate whether serelaxin increases the total renal arterial blood flow in patients with cirrhosis and PHT after at least 120 min of infusion (60 min at 80 µg/kg/day and at least 60 min at 30 µg/kg/day).

Part B (direct venous pressure measurement)

To investigate whether serelaxin reduces the portal pressure gradient in patients with cirrhosis, PHT and a TIPSS in situ after at least 120 min of infusion (60 min at 80 µg/kg/day and at least 60 min at 30 µg/kg/day).

2.2 Secondary objectives

Part A (MRA)

- To investigate whether changes in blood flow associated with terlipressin acetate in patients with cirrhosis and PHT are detected by measurement with MRA at least 120 min after 2 mg iv bolus administration.
- To evaluate whether serelaxin changes the hepatic arterial blood flow in patients with cirrhosis and PHT after at least 120 min of infusion (60 min at 80 µg/kg/day and at least 60 min at 30 µg/kg/day).
- To evaluate whether serelaxin changes the flow in the superior mesenteric artery in patients with cirrhosis and PHT after at least 120 min of infusion (60 min at 80 µg/kg/day and at least 60 min at 30 µg/kg/day).
- To evaluate whether serelaxin changes the flow in the descending thoracic aorta in patients with cirrhosis and PHT after at least 120 min of infusion (60 min at 80 µg/kg/day and at least 60 min at 30 µg/kg/day).

- To evaluate whether serelaxin changes the portal vein blood flow in patients with cirrhosis and PHT after at least 120 min of infusion (60 min at 80 µg/kg/day and at least 60 min at 30 µg/kg/day).
- To collect information on safety and tolerability of serelaxin in patients with cirrhosis and PHT.

Part B (direct venous pressure measurement)

- To evaluate whether serelaxin decreases portal vein pressure in patients with cirrhosis, portal hypertension and a TIPSS *in situ* after at least 120 min of infusion (60 min at 80 µg/kg/d and at least 60 min at 30 µg/kg/d).
- To collect information on safety and tolerability of serelaxin in patients with cirrhosis, portal hypertension and a TIPSS *in situ*.

2.3 Exploratory objectives

Part A and B

- To explore whether serelaxin changes selected biomarkers in patients with compensated cirrhosis and portal hypertension after at least 120 min of infusion (60 min at 80 µg/kg/day and at least 60 min at 30 µg/kg/day).
- To explore the serelaxin pharmacokinetics in patients with compensated cirrhosis and portal hypertension.
- To explore serelaxin immunogenicity in patients with compensated cirrhosis and portal hypertension.

Part A (MRA)

- To explore further haemodynamic effects of serelaxin in patients with cirrhosis and portal hypertension.
- To explore the role of MRA as a non-invasive modality for measuring changes in blood flow in response to vasoactive agents in patients with cirrhosis and portal hypertension.

Part B (direct venous pressure measurement)

- To explore the speed of onset of portal vein pressure reduction induced by serelaxin.

3 Investigational plan

3.1 Study design

This exploratory study uses a randomized, open-label, non-controlled, parallel-group design and consists of two parts that are planned to be run in parallel at a single center in the UK. The study design is outlined in Figure 3-1.

PPD [redacted]

Figure 3-1 Study design

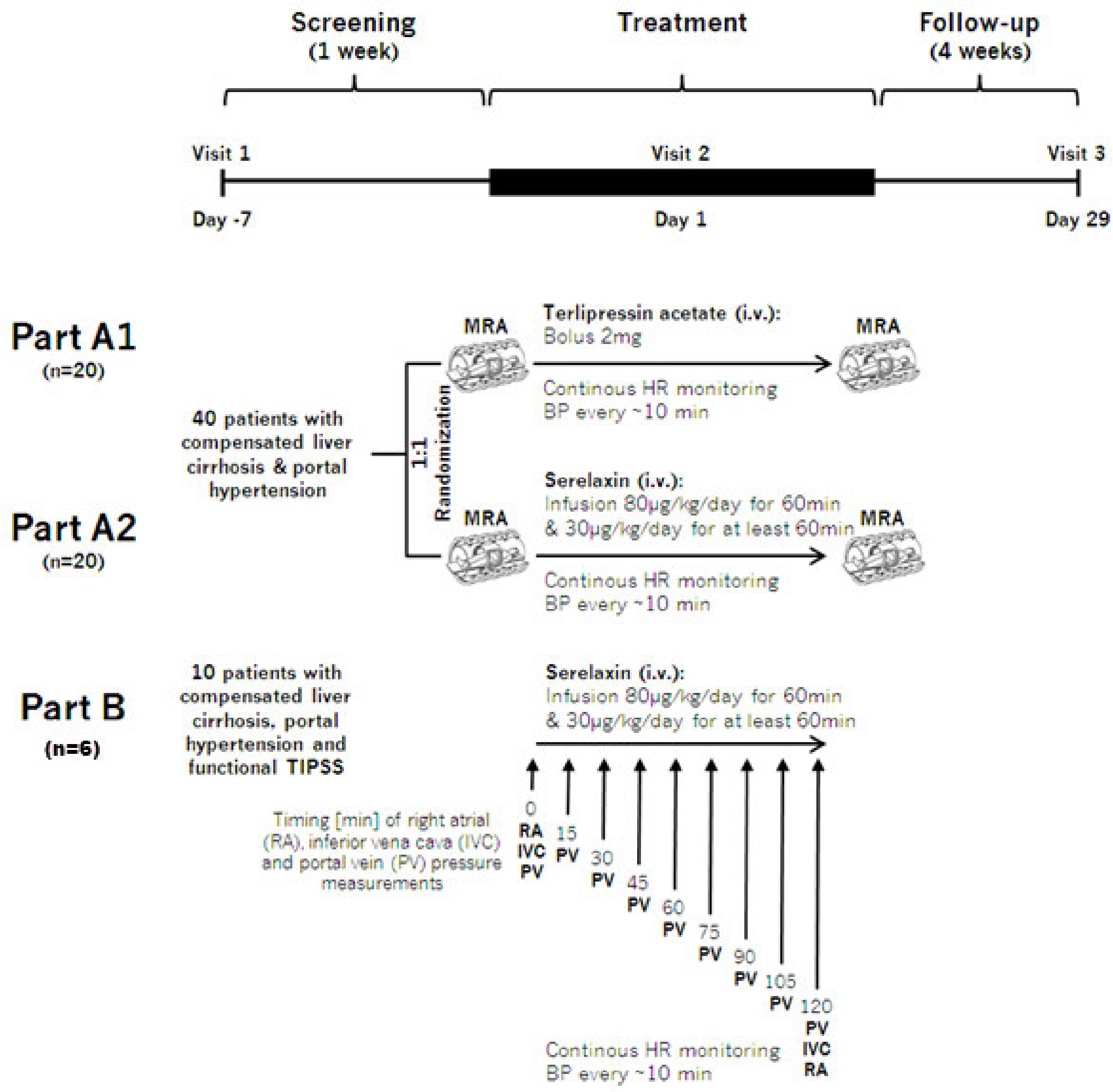

Part A uses a randomized, parallel-group design to investigate blood flow changes measured via MRA. A screening period of about one week will be used to assess the patients' eligibility for study participation. At the Baseline visit (Visit 2, Day 1) eligible patients will be randomized to receive either terlipressin acetate (part A1) or serelaxin (part A2) and undergo the baseline MRA assessment. The terlipressin acetate group will receive an i.v. bolus dose of 2 mg while the serelaxin group will receive an i.v. serelaxin infusion at two different infusion rates: 80 µg/kg/d for 60 min followed by 30 µg/kg/d for at least 60 min. 120 minutes after receiving the terlipressin acetate bolus dose or after starting the serelaxin infusion respectively, the second MRA assessment will be performed while the serelaxin infusion is continued in part A2. When the second MRA as well as the other 120 min assessments have been completed, the serelaxin infusion with an expected maximum duration of ~180 min will be

PPD [redacted]

stopped and all patients will be observed for at least 60 minutes to ensure their well-being (recovery period). The heart rate will be continuously monitored and systemic blood pressure will be recorded at baseline, approximately every 10 min until completion of the 120 min assessments and approximately every 15 min until completion of the 60 minutes recovery period which follows after the second MRA assessment. ECGs will be prepared at baseline, approximately 60 min after initiating study drug treatment, after completion of the second MRA and at the end of recovery period. A follow-up visit is scheduled about 4 weeks later (Visit 3, Day 29) for the patients' safety.

Part B uses a single group design and is planned to be run in parallel to part A. Patients with a TIPSS *in situ* who are due for their yearly routine shunt assessment will start to be screened for study participation about one week prior to their planned shunt assessment. After eligibility for study participation has been confirmed (incl. the confirmation of a fully functioning shunt without variceal filling and increased portal vein pressure at Visit 2, Day 1) the patients will receive an i.v. serelaxin infusion at two different infusion rates: 80 µg/kg/d for 60 min followed by 30 µg/kg/d for at least 60 min. Recordings of portal vein pressure will be taken at baseline and at 15 min intervals during i.v. serelaxin infusion. The right atrial and the inferior vena cava pressure will be measured twice: Prior to the portal vein pressure baseline measurement (at 0 minutes) and after the last portal vein pressure measurement at ~120 minutes of infusion. The serelaxin infusion with an expected maximum duration of ~130 min will only be stopped after the second right atrial pressure has been measured and the other 120 min assessments have been completed. The heart rate will be continuously monitored and systemic blood pressure will be recorded at baseline, approximately every 10 min until completion of the 120 min assessments and approximately every 15 min until completion of the 60 minutes recovery period which follows after the last direct pressure measurement. ECGs will be performed at baseline, approximately 60 and 120 min starting serelaxin infusion, and at the end of recovery period. A follow-up visit is scheduled about 4 weeks later (Visit 3, Day 29) for the patients' safety.

Blood and urine samples will be collected from all patients receiving serelaxin treatment for biomarker analysis. Blood samples will be collected from all patients receiving serelaxin treatment for pharmacokinetic and immunogenicity assessments.

All patients should be fasting for at least 8 hours prior to any study visit (no food, coffee, tea, herbal tea, fruit tea, fruit juice, milk, soda, broth, or any other liquids are allowed except unsweetened water). Patients should be kept fasting throughout the MRA/direct venous pressure measurement procedure (sips of unsweetened water are allowed to keep the patients comfortable).

Any drugs to treat portal hypertension (e.g., vasodilators such as non-selective beta blockers or nitrates) will be prohibited within the period of 1 month before screening to visit 2. Use of diuretics will be prohibited from screening (visit 1) to visit 2. However, washout of these prohibited drugs will not be performed during or prior screening with the intention to make a patient eligible for study participation.

PPD [redacted]

3.2 Rationale of study design

Part A

This randomized, open-label, parallel-group study design with planned analyses of intra-group changes only was selected for this exploratory trial, as it is appropriate to achieve the defined study objectives. No substantial improvement would be provided in the study's rigor by introduction of double-blinding.

Between-group comparisons are not considered meaningful since different effects on local blood flows are already expected for terlipressin acetate and serelaxin due to their different mechanisms of action.

The terlipressin group is exclusively used in this study to establish the sensitivity and dynamic range of the employed magnetic resonance imaging (MRI) method to detect hemodynamic changes as caused by a drug that is proven to be efficacious and to significantly change hemodynamics.

The study has a four week follow-up period because this is considered the best interval for detecting any potential anti-serelaxin antibody development. Additionally, past data in systemic sclerosis patients who developed anti-serelaxin antibodies after chronic sc administration of serelaxin over weeks demonstrated that serelaxin levels in either antibody negative or positive subjects returned to baseline within 4 weeks after stopping serelaxin administration.

The total renal artery flow has been chosen as the primary endpoint parameter of part A because studies indicate that increases in portal pressure result in decreases in GFR and renal blood flow and increases in renin secretion mediated by increased renal adrenergic tone (Anderson 1976). These parameters are most markedly altered in HRS, the hallmark of which is severe but reversible renal vasoconstriction. Serelaxin has the potential to increase renal blood flow in the context of cirrhotic portal hypertension and HRS.

Part B

No comparator group is used for Part B, as the purpose is to establish the size of a serelaxin treatment effect. If a comparator was used, this would significantly increase the sample size with minimal benefit. At the same time, the increased number of patients adds more patients to be exposed to study treatment and risks.

The portal pressure gradient (PPG) (i.e. the difference between the portal venous pressure and the inferior vena cava pressure) has been chosen as the primary endpoint parameter of part B because the measurement of an elevated PPG is used to define the presence of portal hypertension. Moreover, the magnitude of PPG has prognostic importance in cirrhosis and a reduction in portal pressure in response to therapy leads to an improvement in survival and clinical outcomes (Abraldes et al 2003).

3.3 Rationale of dose/regimen, duration of treatment

There is no ideal portal hypertension animal model available that would support estimation of the effective dose or concentration of serelaxin in this indication. Therefore, it is assumed that

PPD [redacted]

a steady state concentration similar to the efficacious serum serelaxin concentration in acute heart failure patients would be needed for patients with portal hypertension. In addition, the pharmacokinetic (PK) of serelaxin has already been studied in subjects with mild, moderate, or severe hepatic impairment along with healthy control subjects (Protocol RLX030A2101). Preliminary analysis for that study suggests that there is no significant impact of hepatic impairment on the PK of serelaxin. Since the serelaxin infusion time cannot be extended beyond 2-3 hours in this study for practical reasons, modeling and simulation were performed using a population PK approach with the serelaxin PK data from the hepatic impairment study (Protocol RLX030A2101). The goal of the modeling and simulation was to identify a dosing regimen that can achieve serelaxin serum concentrations within less than 2 hours that are comparable to the steady-state concentrations that have shown to be efficacious in patients with acute heart failure. This PK simulation suggests that a dosing with two different infusion rates (80 µg/kg/day for 60 minutes followed by 30 µg/kg/day for 60 minutes) will lead to the desired steady-state concentrations within about an hour.

Based on available pre-clinical and clinical data from other indications it seems unlikely that a higher serelaxin steady-state concentration would be required to achieve the pharmacodynamic effects as investigated in this study (i.e. blood flow increases in target vascular territories and portal vein pressure decrease).

The fastest serelaxin infusion rate administered in a clinical trial setting was 960 µg/kg/day and it was found to be well tolerated. Therefore, the selected dosing of 80 µg/kg/day for 60 minutes followed by 30 µg/kg/day for up to 120 minutes (to continue the serelaxin infusion until completion of 120 min MRA assessment/portal pressure measurement) is considered to be safe for the study participants.

Since serelaxin is expected to be a fast onset treatment, a treatment infusion of at least 120 minutes duration is considered sufficient to show pharmacodynamic effects.

The i.v. route of administration has been chosen, because it would be optimal for the treatment of portal hypertension patients in the acute setting (i.e., acute variceal bleeding). Additionally, it may also render serelaxin less immunogenic than delivery by the s.c. route (Rosenberg and Worobec 2004). In previously conducted clinical studies, serelaxin was administered by continuous s.c. infusion over periods of 24 weeks or longer in patients with scleroderma. Overall, antibody formation occurred in 30% of patients. Because administration in the proposed study is via the i.v. route, is relatively short term, and in a non-autoimmune population, the risk of antibody formation is considered low. However, monitoring for antibody formation will be performed.

3.4 Rationale for choice of comparator

Terlipressin, a synthetic vasopressin analogue, is commercially available in the UK and represents the standard pharmacological treatment for bleeding varices. It has been shown to decrease portal pressure and hepatic and renal arterial resistance in patients with cirrhosis (Narahara et al 2009). The i.v. bolus dose of 2mg terlipressin acetate that will be used in this study is the recommended starting dose for treatment of bleeding varices according to the product's labelling.

PPD [redacted]

Terlipressin acetate is primarily used in this trial to demonstrate the range of sensitivity of the MRA method to detect blood flow changes caused by an efficacious drug rather than to directly compare its effects to the potential effects of serelaxin. Differences in the pharmacodynamic effects of these two drugs are already expected due to their different mechanism of actions and therefore, direct efficacy comparisons for this study's hemodynamic efficacy parameters would not be meaningful.

The use of a placebo group was not chosen for this exploratory study, as the objective was to determine whether a change was occurring while still maintaining a relatively small study. Inclusion of a placebo group would require an increase in the sample size for both the placebo and serelaxin groups to provide appropriate power to show differences between treatments. Therefore, intra-group analyses are planned comparing baseline to post treatment for this study to demonstrate that a measureable change occurs and can be detected using the MRA methodology.

3.5 Purpose and timing of interim analyses/design adaptations

Not applicable.

3.6 Risks and benefits

A total of 788 subjects have been treated with serelaxin in 23 completed clinical studies across multiple indications. Serelaxin showed an acceptable safety profile and was well tolerated at all doses (6 - 960 µg/kg/day). Adverse events (AEs) and serious adverse events in the clinical trials were evenly distributed across treatment groups within each of the studies and reflected the natural course and the seriousness of the underlying disease. Nevertheless, no study has been performed in patients with cirrhosis and portal hypertension. The potential risks for patients participating in this study are listed below.

Risks associated with insertion of intravenous (i.v.) cannula

This procedure carries a theoretical risk for the participant of local and systemic infection at the time of insertion or while *in situ*.

Risks associated with intravenous infusion of serelaxin

The following adverse reactions have been reported for serelaxin in the completed clinical trials, which may represent potential safety risks for this study:

- Because serelaxin has vasodilatory properties, blood pressure decreases (defined as a SBP decrease >40 mmHg or any SBP measurements of less than 100 mmHg) were reported with serelaxin in chronic heart failure (CHF) patients. Serelaxin treatment of systemic sclerosis was also associated with decreases in blood pressure generally within normal ranges. Patients should have blood pressure monitoring while receiving serelaxin and it should not be administered to patients who are at risk of developing hypotension during vasodilator therapy.
- Increases in serum creatinine have been observed in CHF subjects receiving serelaxin but were asymptomatic.
- Menometrorrhagia, which may be associated with anaemia. Based on the pharmacology of serelaxin, menorrhagia, metrorrhagia, and dysmenorrhea are not unexpected consequences

of serelaxin administration. These events did not frequently result in discontinuation, and with lower doses and/or local administration, they may not occur.

- In CHF patients, a decrease in hemoglobin/hematocrit (up to 7%) occurred within hours of dosing but was asymptomatic.
- Antibody formation to serelaxin has occurred in 30% of subjects receiving serelaxin, but only after chronic s.c. administration. To date, the antibodies have been associated with no apparent adverse events. There is no evidence that the antibodies are neutralizing, nor is there evidence of any long-term sequelae.
- As with any protein, there is a possibility of hypersensitivity reactions, such as itching or skin rash, as well as the possibility of a potentially serious allergic reaction, which may result in hospitalization or even death. To date, no serious allergic reaction has been reported in the clinical studies.

Risks associated with intravenous injection of terlipressin acetate

Since terlipressin acetate has vasoconstrictor activity it should be used with great caution in patients with hypertension, atherosclerosis, cardiac dysrhythmias or coronary insufficiency. Monitoring of pulse and blood pressure is therefore essential.

Abdominal cramps, headache, transient blanching and increased arterial blood pressure have been noted with use of terlipressin acetate according to the Glypressin® labelling.

Risks associated with fluoroscopic monitoring

Interventional fluoroscopy uses ionizing radiation to guide small instruments such as catheters through blood vessels or other pathways in the body. The benefits of properly performed interventional fluoroscopy almost always outweigh the radiation risk experienced by an individual. The short-term risk to patients is radiation-induced skin damage and longer term effects include the potential risk of cancer. Strategies to manage radiation dose to patients and operators are employed as standard and include well trained operators using proper radiological technique and minimization of fluoroscopy time. Due to the anticipated short screening time involved (catheter positioning only), the risks associated with the use of fluoroscopy in this study are considered minimal.

Minimising risk

The risk to subjects in this trial will be minimized by compliance with the eligiblity criteria and close clinical monitoring in a hospital setting. Potential risks associated with MRI procedures are minimal (e.g. claustrophobia), as these are widely used clinical procedures. Subjects who suffer from severe claustrophobia will be excluded as specified in the exclusion criteria. Non-contrast MRA will be employed in order to avoid the use of Gadolinium based contrast material and therefore to remove the risk of renal failure and nephrogenic systemic fibrosis for participants.

Risks associated with intravenous cannulation

The commonest risk factors for i.v. cannula related infections are inadequate site preparation and duration of cannula insertion. Both of these factors will be minimized by the use of sterile technique and by the short duration (few hours) of catheter insertion.

Risks associated with intravenous infusion of serelaxin

Patients with a baseline SBP<110mmHg will be excluded from the study. Blood pressure will be regularly monitored during the infusion and for 60 minutes after the infusion has been stopped. Signs or symptoms of hypotension or blood pressure less than either SBP <90 and/or DBP <50 mmHg will be thoroughly evaluated by the investigator and if clinically warranted, the patient will be permanently discontinued from study drug. Further interventions (e.g. administration of i.v. fluid) will be at the discretion of the medically qualified investigator in attendance.

Risks associated with intravenous injection of terlipressin acetate

Patients with a recent history of myocardial ischaemia will be excluded from the study. Blood pressure and pulse/heart rate will be regularly monitored during the study.

Potential Benefit

There is no direct benefit expected for patients participating in this study.

4 Population

Part A (MRA)

A total of 40 male or female participants with compensated alcohol-related cirrhosis and portal hypertension, who do not receive or plan to use any drug to treat portal hypertension.

Part B (direct portal vein pressure measurement)

A total of 6 male or female participants with compensated alcohol-related cirrhosis and portal hypertension, who have a fully functioning TIPSS *in situ* and do not receive or plan to use any drug to treat portal hypertension.

4.1 Inclusion criteria

Patients eligible for inclusion in this study have to fulfill all of the following criteria:

All participants (Part A and B)

1. Male and female patients 18 to 75 years of age (inclusive).
2. Cirrhosis of alcohol aetiology according to physician's assessment prior to screening.
3. Written informed consent must be obtained before any assessment is performed.

Part A (MRA)

4. Cirrhosis with clinical and/or endoscopic evidence of portal hypertension (e.g. oesophageal varices).

Part B (direct venous pressure measurement)

5. Cirrhosis with TIPSS in situ and PPG>5mmHg.
6. Fully functioning TIPSS without variceal filling as confirmed by portography at Visit 2.

PPD [redacted]

4.2 Exclusion criteria

Patients fulfilling **any** of the following criteria are not eligible for inclusion in this study. No additional exclusions may be applied by the investigator, in order to ensure that the study population will be representative of all eligible patients.

All participants (Part A and B)

1. Use of any drug to treat portal hypertension (e.g. vasodilators such as non-selective beta blockers or nitrates) within 1 month prior to screening and no plan for use until visit 2.
2. Decompensated cirrhosis (Child-Pugh score >9 points (see Appendix 3), and/or ascites requiring diuretics, and/or hepatic encephalopathy) at visit 1.
3. History of drug or alcohol abuse within 1 month prior to visit 2.
4. Presence of any non-controlled and clinically significant disease that could affect the study outcome or that would place the patient at undue risk.
5. Any surgical or medical condition other than hepatic impairment which might significantly alter the distribution or excretion of drugs, or which may jeopardize the safety of the study subject in case of participation in the study. The Investigator should make this determination in consideration of the patient's medical history and/or clinical or laboratory evidence of any of the following:
   - History or presence of severely impaired renal function.
   - Evidence of urinary obstruction.
6. Hepatocellular carcinoma.
7. Portal or splenic vein thrombosis.
8. History of variceal bleed within 1 month prior to visit 1.
9. Sitting SBP <110 mmHg at visits 1 and/or 2.
10. Severe renal impairment (eGFR <30 mL/min).
11. History of hypersensitivity to any of the study drugs or to drugs of similar chemical classes.
12. Use of other investigational drugs within 5 half-lives of enrollment, or within 30 days/until the expected pharmacodynamic effect has returned to baseline, whichever is longer.
13. Long QT syndrome or QTc > 450 msec (QT correction will be performed using the Fridericia correction method: QTcF = QT/RR0.33) for males and > 470 msec for females at screening (visit 1).
14. History of malignancy of any organ system (other than localized basal cell carcinoma of the skin), treated or untreated, within the past 5 years, regardless of whether there is evidence of local recurrence or metastases.
15. Pregnant or nursing (lactating) women, where pregnancy is defined as the state of a female after conception and until the termination of gestation, confirmed by a positive hCG laboratory test.
16. Women of child-bearing potential, defined as all women physiologically capable of becoming pregnant, unless they will be using highly effective methods of contraception

during entire study (i.e. from screening visit 1 onwards). **Highly effective** contraception methods include:

- Total abstinence (when this is in line with the preferred and usual lifestyle of the subject). Periodic abstinence (e.g., calendar, ovulation, symptothermal, post-ovulation methods) and withdrawal are not acceptable methods of contraception
- Female sterilization (have had surgical bilateral oophorectomy with or without hysterectomy) or tubal ligation at least six weeks before taking study treatment. In case of oophorectomy alone, only when the reproductive status of the woman has been confirmed by follow up hormone level assessment
- Male sterilization (at least 6 months prior to screening). For female subjects on the study the vasectomized male partner should be the sole partner for that subject.
- Combination of any two of the following (a+b or a+c, or b+c):
  a. Use of oral, injected or implanted hormonal methods of contraception or other forms of hormonal contraception that have comparable efficacy (failure rate <1%), for example hormone vaginal ring or transdermal hormone contraception.
  b. Placement of an intrauterine device (IUD) or intrauterine system (IUS)
  c. Barrier methods of contraception: Condom or Occlusive cap (diaphragm or cervical/vault caps) with spermicidal foam/gel/film/cream/vaginal suppository

In case of use of oral contraception women should have been stable on the same pill for a minimum of 3 months before taking study treatment.

Part A (MRA)

17. Contraindication to terlipressin acetate.
18. Body mass index (BMI) (weight[kg] / height[m]^2) > 40 kg/m^2.
19. Any contraindication to having an MRI scan including:
   - Brain aneurysm clip
   - Implanted neural stimulator
   - Implanted cardiac pacemaker, pacemaker wires or defibrillator
   - Prosthetic heart valves
   - Cochlear implant
   - Ocular foreign body (e.g. metal shavings)
   - Other implanted medical devices (e.g. Swan Ganz catheter)
   - Implanted insulin pump
   - Metal shrapnel or bullet
   - Severe claustrophobia
   - Tattoos (at the discretion of the local imager)
   - Any additional contraindications to MRI at the local facility that will perform the test

Part B (direct venous pressure measurement)

20. Contraindication to catheterization (e.g. portal vein thrombosis, overt hepatic encephalopathy, severe cardiac or pulmonary disease).

5 Treatment

5.1 Investigational and control treatment

The sponsor will provide the following open label bulk medication for Part A:

- Glypressin® commercial pack, each ampoule contains 1mg of terlipressin acetate in 8.5mL solution for injection
- Serelaxin (RLX030) solution 3.5mg/ 3.5mL per vial (1.0mg/mL)

The sponsor will provide the following open label study medication for Part B:

- Serelaxin (RLX030) solution 3.5mg/3.5mL per vial (1.0mg/mL)

5.2 Treatment arms

Part A (MRA)

40 patients will be assigned to one of the following two treatment arms in a ratio of 1:1:

- Terlipressin (2mg i.v. bolus)
- Serelaxin (RLX030) (i.v. infusion: 80 µg/kg/d for 60 min followed by 30 µg/kg/d for at least 60 min)

Part B (direct venous pressure measurement)

6 patients will be enrolled into the following treatment arm:

- Serelaxin (RLX030) (i.v. infusion: 80 µg/kg/d for 60 min followed by 30 µg/kg/d for at least 60 min)

5.3 Treatment assignment

For part A only, treatment allocation cards will be utilized for treatment group assignment (part B will not require the use of such cards). One set of treatment allocation cards with unique randomization numbers printed on them and covered by a removable, scratch-off covering will be provided for use at the clinical site.

The investigator or his/her delegate will scratch-off the covering of the treatment allocation card with the lowest available, unassigned randomization number thus revealing the treatment group the patient is assigned to after confirming that the patient fulfills all the inclusion/exclusion criteria for part A.

The patient randomization list will be produced by Novartis Drug Supply Management using a validated system that automates the random assignment of randomization numbers to the two different treatment groups. These randomization numbers are linked to the different treatment arms.

The randomization scheme for patients will be reviewed and approved by a member of the Biostatistics Quality Assurance Group.

5.4 Treatment blinding

Open label supplies will be used as this is an open label study. Treatment allocation cards will be provided to reveal the treatment each patient has been randomly assigned to receive.

5.5 Treating the patient

5.5.1 Patient numbering

Each patient is uniquely identified in the study by a combination of his/her center number and patient number. The center number is assigned by Novartis to the investigative site. Upon signing the informed consent form, the patient is assigned a patient number by the investigator. At each site, the first patient is assigned patient number 1, and subsequent patients are assigned consecutive numbers (e.g. the second patient is assigned patient number 2, the third patient is assigned patient number 3). Once assigned to a patient, a patient number will not be reused. If the patient fails to be randomized/assigned to treatment for any reason, the reason for not being randomized/assigned to treatment will be entered on the Screening Log, and the Demography eCRF should also be completed.

Re-screening of patients who failed screening is not allowed.

5.5.2 Dispensing the study treatment

Each study site will be supplied by Novartis with open-label bulk Serelaxin study drug for study part A2 and B. No unique medication numbers will be used. The primary and secondary packaging will have simple labels only.

Commercial Glypressin® product will be purchased and provided by Novartis and used for study part A1. Simple labels will be affixed to fulfil study drug labelling requirements.

Bulk medication labels will be in local language, will comply with the legal requirements of the country and will include storage conditions for the drug but not information about the subject.

Investigator staff will select the study drug according to the patient's treatment assigment and document this in the source documents. The randomization number must also be recorded on the Randomization Number eCRF (part A only).

5.5.3 Supply, storage and tracking of study treatment

Study treatment must be received by a designated person at the study site, handled and stored safely and properly, and kept in a secured location to which only the investigator and designated assistants have access. Upon receipt, all study drugs should be stored according to the instructions specified on the drug labels. Clinical supplies are to be dispensed only in accordance with the protocol.

Medication labels will be in the local language and comply with the legal requirements of each country. They will include storage conditions for the drug, but no information about the patient.

PPD [redacted]

The investigator must maintain an accurate record of the shipment and dispensing of study drug in a drug accountability ledger. Monitoring of drug accountability will be performed by the field monitor during site visits and at the completion of the trial. Patients will be asked to return all unused study drug and packaging at the end of the study or at the time of study drug discontinuation.

At the conclusion of the study, and as appropriate during the course of the study, the investigator will return all used and unused study drug, packaging, drug labels, and a copy of the completed drug accountability ledger to the Novartis monitor or to the Novartis address provided in the investigator folder at each site.

5.5.4 Instructions for administering study treatment

For Part A1: Terlipressin acetate will be administered via slow i.v. bolus injection of 2mg (over 3-5 min) just after completion of the first MRA assessment at visit 2.

For Part A2: Serelaxin will be administered via i.v. infusion at two different infusion rates: 80 µg/kg/day for 60 minutes followed by 30 µg/kg/day for at least 60 minutes (until completion of the second MRA assessement and the other 120 min assessments at visit 2).

For Part B: Serelaxin will be administered via i.v. infusion at two different infusion rates: 80 µg/kg/day for 60 minutes followed by 30 µg/kg/day for at least 60 minutes (until completion of the second inferior vena cava pressure (IVCP) measurement and the other 120 min assessments at visit 2).

For detailed instructions on preparation and administration of study drug, please refer to the pharmacy manual that will be provided separately.

All dosages administered to the patient and all dose changes during the study must be recorded on the Dosage Administration Record eCRF.

5.5.5 Permitted dose adjustments and interruptions of study treatment

Dose adjustments and/or interruptions of study treatment are not permitted.

Any study drug adjustment and/or interruption must be recorded on the Dosage Administration Record eCRF.

5.5.6 Rescue medication

Rescue medication to treat severe or serious condition in the opinion of the investigator is allowed. Use of rescue medication must be recorded on the Concomitant medications/Significant non-drug therapies after start of study drug eCRF.

5.5.7 Concomitant treatment

The investigator should instruct the patient to notify the study site about any new medications he/she takes after the start of the study drug. All medications and significant non-drug therapies (including physical therapy and blood transfusions) administered after the patient starts treatment with study drug must be listed on the Concomitant medications/Significant non-drug therapies after start of study drug eCRF.

5.5.8 Prohibited treatment

Use of the treatments displayed in Table 5-1 is **not** allowed within the specified time period. No washout of these prohibited drugs will be performed during or prior to screening with the intention to make a patient eligible for study participation.

Table 5-1 Prohibited treatment

Medication	Time window	Actions to be taken
Any drug to treat portal hypertension (e.g. vasodilators such as non-selective beta blockers or nitrates)	Within 1 month prior to screening (visit 1) until (and including) visit 2	- Treat as screen failure<br>- Don't administer study treatment / discontinue study treatment<br>- Record on the Concomitant medications/Significant non-drug therapies after start of study drug eCRF
Diuretics (incl. spironolactone)	From screening (visit 1) to visit 2	- Treat as screen failure<br>- Don't administer study treatment / discontinue study treatment<br>- Record on the Concomitant medications/Significant non-drug therapies after start of study drug eCRF

5.5.9 Discontinuation of study treatment and premature patient withdrawal

Patients may voluntarily withdraw from the study for any reason at any time. They may be considered withdrawn if they state an intention to withdraw, fail to return for visits, or become lost to follow-up for any other reason.

If premature withdrawal occurs for any reason, the investigator must make every effort to determine the primary reason for a patient's premature withdrawal from the study and record this information on the Study Completion eCRF.

The investigator should discontinue the study treatment for a given patient or withdraw the patient from study if, on balance, he/she believes that continuation would be detrimental to the patient's well-being.

Study treatment must be discontinued under the following circumstances:

- Withdrawal of informed consent
- Emergence of clinically significant adverse events at the discretion of the investigator
- Any other protocol deviation that results in a significant risk to the patient's safety

Signs or symptoms of hypotension or blood pressure less than either SBP <90 and/or DBP <50 mmHg should be thoroughly evaluated by the investigator and if clinically warranted, the patient must be permanently discontinued from study drug.

PPD [redacted]

Patients who discontinue study treatment should **not** be considered withdrawn from the study. A Study Drug Discontinuation form should be completed, giving the date and primary reason for stopping study treatment. See Section 6 for the required assessments of these patients after discontinuation of study treatment.

For patients who are lost to follow-up (i.e. those patients whose status is unclear because they fail to appear for study visits without stating an intention to withdraw), the investigator should show "due diligence" by documenting in the source documents steps taken to contact the patient, e.g. dates of telephone calls, registered letters, etc.

Patients who are prematurely withdrawn from the study will be replaced by an equal number of newly enrolled patients only if they are discontinued prior to the completion of the second MRA data acquisition period (part A) or if final PV and IVC pressure measurements at visit 2 are not made (part B).

5.5.10 Emergency unblinding of treatment assignment

Not applicable.

5.5.11 Study completion and post-study treatment

The study is considered completed for an individual patient after the patient's visit 3 has been completed. Patients already in screening when the planned enrolment is met and the last visit 2 has been successfully completed will not be enrolled/randomized/assigned to study treatment.

No post-study treatment will be provided by Novartis.

The investigator must provide follow-up medical care for all patients who are prematurely withdrawn from the study, or must refer them for appropriate ongoing care.

No recommendations for initiating other treatment outside the study are provided.

5.5.12 Early study termination

The study can be terminated at any time for any reason by Novartis. Should this be necessary, the patient should be seen as soon as possible and treated as described in Section 6 for a prematurely withdrawn patient. The investigator may be informed of additional procedures to be followed in order to ensure that adequate consideration is given to the protection of the patient's interests. The investigator will be responsible for informing IRBs and/or ECs of the early termination of the trial.

6 Visit schedule and assessments

Table 6-1 lists all of the assessments and indicates with an "X" the visits at which they will be performed (some of them apply only for part A or B or patients receiving serelaxin respectively).

Patients should be seen for all three visits on the designated day. However, falling outside this visit window will not constitute a protocol deviation. The investigator will bring the patient

back in for study visit as close as possible to recommended visit schedule. Documentation of attempts to contact the patient should be recorded in the patient's record.

Table 6-1 Assessment schedule

Visit		1	2				3 (EOS) and SDD/PPW
Day*	Category	-7*	1				29*
			0min**	1-119min**	120min**	Recovery **	
Obtain informed consent	S	X					
Check inclusion and exclusion criteria	S	X	X				
Demography and medical history	DS	X	X				
History of portal hypertension and cirrhosis and alcohol status	DS	X	X				
Concomitant medication	DS	X	X				X
Physical Exam[1]	S	X	X				X
Height	DS	X					
Weight	DS	X					
Vital signs (incl. blood pressure measurements)	DS	X	X	X[2]	X[2]	X[2]	X
Local ECG	DS	X					
Fluoroscopic monitoring to ensure correct position of catheter (part B only)	S		X		X		
Screening log	DS	X					
Pregnancy test[3]	DS	X	X				X
Blood sample collection for hematology (central laboratory)	DS	X					
Blood sample collection for clinical chemistry (central laboratory)	DS	X					X
Heart rate monitoring[4]	-		X	X	X	X	
Central ECG	DS		X[5]	X[5]	X[5]	X[5]	
Randomization via treatment allocation cards (part A only)	DS		X				
Serum sample for immunogenicity assessments (serelaxin antibodies) by central laboratory[6, 7]	DS		X				X
Serum sample for phamacokinetic assessments (serelaxin levels) by central laboratory[6, 7]	DS		X	X[8]	X	X	X
Peripheral blood collection for biomarker analysis by	DS		X		X		

Visit		1	2				3 (EOS) and SDD/PPW
Day*	Category	-7*	1				29*
			0min**	1-119min**	120min**	Recovery **	
central laboratory[6]							
Portal vein blood collection for biomarker analysis by central laboratory (part B only)	DS		X		X[9]		
Urine sample for biomarker analysis by central laboratory[6]	DS		X		X		
Study drug administration	DS			X			
Magnetic resonance angiography (part A only)	DS		X		X		
Direct portal vein pressure measurement (part B only)[10]	DS		X	X	X		
Direct right atrial and inferior vena cava pressure measurements (part B only)[10]	DS		X		X		
Serious adverse events	DS	X	X	X	X	X	X
Adverse events	DS		X	X	X	X	X
Study Completion form	DS						X

EOS = End of study
SDD/PPW = Study drug discontinuation/Premature patient withdrawal
S = assessment to be recorded on source documentation
DS = data to be recorded on source documentation and to be entered into the database

[1] A complete physical examination is required at visit 1 and 3 (EOS). A short physical examination is sufficient at visit 2.

[2] Blood pressure will be assessed approximately every 10 min until the completion of the MRA data acquisition/direct venous pressure measurement and approximately every 15 min until completion of the 60 min recovery period.

[3] Women of childbearing potential only. Local urine pregnancy tests will be performed. The visit 2 test is to be performed prior to randomization and study drug administration. In case of a positive urine pregnancy result a confirmatory serum pregnancy test has to be performed at the central laboratory.

[4] HR will be continuously monitored via ECG from the initiation of study drug administration to end of recovery.

[5] Printouts of central ECG recordings should be reviewed locally in a timely manner to ensure patient safety. The 1-119 min central ECG should be performed about 60 min after initiating study drug administration.

[6] Only in patients receiving serelaxin study drug.

[7] Additional blood samples may be collected for immunogenicity and accompanying serelaxin assessments as specified in Section 6.6.2.

[8] At 60 min just prior to decreasing the serelaxin infusion rate from 80 to 30 µg/kg/day.

[9] The 120 min portal vein blood sample will be collected between the final (120 min) direct PV and the final IVC pressure measurement.

[10] Prior to initiating the serelaxin infusion (0min) and at 15, 30, 45, 60, 90, 105 and 120 min after initiating the serelaxin infusion portal vein pressure will be measured. Prior to first (0 min) and after last (120 min) portal vein pressure (PVP) measurement the right atrial pressure (RAP) and the inferior vena cava pressure (IVCP) will be measured.

*The time windows between the visits are flexible (i.e. may be slightly shorter or longer than the suggested 6 or 28 days), but unnecessary delays should be avoided and treatment with study drug within one week after visit 1 should be targeted. Visit 1 central lab results must be received and reviewed by the sites prior to proceeding with

PPD [redacted]

Visit		1	2				3 (EOS) and SDD/PPW
Day*	Category	-7*	1				29*
			0min**	1-119min**	120min**	Recovery **	

visit 2.

**The timing refers to the initiation of study drug administration (0min = prior to study drug administration). The serelaxin infusion is to be continued beyond 120min until all assessments are completed except urine sample collection for biomarker analysis. The recovery period starts after all 120 min assessments are completed / serelaxin infusion is stopped and lasts for at least 60min. Sample collection planned for the recovery time point should be performed at the end of the 60 min recovery period.

6.1 Information to be collected on screening failures

Patients discontinuing prior to receiving study medication are considered screening failures. If a patient has withdrawn before entering the treatment phase, all Visit 1 eCRFs, including demographics, vital signs, portal hypertension and cirrhosis history, alcohol status, relevant medical history, and Screening Log entry with the primary reason for discontinuation should be completed. It is not necessary to complete all the required evaluations unless medically indicated.

6.2 Patient demographics/other baseline characteristics

Patient demographic and baseline characteristic data to be collected on all patients include: date of birth, age, sex, race, and ethnicity. Relevant medical history/current medical condition data includes data until the start of study drug. If possible, diagnoses and not symptoms will be recorded.

Demography/baseline characteristic information will be collected at Visit 1. All relevant medical history, portal hypertension and cirrhosis history and alcohol status will be collected at Visit 1 and reviewed at Visit 2 before administering any study drug.

6.3 Treatment exposure and compliance

All medications currently being taken, having been taken up to 30 days prior to entry into the study, and those taken after the start of study drug will be recorded on the Concomitant medications/Significant non-drug therapies eCRF.

Dosing information for study medication will be collected on corresponding dosage administration record eCRFs.

6.4 Efficacy

6.4.1 Part A: Blood flow measured via MRA

Haemodynamic data will be acquired using a 3.0 Tesla whole body Siemens Verio dedicated research MR scanner. A non-contrast MR angiography sequence (Siemens NATIVE TrueFISP (True Fast Imaging with Steady state Precession) will be performed to acquire phase contrast blood flow measurements from vessels of interest (right and left renal artery, hepatic artery, superior mesenteric artery, descending thoracic aorta, portal vein, descending

abdominal aorta, and azygos vein). For each vessel the velocity encoding of the acquisition will be adjusted on a patient-by-patient basis.

Scan sequences will be performed pre and post 120 minutes of serelaxin infusion or terlipressin acetate bolus administration. During scanning, all images will be checked visually after each flow measurement sequence; if motion artefacts are detected, the individual vessel scan will be repeated. Flow measurements will be derived from analysis of the phase contrast images using the Siemens Argus Flow software directly on the scanner workstation console. The total acquisition time for each MRA sequence is approximately 45-60 minutes (incl. repeats in case of motion artefacts).

On the vascular images where the phase contrast sequence has been applied for assessment of flow rates (hepatic artery, portal vein, superior mesenteric artery, descending thoracic aorta (acquired 2 cm above the coeliac trunk), inferior aorta (acquired 2 cm above the iliac bifurcation), renal arteries, azygos vein)), contours are drawn manually (or semi-automatically after seed points are indicated at the lumen boundary) at end-diastole (first image of the cine acquisition) and end-systole (smallest cavity size during cardiac cycle) on every slice. For the flow measurements, the lumen of each vessel will be carefully evaluated at the peak systolic phase image, and the area of the lumen will be used as the region of interest for the flow and velocity quantification program, yielding values that represent average volumetric flow rate and peak systolic velocity within the vessel (Barthelmes et al 2009).

For detailed instructions on MRA procedure, please refer to the MRI manual that will be provided separately.

6.4.2 Part B: Direct venous pressure measurement

Patients with cirrhosis and a TIPSS *in situ* will undergo portography at the time of routine shunt assessment at the site's X-ray department. Typically, an introducer sheath is inserted under local anaesthetic into the right internal jugular vein and a catheter inserted to measure inferior vena cava pressure (IVCP) and right atrial pressure (RAP) using fluoroscopic guidance. After catheterization of the TIPSS, shunt patency is determined by injection of iodinated contrast medium, followed by measurement of portal vein pressure (PVP) and calculation of the portal pressure gradient (PPG). A non-study specific informed consent form will be signed by the patients according to the site's standard practice for their portography procedure as performed for routine TIPSS assessment. After portography has confirmed a fully functioning shunt without variceal filling, and a PPG of >5mm Hg, the PV catheter will be secured in position and the patient transferred to the site's Clinical Research Facility. Fluoroscopic monitoring will be used to confirm that the catheter is still properly positioned in the PV and did not move during the transfer. The catheter position will be corrected if needed. Just prior to starting the serelaxin study drug infusion the pre-dosing PVP as measured at the site's X-ray department will be confirmed. The catheter will be kept in the PV, secured in place and not moved throughout the measurement period of 2 hours during which the PVP will be measured at 15 min intervals (i.e. prior to and at 15, 30, 45, 60, 75, 90, 105 and 120 min of serelaxin infusion). Before complete withdrawal of the catheter, a second IVCP and RAP reading will be obtained under fluoroscopic guidance.

PPD [redacted]

6.4.3 Appropriateness of efficacy assessments

PHT is the earliest and most important consequence of cirrhosis and underlies most of the clinical complications of the disease. Indirect measurement of PHT by the hepatic venous pressure gradient (HVPG) is the gold standard method for evaluating the presence and severity of PHT, but this technique is considered invasive and is not routinely performed in all centers. A reduction in HVPG (e.g. after drug therapy) below 12 mmHg or by >20% from baseline is associated with a significant reduction in complications and death (Ripoll 2007). Although a novel therapy for PHT would ultimately need to demonstrate efficacy in terms of HVPG reduction, one could argue that this approach is both too invasive and too restrictive for exploratory studies. HVPG captures only limited information with no indication of the relative influence of a drug on the various factors that affect PHT including hepatic vascular resistance (due to scar tissue and intrahepatic vasoconstriction) and extrahepatic haemodynamic abnormalities involving both the splanchnic and systemic circulations. Accordingly, we propose a unique strategy using complementary but distinct approaches to provide a comprehensive assessment of the haemodynamic response to serelaxin in patients with cirrhosis and PHT.

Magnetic resonance angiography

MRA is a reliable technique for the non-invasive assessment of blood flow changes in the body. MRA has previously been used to successfully investigate splanchnic blood flow (Barthelmes et al 2009) and present research efforts are focused on application of the MRA assessment to evaluate the 'splanchnic steal' phenomenon in patients with cirrhosis (McAvoy 2010). Through assessment of azygos flow, MRA also has been shown to accurately stage varices in patients with cirrhosis (Gouya et al 2011).

Using MRA, the effects of serelaxin on the hepatic, splanchnic, renal and systemic circulations will be evaluated. Taken together, these measurements will establish the potential for serelaxin as a therapy in cirrhosis (e.g. to reduce PHT in variceal bleeding and/or to increase renal blood flow in hepatorenal syndrome). Furthermore, as the approach utilizes a state of the art MR platform and an optimized non-contrast sequence, the risk of the MRI for study participants is an absolute minimum.

Direct venous pressure measurement

Although MRA enables a broad assessment of blood flow changes, it does not directly measure pressure. The HVPG is a good reflection of portal pressure in patients with alcoholic or viral cirrhosis, but it remains an invasive procedure. Furthermore, after the acute administration of a drug acting on the splanchnic circulation, the HVPG measurement does not necessarily provide a reliable estimation of the magnitude of the changes in the portal pressure (Valla et al 1984). Direct portal vein pressure measurement in patients with a TIPSS has been found to be reproducible with a coefficient of variation of <10% (Forrest et al 1996). Although the presence of a shunt alters intrahepatic haemodynamics by removing a large element of mechanical resistance, a significant dynamic resistive component remains. Patients with a TIPSS *in situ* undergo regular shunt assessment for patency and portal pressure monitoring as part of standard medical care. TIPSS therefore provides a valuable opportunity

to directly assess changes in portal pressure in patients with cirrhosis at the time of portal vein catheterization for routine portography.

6.5 Safety

6.5.1 Physical examination

A complete physical examination will be performed at screening and study completion (incl. SDD and/or PPW). It will include the examination of general appearance, heart, lung, abdomen, thyroid, and lymph nodes. If indicated, a more detailed exam will be performed.

A short physical examination will be performed at Visit 2 and include the examination of general appearance.

At Screening, height in centimeters (cm) and body weight (to the nearest 0.1 kilogram [kg] in indoor clothing, but without shoes) will be measured.

Information for all physical examinations must be included in the source documentation at the study site. Significant findings that are present prior to the start of study drug must be included in the Relevant Medical History/Current Medical Conditions screen on the patient's eCRF. Significant findings made after the start of study drug which meet the definition of an Adverse Event must be recorded on the Adverse Event screen of the patient's eCRF.

6.5.2 Vital signs

Vital signs include sitting blood pressure and pulse measurements and will be obtained at each visit until end of study.

At each study visit, after the patient has been sitting for five minutes, with back supported and both feet placed on the floor, systolic and diastolic blood pressure and pulse will be measured three times in 1-2 min intervals using an automated validated device, e.g. OMRON, with an appropriately sized cuff.

Additional single supine blood pressure readings will be taken at visit 2 in approximately 10 min intervals starting prior to the administration of study drug until the completion of all 120 min assessments.

Single blood pressure readings will be continued in 15 min intervals during the ≥60 min recovery period but in the sitting position.

The patient's dominant arm should be noted in source documents and used for all measurements while the non-dominant arm should be used for peripheral blood sample collections.

All measurements will be recorded in the patient's source documents and case report form (eCRF).

6.5.3 Local ECG

Standard 12 lead ECGs will be performed at screening (visit 1). Interpretation of the tracings must be made by a qualified physician and documented on the ECG section of the eCRF. Only clinically significant abnormalities should be reported on this page. Clinically significant abnormalities at screening should also be recorded on the relevant medical history/Current

medical conditions eCRF page. Each ECG tracing should be labeled with the study number, patient initials, patient number, date, and kept in the source documents at the study site.

6.5.4 Central ECG

The ECGs performed at the four time points of visit 2 will be collected and analysed centrally. For this purpose the study site will be provided with 12-lead ECG equipment. Triplicate ECGs will be performed in approximately 2 minute intervals for each of the four time points.

Printouts of central ECG tracings should also be analysed locally and significant findings made after the start of study drug which meet the definition of an Adverse Event must be recorded on the Adverse Event screen of the patient's eCRF. Each ECG tracing should be labeled with the study number, patient initials, patient number, date, and kept in the source documents at the study site.

For detailed instructions on central ECG procedures, please refer to the central ECG manual that will be provided separately.

6.5.5 Laboratory safety evaluations

A central laboratory will be used for analysis of all specimens collected except urine for pregnancy testing. All central laboratory safety results will be communicated to the investigators and the sponsor. Details on the collection, shipment of samples and reporting of results by the central laboratory will be provided to investigators in the laboratory manual.

Clinically notable laboratory findings are defined in Appendix 1.

6.5.5.1 Hematology

Hemoglobin, hematocrit, red blood cell count, white blood cell count with differential, and platelet count will be measured.

6.5.5.2 Clinical chemistry

Blood urea, creatinine, total bilirubin, direct and indirect bilirubin, glutamic oxaloacetic transaminase (SGOT)/aspartate aminotransferase (AST), glutamic pyruvic transaminase (SGPT)/alanine aminotransferase (ALT), alkaline phosphatase, γ-glutamyltransferase (GGT), sodium, potassium, calcium, total protein, albumin and prothrombin time (PT) will be measured.

Potassium, sodium and serum creatinine concentration will be used to determine eGFR according to the sMDRD formula and confirm the eligibility of the patient to participate in the study.

PT will be used to determine the international normalized ratio (INR) and to calculate the Child-Pugh score (screening).

6.5.5.3 Pregnancy

All pre-menopausal women who are not surgically sterile will have urine pregnancy tests performed locally. If positive, confirmatory serum pregnancy tests are to be performed

[redacted]

centrally. If pregnancy is confirmed at visit 1 or 2, the patient must be excluded from study participation and not be randomized/receive any study drug.

6.5.6 Appropriateness of safety measurements

Since terlipressin acetate is a safe, marketed drug and serelaxin was found to be safe in previous studies, only general measures of safety are to be implemented in the present study (i.e., physical examinations, vital signs, laboratory evaluations (incl. hematology and clinical chemistry), ECG, pregnancy tests, collection of adverse events and serious adverse events) and no specific additional measures will be implemented.

6.6 Other assessments

6.6.1 Biomarkers

Biomarker measurements will be obtained from peripheral blood and urine samples (part A and B) as well as portal vein blood samples (part B only). Biomarker samples will only be collected from patients who will receive serelaxin but not from patients who will receive terlipressin acetate. The selected biomarkers to be studied will be the ones believed to be relevant to the pathophysiology of portal hypertension, cirrhosis, renal function or mechanism of drug action. Thus analysis may include: markers of vasoreactivity such as endothelin, markers of renal function such as UACR, markers of oxidative stress such as isoprostanes, and/or markers of fibrosis such as matrix metalloproteinases or TIMPs. The list may be changed or expanded further, as more relevant or novel (non-genomic) biomarkers may be discovered during the course of this study.

Blood plasma and urine sample aliquots will be stored in a bio bank for potential post-hoc analysis.

For detailed instructions on handling, labeling and shipment of biomarker samples, please refer to the laboratory manual that will be provided separately.

6.6.2 Immunogenicity (anti-serelaxin antibodies and neutralizing activity)

All patients who will receive serelaxin will be monitored for anti-drug antibodies (ADAs) response at baseline (visit 2, to evaluate potential interference and/or pre-existing ADAs) and 4 weeks post treatment (visit 3, to evaluate the development of an ADA response). Should a patient develop an adverse event indicative of a hypersensitivity reaction, an additional blood sample may be taken from this patient during an unscheduled visit between visit 2 and 3 to evaluate ADAs and the accompanying serelaxin concentration. These patients will be followed to AE resolution.

Immunogenicity Blood collection and processing

Blood samples will be taken by either direct venipuncture or an indwelling cannula inserted in a forearm vein contra-lateral to the site of infusion. If an indwelling cannula is used, a 0.5 mL discard will be taken before the immunogenicity sample in case the indwelling cannula was flushed with saline. Serum separator tubes will be used to collect serum for the quantification of anti-serelaxin antibody levels.

For assessment of immunogenicity (i.e. presence of anti-serelaxin-antibodies), approximately 3 mL blood, will be drawn using a serum separator tube. Following collection, the sample will be allowed to clot for approximately 45 minutes at room temperature and then centrifuged at 3-5°C for 15 minutes at approximately 2000g or sufficient time and force to achieve a clear serum layer above the red cell clot. Post centrifugation, at least 1 mL (i.e. two times 0.5 mL) serum will be aliquoted into two freezer proof polypropylene screw-cap tubes and put on dry ice. The rest of the serum will be transferred into back-up tubes. The serum tubes will be frozen within 90 min of sample collection and kept below -70° C until shipment to the central laboratory.

For detailed instructions on handling, labeling and shipment of immunogenicity samples, please refer to the laboratory manual that will be provided separately.

Immunogenicity analytical methods

Anti-serelaxin antibodies will be evaluated in serum in a validated four-tiered assay approach. All samples are initially screened for potential immunogenicity in the screening assay. Any positive screen results are confirmed using an immunodepletion assay. If a sample immunodepletes it is considered confirmed positive and the sample moves to a third tier titration assay. Confirmed positive samples will also be tested for neutralization of serelaxin biological activity using a validated bioassay.

The detailed method descriptions of the Immunogenicity assays will be included in the corresponding bioanalytical data reports.

No immunogenicity test results will be communicated to the investigators.

6.6.3 Pharmacokinetics

This only applies to patients who will receive serelaxin.

PK Blood collection and processing

Blood samples will be taken by either direct venipuncture or an indwelling cannula inserted in forearm vein contra-lateral to the site of infusion. If an indwelling cannula is used, a 0.5 mL discard will be taken before the PK sample in case the indwelling cannula was flushed with saline. Serum separator tubes will be used to collect serum for the quantification of serelaxin levels.

For pharmacokinetic analysis of serelaxin, approximately 2 mL blood will be drawn into a serum separator tube. Following collection, the sample will be allowed to clot for approximately 45 minutes at room temperature and then centrifuged at 3-5° C for 15 minutes at approximately 2000g or sufficient time and force to achieve a clear serum layer above the red cell clot.

Post centrifugation, at least 0.5 mL serum will be immediately transferred into a freezer proof polypropylene screw-cap tube and put on dry ice. The rest of the serum will be transferred into a second tube as back-up. The serum tubes will be frozen within 90 min of sample collection and kept below -70° C until shipment to the central laboratory.

For detailed instructions on handling, labeling and shipment of PK samples, please refer to the laboratory manual that will be provided separately.

Pharmacokinetic analytical methods

Serelaxin will be determined in serum by a validated Enzyme Linked ImmunoSorbent Assay (ELISA) based upon the commercially available kit from R&D Systems (Catalogue No. DRL200). The ELISA method uses a monoclonal antibody specific for relaxin-2 as the capture reagent and an enzyme-linked polyclonal antibody specific for relaxin-2 as the detection reagent.

The detailed method descriptions of the PK assays will be included in the corresponding bioanalytical data reports.

PK results will not be communicated to the investigators.

7 Safety monitoring

7.1 Adverse events

An adverse event is the appearance or worsening of any undesirable sign, symptom, or medical condition occurring after starting the study drug even if the event is not considered to be related to study drug. Study drug includes the investigational drug under evaluation and the comparator drug or placebo that is given during any phase of the study. Medical conditions/diseases present before starting study drug are only considered adverse events if they worsen after starting study drug. Abnormal laboratory values or test results constitute adverse events only if they induce clinical signs or symptoms, are considered clinically significant, or require therapy.

The occurrence of adverse events should be sought by non-directive questioning of the patient at each visit during the study. Adverse events also may be detected when they are volunteered by the patient during or between visits or through physical examination, laboratory test, or other assessments. All adverse events must be recorded on the Adverse Events eCRF with the following information:

1. the severity grade (mild, moderate, severe)
2. its relationship to the study drug(s) (suspected/not suspected)
3. its duration (start and end dates or if continuing at final exam)
4. whether it constitutes a serious adverse event (SAE)

An SAE is defined as an event which:

- is fatal or life-threatening
- results in persistent or significant disability/incapacity
- constitutes a congenital anomaly/birth defect
- requires inpatient hospitalization or prolongation of existing hospitalization, unless

hospitalization is for:

- routine treatment or monitoring of the studied indication, not associated with any deterioration in condition
- elective or pre-planned treatment for a pre-existing condition that is unrelated to the indication under study and has not worsened since the start of study drug
- treatment on an emergency outpatient basis for an event not fulfilling any of the definitions of a SAE given above and not resulting in hospital admission
- social reasons and respite care in the absence of any deterioration in the patient's general condition

- is medically significant, i.e. defined as an event that jeopardizes the patient or may require medical or surgical intervention to prevent one of the outcomes listed above

All malignant neoplasms will be assessed as serious under "medically significant" if other seriousness criteria are not met.

Unlike routine safety assessments, SAEs are monitored continuously and have special reporting requirements; see Section 7.2.

All adverse events should be treated appropriately. Treatment may include one or more of the following: no action taken (i.e. further observation only); study drug dosage adjusted/temporarily interrupted; study drug permanently discontinued due to this adverse event; concomitant medication given; non-drug therapy given; patient hospitalized/patient's hospitalization prolonged. The action taken to treat the adverse event should be recorded on the Adverse Event eCRF.

Once an adverse event is detected, it should be followed until its resolution or until it is judged to be permanent, and assessment should be made at each visit (or more frequently, if necessary) of any changes in severity, the suspected relationship to the study drug, the interventions required to treat it, and the outcome.

Should a patient develop an adverse event indicative of a hypersensitivity reaction, an additional blood sample may be taken from this patient during an unscheduled visit between visit 2 and 3 to evaluate ADAs and the accompanying serelaxin concentration. The patient will be followed to AE resolution.

Information about common side effects already known about the investigational drug can be found in the Investigator Brochure (IB) or will be communicated between IB updates in the form of Investigator Notifications. This information will be included in the patient informed consent and should be discussed with the patient during the study as needed.

7.2 Serious adverse event reporting

To ensure patient safety, every SAE, regardless of suspected causality, occurring after the patient has provided informed consent and until 30 days after the patient has stopped study participation (defined as time of last dose of study drug taken or last visit whichever is later).must be reported to Novartis within 24 hours of learning of its occurrence.

PPD [redacted]

Any SAEs experienced after this 30 day period should only be reported to Novartis if the investigator suspects a causal relationship to the study drug.

Recurrent episodes, complications, or progression of the initial SAE must be reported as follow-up to the original episode, regardless of when the event occurs. This report must be submitted within 24 hours of the investigator receiving the follow-up information. An SAE that is considered completely unrelated to a previously reported one should be reported separately as a new event.

Information about all SAEs is collected and recorded on the Serious Adverse Event Report Form. The investigator must assess the relationship of any SAE to study drug, complete the SAE Report Form in English, and send the completed, signed form by fax within 24 hours to the local Novartis Drug Safety and Epidemiology Department. The telephone and telecopy number of the contact persons in the local department of Clinical Safety and Epidemiology, specific to the site, are listed in the investigator folder provided to each site. The original copy of the SAE Report Form and the fax confirmation sheet must be kept with the case report form documentation at the study site.

Follow-up information is sent to the same person to whom the original SAE Report Form was sent, using a new SAE Report Form stating that this is a follow-up to the previously reported SAE and giving the date of the original report. The follow-up information should describe whether the event has resolved or continues, if and how it was treated, whether the blind was broken or not, and whether the patient continued or withdrew from study participation.

If the SAE is not previously documented in the Investigator's Brochure or Package Insert (new occurrence) and is thought to be related to the Novartis study drug, a Drug Safety and Epidemiology Department associate may urgently require further information from the investigator for Health Authority reporting. Novartis may need to issue an Investigator Notification (IN) to inform all investigators involved in any study with the same drug that this SAE has been reported. Suspected Unexpected Serious Adverse Reactions (SUSARs) will be collected and reported to the competent authorities and relevant ethics committees in accordance with Directive 2001/20/EC or as per national regulatory requirements in participating countries.

7.3 Liver safety monitoring

To ensure patient safety and enhance reliability in determining the hepatotoxic potential of an investigational drug, a standardized process for identification, monitoring and evaluation of liver events has to be followed.

Liver events are divided into two categories:

- Liver events of special interest (AESI) which consist of LFTs elevations
- Medically significant liver events which are considered as serious adverse events (SAEs) and which consist of marked elevations of LFTs and / or pre-specified adverse events.

Please refer to Table 14-1 in Appendix 2 for complete definitions of liver events.

Any liver event which meets the criteria for "**medically significant**" event as outlined in Table 14-1 of Appendix 2 should follow the **standard procedures for SAE reporting** as described in Section 7.2.

Every liver event as defined in Table 14-1 of Appendix 2 should be followed up by the investigator or designated personal at the trial site as summarized below. Detailed information is outlined in Table 14-2 of Appendix 2.

- Repeating the LFT to confirm elevation as appropriate
- Discontinuation of the investigational drug if appropriate
- Hospitalization of the patient if appropriate
- A causality assessment of the liver event via exclusion of alternative causes (e.g., disease, co-medications)
- An investigation of the liver event which needs to be followed until resolution.

These investigations can include serology tests, imaging and pathology assessments, hepatologist's consultancy, based on investigator's discretion. All follow-up information, and the procedures performed should be recorded on appropriate eCRF pages, including the liver event overview eCRF pages.

7.4 Pregnancy reporting

To ensure patient safety, each pregnancy occurring while the patient is in the study must be reported to Novartis within 24 hours of learning of its occurrence. The pregnancy should be followed up to determine outcome, including spontaneous or voluntary termination, details of the birth, and the presence or absence of any birth defects, congenital abnormalities, or maternal and/or newborn complications.

Pregnancy should be recorded on a Clinical Trial Pregnancy Form and reported by the investigator to the local Novartis Drug Safety and Epidemiology Department. Pregnancy follow-up should be recorded on the same form and should include an assessment of the possible relationship to the study treatment.

Any SAE experienced during pregnancy must be reported on the SAE Report Form.

7.5 Data Monitoring Committee

There will be no data monitoring committee for this study.

8 Data review and database management

8.1 Site monitoring

Before study initiation, at a site initiation visit or at an investigator's meeting, a Novartis representative will review the protocol and eCRFs with the investigators and their staff. During the study, the field monitor will visit the site regularly to check the completeness of patient records, the accuracy of entries on the eCRFs, the adherence to the protocol and to Good Clinical Practice, the progress of enrollment, and to ensure that study drug is being stored, dispensed, and accounted for according to specifications. Key study personnel must be available to assist the field monitor during these visits.

The investigator must maintain source documents for each patient in the study, consisting of case and visit notes (hospital or clinic medical records) containing demographic and medical

information, laboratory data, electrocardiograms, and the results of any other tests or assessments. All information on eCRFs must be traceable to these source documents in the patient's file. The investigator must also keep the original informed consent form signed by the patient (a signed copy is given to the patient).

The investigator must give the monitor access to all relevant source documents to confirm their consistency with the eCRF entries. Novartis monitoring standards require full verification for the presence of informed consent, adherence to the inclusion/exclusion criteria, documentation of SAEs, and the recording of data that will be used for all primary and safety variables. Additional checks of the consistency of the source data with the eCRFs are performed according to the study-specific monitoring plan. No information in source documents about the identity of the patients will be disclosed.

8.2 Data collection

Designated investigator staff will enter the data required by the protocol into the Electronic Case Report Forms using fully validated software that conforms to 21 CFR Part 11 requirements. Designated investigator site staff will not be given access to the EDC system until they have been trained. Automatic validation programs check for data discrepancies and, by generating appropriate error messages, allow the data to be confirmed or corrected before transfer of the data to the contract research organization (CRO) working on behalf of Novartis. The Investigator must certify that the data entered into the Electronic Case Report Forms are complete and accurate. After database lock, the investigator will receive a CD-ROM or paper copies of the patient data for archiving at the investigational site.

8.3 Database management and quality control

A CRO working on behalf of Novartis will review the data entered into the eCRFs by investigational staff for completeness and accuracy and instruct the site personnel to make any required corrections or additions. Queries are sent to the investigational site using an electronic data query. Designated investigator site staff is required to respond to the query and confirm or correct the data. If the electronic query system is not used, a paper Data Query Form will be faxed to the site. Site personnel will complete and sign the faxed copy and fax it back to Novartis staff who will make the correction to the database. The signed copy of the Data Query Form is kept at the investigator site.

Concomitant medications entered into the database will be coded using the WHO Drug Reference List, which employs the Anatomical Therapeutic Chemical classification system. Medical history/current medical conditions and adverse events will be coded using the Medical dictionary for regulatory activities (MedDRA) terminology.

Laboratory samples will be processed centrally and the results will be sent electronically to a designated CRO.

ECG readings will be processed centrally and the results will be sent electronically to a designated CRO.

MRA data will be reviewed by the site staff with quantitative image analysis captured in the eCRF. Additionally, a central imaging laboratory will perform a more detailed, automated,

algorithm-based quantitative image analysis that is reader independent. These analysis results will be sent electronically to a designated CRO.

9 Data analysis

Summary statistics for continuous variables will include N, mean, standard deviation, minimum, lower quartile, median, upper quartile, maximum. Summary statistics for discrete variables will be presented in contingency tables and will include absolute frequencies.

If not otherwise specified, p-values will be presented as two-sided p-values and two-sided confidence intervals will be displayed. All hypothesis tests will be for within group comparisons.

9.1 Analysis sets

Part A (MRA)

Randomized set: The randomized set will be defined as all subjects who were randomized.

Treated set: The treated set includes all subjects who received any amount of study treatment during the treatment period and had at least one post-baseline assessment during that period. Subjects will be analyzed according to treatment received. This will be the primary analysis set for the study.

Per-protocol set: The per protocol set includes all patients in the treated set who received study medication as specified in the protocol and who did not have any major protocol deviations. Subjects will be analyzed according to treatment received.

Part B (direct venous pressure measurement)

Randomized set: there is no randomization for this part of the study, so a randomized set will not be defined.

Entered set: The entered set will be defined as all subjects who entered the study (i.e. signed the informed consent form) and had baseline measurements made.

Treated set: The treated set includes all subjects who received any amount of study treatment during the treatment period and had at least one post-baseline assessment during that period. This will be the primary analysis set for the study.

Per-protocol set: The per protocol set includes all patients in the treated set who received study medication as specified in the protocol and who did not have any major protocol deviations.

9.2 Patient demographics and other baseline characteristics

9.2.1 Demographics and baseline characteristics

Part A (MRA)

Summary statistics will be presented for continuous demographic and baseline characteristic variables for each treatment group and for all subjects in the randomized set. The number and percentage of subjects in each category will be presented for categorical variables for each treatment group and all subjects.

Part B (direct venous pressure measurement)

Summary statistics will be presented for continuous demographic and baseline characteristic variables for all subjects in the entered set. The number and percentage of subjects in each category will be presented for categorical variables for all subjects.

9.2.2 Medical history

Part A (MRA)

For all patients in the randomized set, any condition entered as medical history or current medical conditions at baseline will be summarized by treatment group using the system organ class and preferred term of the MedDRA dictionary. Summaries of portal hypertension and cirrhosis specific medical history and alcohol status will also be provided by treatment group for all patients in the randomized set.

Part B (direct venous pressure measurement)

For all patients in the entered set, any condition entered as medical history or current medical conditions at baseline will be summarized using the system organ class and preferred term of the MedDRA dictionary. Summaries of portal hypertension and cirrhosis specific medical history and alcohol status will also be provided for all patients in the entered set.

9.3 Treatments (study drug, rescue medication, other concomitant therapies, compliance)

9.3.1 Study treatment

Part A (MRA)

The analysis of study treatment data will be based on the treated set.

A summary of patients receiving the planned dose regimen of study medication and reasons for not receiving planned serelaxin dose will be presented by day and infusion period. Reason for not receiving planned terlipressin acetate bolus injection will be summarized at a single time point. For the serelaxin group, there will be two infusion periods, the first 60 minutes of infusion and the second at least 60 minutes of infusion (until the 120 min assessment procedure is completed).

PPD [redacted]

Part B (direct venous pressure measurement)

The analysis of study treatment data will be based on the treated set.

A summary of patients receiving the planned dose regimen of serelaxin study medication and reasons for not receiving planned serelaxin dose regimen will be presented by day and infusion period. There will be two infusion periods, the first 60 minutes of infusion and the second at least 60 minutes of infusion (until 120 min assessment procedure is completed).

9.3.2 Prior and concomitant treatment

Part A (MRA)

Prior and concomitant treatments will be summarized by treatment group in separate tables.

Prior treatments are defined as treatments taken prior to first dose of study treatment. Any treatment given at least once between the day of first dose of randomized study treatment and the last day of study visit will be a concomitant treatment, including those which were started pre-baseline and continued into the treatment period.

Treatments will be presented in alphabetical order, by Anatomical Therapeutic Classification (ATC) codes and grouped by anatomical main group. Tables will also show the overall number and percentage of subjects receiving at least one treatment of a particular ATC code and at least one treatment in a particular anatomical main group.

In addition, non-drug therapies will be summarized.

Part B (direct venous pressure measurement)

Prior and concomitant treatments will be summarized in separate tables.

Prior treatments are defined as treatments taken prior to first dose of study treatment. Any treatment given at least once between the day of first dose of randomized study treatment and the last day of study visit will be a concomitant treatment, including those which were started pre-baseline and continued into the treatment period.

Treatments will be presented in alphabetical order, by Anatomical Therapeutic Classification (ATC) codes and grouped by anatomical main group. Tables will also show the overall number and percentage of subjects receiving at least one treatment of a particular ATC code and at least one treatment in a particular anatomical main group.

In addition, non-drug therapies will be summarized.

9.4 Analysis of the primary variable(s)

Part A (MRA)

No statistical hypothesis tests will be performed comparing the terlipressin acetate and serelaxin groups. Only within group comparisons are performed for this study.

All efficacy analyses will be performed on the treated set.

PPD [redacted]

Part B (direct venous pressure measurement)

All efficacy analyses will be performed on the treated set.

9.4.1 Variable

Part A (MRA)

Change from baseline of the blood flow for the total renal arteries (where the flow is the average flow over the cardiac cycle).

Total renal artery flow = left renal artery flow + right renal artery flow

Part B (direct venous pressure measurement)

Change from baseline of the portal pressure gradient

PPG = PVP-IVCP

9.4.2 Statistical model, hypothesis, and method of analysis

Part A (MRA)

Summary statistics (n, mean, SD, median, quartiles, min, max, geometric mean) for the baseline, post baseline and change from baseline measurements for the blood flow parameter for patients in the serelaxin group will be presented. Confidence intervals on the change from baseline for the blood flow parameter will be calculated for both the arithmetic and geometric means.

Data analysis for the terlipressin acetate group will be analyzed in a similar manner as the serelaxin group.

Part B (direct venous pressure measurement)

Summary statistics (n, mean, SD, median, quartiles, min, max, geometric mean) for the baseline, post baseline and change from baseline measurements for each parameter will be presented. Confidence intervals on the change from baseline for the parameters will be calculated for both the arithmetic and geometric means.

9.4.3 Handling of missing values/censoring/discontinuations

Missing data as a result of patients not having the post baseline measurement will not be imputed. Patients with missing post baseline data will be excluded from the analysis at that time point.

9.4.4 Supportive analyses

Part A (MRA)

The primary analysis will be repeated using the per-protocol set.

Part B (direct venous pressure measurement)

The primary analysis will be repeated using the per-protocol set.

9.5 Analysis of secondary variables

9.5.1 Key secondary variables

Not applicable.

9.5.2 Efficacy variables

Part A (MRA)

Change from baseline of the blood flows for the following blood vessels (where the flow is the average flow over the cardiac cycle):

- Hepatic artery
- Superior mesenteric artery
- Descending thoracic aorta
- Portal vein

Exploratory

Change from baseline of the blood flows/flow parameters for the following blood vessels (where the flow is the average flow over the cardiac cycle):

- Total liver = average portal vein flow + average hepatic artery flow
- Hepatic artery : portal vein ratio
- Descending abdominal aorta
- Left renal artery
- Right renal artery
- Azygos vein

The secondary and exploratory efficacy parameters will be analyzed in the same manner as the primary efficacy parameter.

Part B (direct venous pressure measurement)

Change from baseline of the following parameter:

- Portal vein pressure (PVP)

The secondary efficacy parameter will be analyzed in the same manner as the primary efficacy parameter.

Exploratory

Time from start of infusion to the beginning of the reduction in portal vein pressure.

9.5.3 Safety variables

All safety evaluations will be performed on the Treated set.

Adverse events

Part A (MRA)

Only treatment emergent adverse events (events started after the first dose of study treatment or events present prior to the first dose of study treatment but increased in severity based on preferred term) will be summarized.

AEs will be summarized by presenting, for each treatment group, the number and percentage of subjects having any AE, having an AE in each primary system organ class and having each individual AE (preferred term). Summaries will also be presented for AEs by severity and for study treatment related AEs. If a subject reported more than one adverse event with the same preferred term, the adverse event with the greatest severity will be presented. If a subject reported more than one adverse event within the same primary system organ class, the subject will be counted only once with the greatest severity at the system organ class level, where applicable.

Additional summaries will be provided for death, serious adverse event, other significant adverse events leading to discontinuation and adverse events leading to dose adjustment (including study treatment discontinuation).

Part B (direct venous pressure measurement)

Only treatment emergent adverse events (events started after the first dose of study treatment or events present prior to the first dose of study treatment but increased in severity based on preferred term) will be summarized.

AEs will be summarized by presenting, the number and percentage of subjects having any AE, having an AE in each primary system organ class and having each individual AE (preferred term). Summaries will also be presented for AEs by severity and for study treatment related AEs. If a subject reported more than one adverse event with the same preferred term, the adverse event with the greatest severity will be presented. If a subject reported more than one adverse event within the same primary system organ class, the subject will be counted only once with the greatest severity at the system organ class level, where applicable.

Additional summaries will be provided for death, serious adverse event, other significant adverse events leading to discontinuation and adverse events leading to dose adjustment (including study treatment discontinuation).

Laboratory data

Part A (MRA)

The summary of laboratory evaluations will be presented for two groups of laboratory tests (hematology and serum chemistry). Descriptive summary statistics for the change from baseline to each study visit will be presented. These descriptive summaries will be presented

by test group, laboratory test and treatment group. Change from baseline will only be summarized for subjects with both baseline and post baseline.

For each parameter, the maximum change from baseline within each study period will be analyzed analogously.

In addition, shift tables will be provided for all parameters to compare a subject's baseline laboratory evaluation relative to the visit's observed value. For the shift tables, the normal laboratory ranges will be used to evaluate whether a particular laboratory test value was normal, low, or high for each visit value relative to whether or not the baseline value was normal, low, or high. These summaries will be presented by laboratory test and treatment group. Shifts will be presented for most extreme values post-baseline.

Part B (direct venous pressure measurement)

The summary of laboratory evaluations will be presented for two groups of laboratory tests (hematology and serum chemistry). Descriptive summary statistics for the change from baseline to each study visit will be presented. These descriptive summaries will be presented by test group, laboratory test and treatment group. Change from baseline will only be summarized for subjects with both baseline and post baseline.

For each parameter, the maximum change from baseline within each study period will be analyzed analogously.

In addition, shift tables will be provided for all parameters to compare a subject's baseline laboratory evaluation relative to the visit's observed value. For the shift tables, the normal laboratory ranges will be used to evaluate whether a particular laboratory test value was normal, low, or high for each visit value relative to whether or not the baseline value was normal, low, or high. These summaries will be presented by laboratory test and treatment group. Shifts will be presented for most extreme values post-baseline.

Vital signs

Part A (MRA)

Analysis of the vital sign measurements using summary statistics for the change from baseline for each post-baseline visit will be performed. These descriptive summaries will be presented by vital sign, time point and treatment group. Change from baseline will only be summarized for subjects with both baseline and post-baseline values.

Part B (direct venous pressure measurement)

Analysis of the vital sign measurements using summary statistics for the change from baseline for each post-baseline visit will be performed. These descriptive summaries will be presented by vital sign, and time point. Change from baseline will only be summarized for subjects with both baseline and post-baseline values.

ECG

The following quantitative variables will be summarized: ventricular rate, PR interval, QRS duration, QT interval, and corrected QT interval (QTc) at each measurement. QT data will be analyzed with the Fridericia (primary) correction.

QTc will summarized by computing the number and percentage of subjects with:

- QTcF > 500 msec not present at baseline (new onset)
- QTcF > 480 msec not present at baseline (new onset)
- QTcF > 450 msec not present at baseline (new onset)
- QTcF > 470 msec for males and > 480 msec for females, not present at baseline (new onset)
- QTcF changes from baseline > 30 msec
- QTcF changes from baseline > 60 msec
- QRS change from baseline: more than 25% increase leading to QRS > 120 msec.
- Sinus pause > 3 sec
- PR > 250 msec not present at baseline (new onset)
- PR change from baseline: more than 25% increase leading to PR > 200 msec.
- Heart rate changes reflecting a more than 25% decrease from baseline to a heart rate < 40 beats per minute or a more than 25% increase from baseline reflecting a heart rate > 100 beats per minute.

In addition, shift tables comparing baseline ECG results (normal, abnormal, not available, total) with the maximum on-study result (normal, abnormal, not available, total) will be provided for each variable.

A listing of all newly occurring or worsening abnormalities will be provided, as well as a by-subject listing of all quantitative ECG parameters.

9.5.4 Resource utilization

Not applicable.

9.5.5 Health-related Quality of Life

Not applicable.

9.5.6 Pharmacokinetics

Only sparse samples will be taken for pharmacokinetic evaluations of serelaxin, therefore pharmacokinetic parameters such as AUC or CL will not be calculated for this study. Serelaxin serum concentration data will be listed as described below. Summary statistics will include mean (arithmetic and geometric), SD, CV (arithmetic and geometric), median, minimum and maximum. Concentrations below LLOQ will be treated as zero in summary statistics. A geometric mean will not be reported if the dataset includes zero values.

PPD [redacted]

Part A (MRA)

Summary statistics for baseline, each post-baseline measurement time point and change from baseline to each time point will be provided for subjects in the serelaxin group.

Part B (direct venous pressure measurement)

Summary statistics for baseline, each post-baseline measurement time point and change from baseline to each time point will be provided.

9.5.7 Pharmacogenetics/pharmacogenomics

Not applicable.

9.5.8 Biomarkers

Part A (MRA)

For each biomarker parameter measured, summary statistics for baseline, each post-baseline visit and change from baseline to each visit will be provided for patients receiving serelaxin. Summary statistics will include geometric mean and corresponding 95% confidence intervals. Exploratory analyses will be used to investigate the relationship between biomarkers and effects on selected cardiovascular and hemodynamic parameters.

Part B (direct venous pressure measurement)

For each biomarker parameter measured, summary statistics for baseline, each post-baseline visit and change from baseline to each visit will be provided for patients receiving serelaxin. Summary statistics will include geometric mean and corresponding 95% confidence intervals. Exploratory analyses will be used to investigate the relationship between biomarkers and effects on selected cardiovascular and hemodynamic parameters.

9.5.9 Immunogenicity

Part A (MRA)

Anti-serelaxin antibodies will be measured in serum samples collected during patient follow-up visit. To account for individual variability in background of each subject and to check for endogenous interference and/or pre-existing anti-serelaxin antibodies, anti-serelaxin antibodies will also be measured prior to dosing. Listings for immunogenicity results will be presented for every subject. Summary statistics including frequencies and percentages will be provided for occurrence of anti-serelaxin antibodies at each time point per treatment group.

Part B (direct venous pressure measurement)

Anti-serelaxin antibodies will be measured in serum samples collected during patient follow-up visit. To account for individual variability in background of each subject and to check for endogenous interference and/or pre-existing anti-serelaxin antibodies, anti-serelaxin antibodies will also be measured prior to dosing. Listings for immunogenicity results will be

presented for every subject. Summary statistics including frequencies and percentages will be provided for occurrence of anti-serelaxin antibodies at each time point per treatment group.

9.6 Sample size calculation

Part A (MRA)

This is an exploratory study. As such the study is not sized based on the power to detect a difference between treatment groups. Instead, the sample size is based on the required number of patients to identify by means of a 90% confidence interval a mean change from baseline of 25% or larger for each of the blood flow parameters. An increase of 25% is used, as this was identified as the indication of a clinically significant change in blood flow parameter that would be important to detect.

Table 9-1 contains the estimated mean and SD for each baseline blood flow parameter. The SD for the difference is estimated assuming a 10% correlation between baseline and post baseline measurements.

A sample size of 20 patients per group will be sufficient to create a 90% confidence interval on the mean change from baseline that will exclude the zero if a 25% increase is observed for that parameter, assuming the observed baseline mean and standard deviation for the study is similar to the historically observed standard deviation.

Table 9-1 Estimated number of patients required for the 90% CI on mean change from baseline to exclude zero

Parameter	Units	Mean	SD	Estimated SD for difference	Number of patients required per group*
Total renal arteries	L/min	0.62	0.24	0.322	14
Hepatic artery	L/min	0.27	0.12	0.161	18
Superior mesenteric artery	L/min	0.15	0.05	0.067	11
Descending thoracic aorta	L/min	3.32	0.81	1.087	7

*Assumes a 25% increase in mean blood flow is observed

Part B (direct venous pressure measurement)

This is an exploratory study. As such the study is not sized based on the power to detect a difference between treatment groups. Instead, the sample size is based on the required number of patients to identify by means of a 90% confidence interval a mean change from baseline of 25% or larger for each of the blood flow parameters. An increase of 25% is used, as this was identified as the indication of a clinically significant change in blood flow parameter that would be important to detect.

Table 9-2 contains the estimated mean and SD for each baseline blood flow parameter. The SD for the difference is estimated assuming a 10% correlation between baseline and post baseline measurements.

A sample size of 6 patients will be sufficient to create a 90% confidence interval on the mean change from baseline that will exclude the zero if a 25% increase is observed for that

parameter, assuming the observed baseline mean and standard deviation for the study is similar to the historically observed standard deviation.

Table 9-2 Estimated number of patients required for the 90% CI on mean change from baseline to exclude zero

Parameter	Units	Mean	SD	Estimated SD for difference	Number of patients required**
PPG Porto-atrial pressure gradient (PVP-RAP)	mmHg	9.30	1.60	2.147	5
Portal venous pressure (PVP)	mmHg	12.50	1.50	2.012	4
Right atrial pressure (RAP)*	mmHg	3.40	0.70	1.087	6

*RAP is very similar to inferior vena cava pressure (IVCP) as measured in this study for primary efficacy analysis.

**Assumes a 25% increase in mean pressure is observed

9.7 Power for analysis of key secondary variables

Not applicable.

9.8 Interim analyses

There will be no interim analyses for this study.

10 Ethical considerations

10.1 Regulatory and ethical compliance

This clinical study was designed and shall be implemented and reported in accordance with the ICH Harmonized Tripartite Guidelines for Good Clinical Practice, with applicable local regulations (including European Directive 2001/20/EC, US Code of Federal Regulations Title 21, and Japanese Ministry of Health, Labor, and Welfare), and with the ethical principles laid down in the Declaration of Helsinki.

10.2 Informed consent procedures

Eligible patients may only be included in the study after providing written (witnessed, where required by law or regulation), IRB/IEC-approved informed consent, or, if incapable of doing so, after such consent has been provided by a legally acceptable representative of the patient. In cases where the patient's representative gives consent, the patient should be informed about the study to the extent possible given his/her understanding. If the patient is capable of doing so, he/she should indicate assent by personally signing and dating the written informed consent document or a separate assent form. Informed consent must be obtained before conducting any study-specific procedures (i.e. all of the procedures described in the protocol). The process of obtaining informed consent should be documented in the patient source documents.

Novartis will provide to investigators in a separate document a proposed informed consent form that complies with the ICH GCP guideline and regulatory requirements and is considered appropriate for this study. Any changes to the proposed consent form suggested by

PPD [redacted]

the investigator must be agreed to by Novartis before submission to the IRB/IEC, and a copy of the approved version must be provided to the Novartis monitor after IRB/IEC approval.

Women of child bearing potential should be informed that taking the study drug may involve unknown risks to the fetus if pregnancy were to occur during the study and agree that in order to participate in the study they must adhere to the contraception requirement for the duration of the study. If there is any question that the patient will not reliably comply, they should not be entered in the study.

10.3 Responsibilities of the investigator and IRB/IEC

The protocol and the proposed informed consent form must be reviewed and approved by a properly constituted Institutional Review Board/Independent Ethics Committee/Research Ethics Board (IRB/IEC) before study start. A signed and dated statement that the protocol and informed consent have been approved by the IRB/IEC must be given to Novartis before study initiation. Prior to study start, the investigator is required to sign a protocol signature page confirming his/her agreement to conduct the study in accordance with these documents and all of the instructions and procedures found in this protocol and to give access to all relevant data and records to Novartis monitors, auditors, Novartis Clinical Quality Assurance representatives, designated agents of Novartis, IRBs/IECs, and regulatory authorities as required. If an inspection of the clinical site is requested by a regulatory authority, the investigator must inform Novartis immediately that this request has been made.

10.4 Publication of study protocol and results

Novartis assures that the key design elements of this protocol will be posted in a publicly accessible database such as clinicaltrials.gov. In addition, upon study completion and finalization of the study report the results of this trial will be either submitted for publication and/or posted in a publicly accessible database of clinical trial results.

11 Protocol adherence

Investigators ascertain they will apply due diligence to avoid protocol deviations. Under no circumstances should the investigator contact Novartis or its agents, if any, monitoring the trial to request approval of a protocol deviation, as requests to approve deviations will not be granted.

This protocol defines the study objectives, the study procedures and the data to be collected on study participants. Under no circumstances should an investigator collect additional data or conduct any additional procedures for any research related purpose involving any investigational drugs.

If the investigator feels a protocol deviation would improve the conduct of the study this must be considered a protocol amendment, and unless such an amendment is agreed upon by Novartis and approved by the IRB/IEC it cannot be implemented. All significant protocol deviations will be recorded and reported in the CSR.

11.1 Protocol Amendments

Any change or addition to the protocol can only be made in a written protocol amendment that must be approved by Novartis, Health Authorities where required, and the IRB/IEC. Only amendments that are required for patient safety may be implemented prior to IRB/IEC approval. Notwithstanding the need for approval of formal protocol amendments, the investigator is expected to take any immediate action required for the safety of any patient included in this study, even if this action represents a deviation from the protocol. In such cases, Novartis should be notified of this action and the IRB/IEC at the study site should be informed within 10 working days or less, if required by local regulations.

12 References

Abraldes JG, Tarantino I, Turnes J, et al (2003) Hemodynamic response to pharmacological treatment of portal hypertension and long-term prognosis of cirrhosis. Hepatology; Apr;37(4):902-8.

Anderson RJ, Cronin RE, McDonald KM, et al (1976) Mechanisms of portal hypertension-induced alterations in renal hemodynamics, renal water excretion, and renin secretion. J Clin Invest; Oct;58(4):964-70.

Arroyo V, Fernandez J, Ginès P (2008) Pathogenesis and treatment of hepatorenal syndrome. Semin Liver Dis; Feb;28(1):81-95.

Barthelmes D, Parviainen I, Vainio P, et al (2009) Assessment of splanchnic blood flow using magnetic resonance imaging. Eur J Gastroenterol Hepatol; Jun;21(6):693-700.

Bennett RG (2009) Relaxin and its role in the development and treatment of fibrosis. Transl Res; Jul;154(1):1-6.

Bhathal PS, Grossman HJ (1985) Reduction of the increased portal vascular resistance of the isolated perfused cirrhotic rat liver by vasodilators. J Hepatol;1(4):325-37.

Conrad KP (2010) Unveiling the vasodilatory actions and mechanisms of relaxin. Hypertension; Jul;56(1):2-9.

de Franchis R (2000) Stellate cells and the "reversible component" of portal hypertension. Dig Liver Dis; Mar;32(2):104-7.

Danielson LA, Conrad KP (2003) Time course and dose response of relaxin-mediated renal vasodilation, hyperfiltration, and changes in plasma osmolality in conscious rats; J Appl Physiol; Oct;95(4):1509-14.

Ghany M, Hoofnagle JH (2012) Approach to the patient with liver disease. In: Longo DL, Fauci AS, Kasper DL, Hauser SL, Jameson JL, Loscalzo J, (eds). Harrison's Principles of Internal Medicine, 18th ed; chapter 301; New York: McGraw-Hill. Available from <http://www.accessmedicine.com/content.aspx?aID=9132925> (accessed 16 January 2012).

Fallowfield JA, Hayden AL, Snowdon, VK, et al (2014) Relaxin modulates human and rat hepatic myofibroblast function and ameliorates portal hypertension in vivo. Hepatology;59(4):1492-1504.

PPD [redacted]

Fernández J, Navasa M, Planas R, et al (2007) Primary prophylaxis of spontaneous bacterial peritonitis delays hepatorenal syndrome and improves survival in cirrhosis. Gastroenterology; Sep;133(3):818-24.

Forrest EH, Jalan R, Redhead DN, et al (1996) Differing actions of the acute administration of propranolol and isosorbide-5-mononitrate on the portal circulation. Aliment Pharmacol Ther; Oct;10(5):795-800.

Ginès A, Escorsell A, Ginès P, et al (1993) Incidence, predictive factors, and prognosis of the hepatorenal syndrome in cirrhosis with ascites. Gastroenterology; Jul;105(1):229-36.

Ginès P, Guevara M, Arroyo V, et al (2003) Hepatorenal syndrome. Lancet; Nov 29;362(9398):1819-27.

Gouya H, Vignaux O, Sogni P, et al (2011) Chronic liver disease: systemic and splanchnic venous flow mapping with optimized cine phase-contrast MR imaging validated in a phantom model and prospectively evaluated in patients. Radiology; Oct;261(1):144-55.

Langer DA, Shah VH (2006) Nitric oxide and portal hypertension: interface of vasoreactivity and angiogenesis. J Hepatol; Jan;44(1):209-16.

Leon DA, McCambridge J (2006) Liver cirrhosis mortality rates in Britain from 1950 to 2002: an analysis of routine data; Jan 7;367(9504):52-6. Erratum in Lancet. 2006 Feb 25;367(9511):650.

McAvoy N, Leyland KA, Richards JMJ, et al (2010) Clinical hepatology: P07 Splanchnic steal in patients with liver disease: a 3T MRI study of visceral blood flow. Gut;59:A14

Munoz SJ (2008) The hepatorenal syndrome. Med Clin North Am; Jul;92(4):813-37.

Narahara Y, Kanazawa H, Taki Y, et al (2009) Effects of terlipressin on systemic, hepatic and renal hemodynamics in patients with cirrhosis. J Gastroenterol Hepatol; Nov;24(11):1791-7.

Nazar A, Pereira GH, Guevara M, et al (2010) Predictors of response to therapy with terlipressin and albumin in patients with cirrhosis and type 1 hepatorenal syndrome. Hepatology; Jan;51(1):219-26.

Ripoll C (2007) Hepatic venous pressure gradient and outcomes in cirrhosis. J Clin Gastroenterol; Nov-Dec;41 Suppl 3:S330-5.

Rosenberg AS, Worobec AS (2004) Immunogenicity Concerns of Therapeutic Protein Products. Biopharm International Dec: 34-42.

Sanyal AJ, Bosch J, Blei A, et al (2008) Portal hypertension and its complications. Gastroenterology; May;134(6):1715-28.

Schuppan D, Afdhal NH (2008) Liver cirrhosis. Lancet; Mar 8;371(9615):838-51.

Teerlink JR, Metra M, Felker GM, et al (2009) Relaxin for the treatment of patients with acute heart failure (Pre-RELAX-AHF): a multicentre, randomised, placebo-controlled, parallel-group, dose-finding phase IIb study. Lancet; Apr 25;373(9673):1429-39.

[redacted]

Valla D, Bercoff E, Menu Y, et al (1984) Discrepancy between wedged hepatic venous pressure and portal venous pressure after acute propranolol administration in patients with alcoholic cirrhosis. Gastroenterology; Jun;86(6):1400-3.

These references are available upon request.

13 Appendix 1: Clinically notable laboratory values and vital signs

Clinically notable laboratory abnormalities for selected tests based on a percent change from baseline:

Hematology

RBC count	>50% increase, >20% decrease
Hemoglobin	>50% increase, >20% decrease
Hematocrit	>50% increase, >20% decrease
WBC count	>50% increase, >50% decrease
Platelet count	>75% increase, >50% decrease

Blood Chemistry

BUN	>50% increase
Creatinine	>50% increase
CPK	>300% increase
Sodium	>5% decrease
Potassium	>20% increase, >20% decrease
Chloride	>10% increase, >10% decrease
Calcium	>10% increase, >10% decrease
Uric acid	>50% increase

14 Appendix 2: Liver event definitions and follow-up requirements

Table 14-1 Liver Event Definitions

	Definition/ threshold
**Adverse event of special interest**	
Laboratory values	ALT or AST > 3 x ULN<br>ALP > 2 x ULN<br>TBL > 1.5 x ULN
**Medically significant event (SAE)**	

[redacted]

Laboratory values	ALT or AST > 5 x ULN (with or without TBL > 2 x ULN [mainly conjugated fraction])<br>ALP > 5 x ULN (with or without TBL > 2 x ULN [mainly conjugated fraction])<br>TBL > 3 x ULN<br>Potential Hy's Law cases (defined as ALT/AST > 3 x ULN and TBL > 2 x ULN [mainly conjugated fraction] without notable increase in ALP to > 2 x ULN)
Adverse events	Any clinical event of jaundice (or equivalent term)<br>ALT or AST > 3 x ULN accompanied by general malaise, fatigue, abdominal pain, nausea, or vomiting, or rash with eosinophilia<br>Any event that links to a preferred term (PT) in the MedDRA dictionary falling under the SMQ sub-module "Drug-related hepatic disorders – severe events only"* or any "Hy's law case" PT

* These events cover the following: hepatic failure, fibrosis and cirrhosis, and other liver damage-related conditions; the non-infectious hepatitis; the benign, malignant and unspecified liver neoplasms

Table 14-2 Liver Event Follow Up Requirements

Criteria	Event type	Actions required	Follow-up monitoring
Potential Hy's Law case[a]	Medically significant	Discontinue the study drug immediately<br>Hospitalize, if clinically appropriate<br>Report to Novartis as an SAE<br>Establish causality	ALT, AST, TBL, Alb, PT, ALP and γGT until resolution[c] (frequency at investigator discretion)
**ALT or AST**			
> 8 x ULN	Medically significant	Repeat LFT within 48 hours<br>If elevation persists, discontinue the study drug immediately<br>Hospitalize if clinically appropriate<br>Report to Novartis as an SAE<br>Establish causality	ALT, AST, TBL, Alb, PT, ALP and γGT until resolution[c] (frequency at investigator discretion)
> 5 to ≤ 8 x ULN	Medically significant	Repeat LFT within 48 hours<br>If elevation persists for *more than 2 weeks*, discontinue the study drug<br>Report to Novartis as an SAE<br>Establish causality	ALT, AST, TBL, Alb, PT, ALP and γGT until resolution[c] (frequency at investigator discretion)
> 3 x ULN accompanied by symptoms[b]	Medically significant	Discontinue the study drug immediately<br>Hospitalize if clinically appropriate<br>Report to Novartis as an SAE<br>Establish causality	ALT, AST, TBL, Alb, PT, ALP and γGT until resolution[c] (frequency at investigator discretion)
> 3 to ≤ 5 x ULN (patient is	AESI	Central laboratory to report to Investigator & Novartis	Investigator discretion<br>Monitor LFT within 1 to 4

[redacted]

Criteria	Event type	Actions required	Follow-up monitoring
asymptomatic)		Repeat LFT once or twice in the week<br>If elevation persists, establish causality	weeks or at next visit
≤ 3 x ULN (patient is asymptomatic)	N/A	Repeat LFT at next visit	
**ALP (isolated)**			
> 5 x ULN	Medically significant	Repeat LFT within 48 hours<br>If elevation persists, report to Novartis as an SAE<br>Establish causality	Investigator discretion<br>Monitor LFT within 1 to 4 weeks or at next visit
> 2 to ≤5 x ULN (patient is asymptomatic)	AESI	Central laboratory to report to Investigator & Novartis<br>Repeat LFT once or twice in the weekIf elevation persists, establish causality	Investigator discretion<br>Monitor LFT within 1 to 4 weeks or at next visit
≤ 2 x ULN (patient is asymptomatic)	N/A	Repeat LFT at next visit	
**TBL (isolated)**			
> 3 x ULN	Medically significant	Repeat LFT within 48 hours<br>If elevation persists, discontinue the study drug immediately<br>Hospitalize if clinically appropriate<br>Report to Novartis as an SAE<br>Establish causality	ALT, AST, TBL, Alb, PT, ALP and γGT until resolution[c] (frequency at investigator discretion)<br>Test for hemolysis (e.g., reticulocytes, haptoglobin, unconjugated [indirect] bilirubin)
> 1.5 to ≤ 3 x ULN (patient is asymptomatic)	AESI	Central laboratory to report to Novartis<br>Repeat LFT once or twice in the weekIf elevation persists, establish causality	investigator discretion<br>Monitor LFT within 1 to 4 weeks or at next visit
≤ 1.5 x ULN (patient is asymptomatic)	N/A	Repeat LFT at next visit	
**Preferred terms**			
Jaundice	Medically significant	Discontinue the study drug immediately<br>Hospitalize the patient<br>Report to Novartis as an SAE<br>Establish causality	ALT, AST, TBL, Alb, PT, ALP and γGT until resolution[c] (frequency at investigator discretion)
"Drug-related hepatic disorders - severe	Medically significant	Discontinue the study drug<br>hospitalization if clinically appropriate	Investigator discretion

PPD [redacted]

Criteria	Event type	Actions required	Follow-up monitoring
events only" SMQ AE		Report to Novartis as an SAE<br>Establish causality	

[a] Elevated ALT/AST > 3 x ULN and TBL > 2 x ULN but with no notable increase in ALP to > 2 x ULN

[b] General malaise, fatigue, abdominal pain, nausea, or vomiting, rash with eosinophilia

[c] Resolution is defined as an outcome of one of the following: return to baseline values, stable values at three subsequent monitoring visits at least 2 weeks apart, remain at elevated level after a maximum of 6 months, liver transplantation, and death.

15 Appendix 3: Child-Pugh Clinical Assessment Score

Factor	Unit	Score		
		1	2	3
Serum bilirubin	μmol/L	<34	34-51	>51
	mg/dL	<2.0	2.0-3.0	>3.0
Serum albumin	g/L	>35	30-35	<30
	g/dL	>3.5	3.0-3.5	<3.0
Prothrombin time	Seconds prolonged	<4	4-6	>6
	INR*	<1.7	1.7-2.3	>2.3
Ascites	NA	None	Easily controlled	Poorly controlled
Hepatic encephalopathy	NA	None	Minimal	Advanced

*International normalized ratio

The Child-Pugh score is calculated by adding the scores of the five factors and can range from 5-15 (Ghany and Hoofnagle 2012).

[redacted]

16 Appendix 4: Sample log table

Table 16-1 Sample collection time points for pharmacokinetic (PK), immunogenicity (IG) and biomarker analyses from all patients receiving serelaxin study treatment (portal vein blood sampling in part B only)

Period	Visit No.	Day	Timing	PK Sample no.	PK Collection no.	IG Sample no.	Peripheral blood EDTA sample no.	Peripheral blood Li-Hep sample no.	Urine Sample no.	Portal vein blood* EDTA sample no.	Portal vein blood* Li-Hep sample no.
Screening	1	D-7									
Treatment	2	D1	0 min (prior to serelaxin administration)	101	1	201	301	401	501	601	701
			60 min (just prior to decreasing the serelaxin infusion rate)	102	1						
			120 min (after starting the serelaxin infusion)	103	1		302	402	502**	602	702
			60 min after stopping the serelaxin infusion (end of recovery)	104	1						
End of study	3 (777)	D29		105	1	202					
Unscheduled	999	-		1101	1	1201					

*In part B only. The 120 min portal vein blood samples will be collected between the final (120 min) PVP and the final IVCP measurement.

**As close as possible to the 120 min time point. For operational reasons this will be the last 120 min assessment and can take place in part A2 only after completion of second MRA and after cessation of the serelaxin infusion (in part A2 and B).

[redacted]
